# Radiocesium levels in contaminated forests has remained stable, even after heavy rains due to typhoons and localized downpours

**DOI:** 10.1038/s41598-020-75857-1

**Published:** 2020-11-05

**Authors:** Yasuyuki Taira, Masahiko Matsuo, Takumi Yamaguchi, Yumiko Yamada, Makiko Orita, Noboru Takamura

**Affiliations:** Department of Global Health, Medicine and Welfare, Atomic Bomb Disease Institute, Nagasaki University Graduate School of Biomedical Sciences, Nagasaki University, 1-12-4 Sakamoto, Nagasaki City, Nagasaki 852-8523 Japan

**Keywords:** Environmental sciences, Natural hazards, Risk factors

## Abstract

In recent years, Japan has suffered serious damage due to natural disasters such as earthquakes, heavy rains due to tropical storms (typhoons) and localized downpours. To assess the chronological changes in the attenuation of external exposure doses and environmental radiation contamination due to the rainfall associated with typhoons and heavy rains during October to December 2019 in Fukushima, we measured environmental radiation levels in forest areas along the Mt Okura hiking trail in Tomioka Town, Fukushima Prefecture, near the Fukushima Daiichi Nuclear Power Station. We confirmed that (1) current ambient dose rates of 0.38–0.95 μSv/h in most forest areas were 79.9–84.7% higher than in residential areas; (2) the number of sites along the hiking trail where ^137^Cs was detected was limited (1.1–4.7%); and (3) individual dose rates of 0.21–0.34 μSv/h were lower than ambient dose rates. These findings suggest that radiocesium has remained stable in natural forests that have not been decontaminated even though current levels are low, despite the occurrence of heavy rainfall associated with Super Typhoon Hagibis in 2019 and localized downpours. Hiking while managing exposure to environmental contamination using a personal dosimeter may be the safest model for spending time of leisure activities.

## Introduction

Nine years have passed since the severe nuclear accident at the Fukushima Dai-ichi Nuclear Power Station (FDNPS), which was severely damaged by the tsunami associated with the magnitude 9.0 Great East Japan Earthquake^1,2^. The accident resulted in the release of radionuclides such as iodine-131 (^131^I; half-life (t_1⁄2_) = 8 days), cesium-134 (^134^Cs; t_1⁄2_ = 2.1 y) and cesium-137 (^137^Cs; t_1⁄2_ = 30.1 y) into the atmosphere, which were eventually deposited on the surrounding terrestrial and marine environments^1,2^. Following the accident, decontamination was conducted in residential areas, farmlands, forests adjacent to residential areas, and roads within the evacuation area around the FDNPS; however, the decontamination activities, which were completed on March 19, 2018, excluded the Difficult-to-Return Zones^3^. In addition, since 68% of Fukushima Prefecture is covered by forests and forest areas in the prefecture accumulated 72% of the total atmospheric input of ^137^Cs, forest ecosystems contaminated with ^137^Cs may increase exposure of the local population to radiation by elevating ambient dose rates (external exposure) and through the consumption of contaminated forest products (internal exposure) over the coming decades^4–6^.

**Figure 1:**
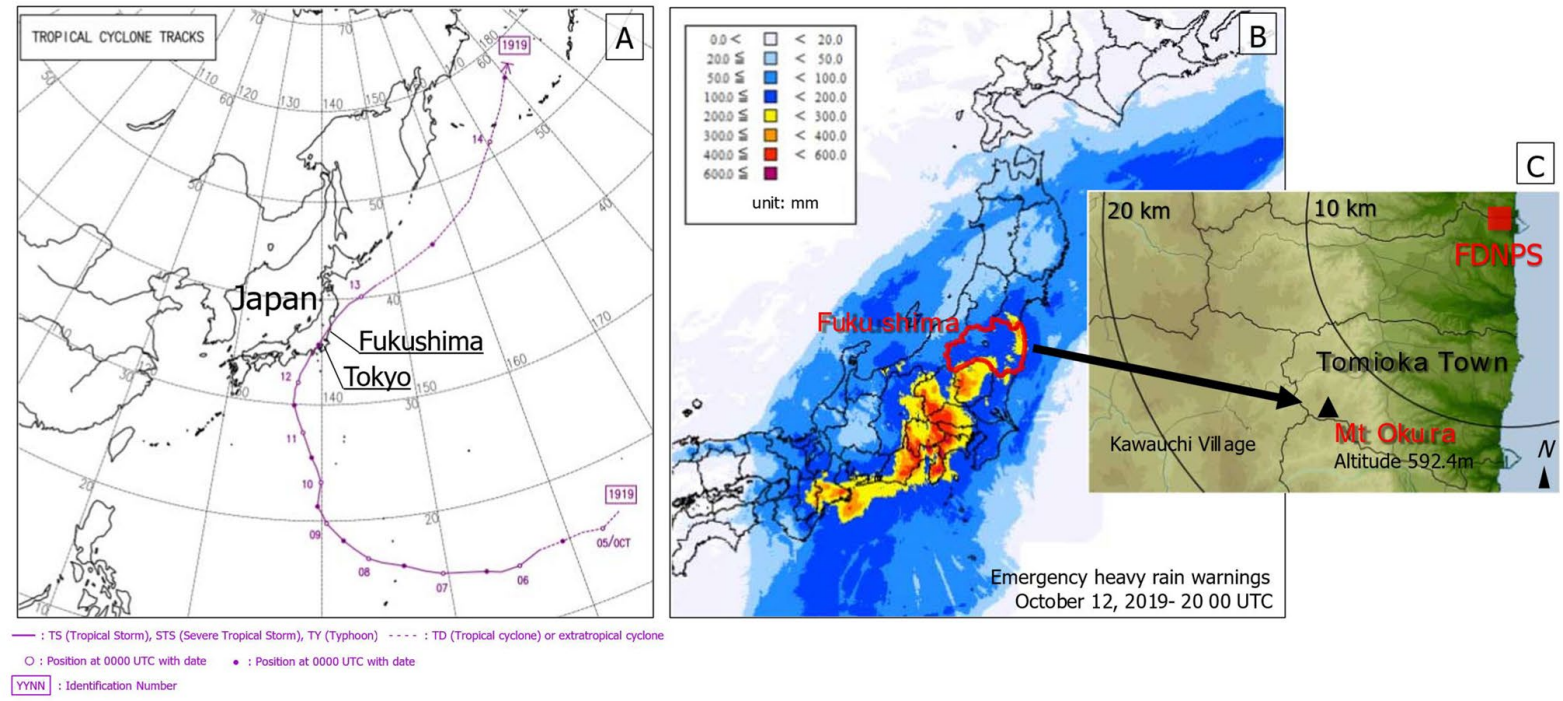
(**A**) Path of Typhoon Hagibis in 2019, (**B**) precipitation after 48 h, and (**C**) location of Mt Okura in Tomioka Town, Fukushima Prefecture, Japan. (**A**,**B**) are reprinted and modified from Regional Specialized Meteorological Centres Best Track Data (Graphics) published in 2019 (https://www.jma.go.jp/jma/jma-eng/jma-center/rsmc-hp-pub-eg/bstve_2019_m.html) and a JMA Report on Typhoon 19 (http://www.data.jma.go.jp/obd/stats/data/bosai/report/2019/20191012/jyun_sokuji20191010-1013.pdf), under a CC BY license, with permission; (**C**) shows a map produced by the first author (Y.T.) using GREEN MAP III software (Tokyo Shoseki Co., Ltd, Tokyo, Japan; https://www.tokyo-shoseki.co.jp/company_english/philosophy.html). Reprinted from GREEN MAP III under a CC BY license with permission from Tokyo Shoseki Co., Ltd.; original copyright 2003.

In recent years, Japan has suffered from natural disasters such as earthquakes, heavy rains due to tropical storms, and localized downpours^7^. From the evening of October 12, 2019 to the following morning, Typhoon Hagibis (Typhoon 19), the most powerful typhoon to hit Japan in recorded history, affected extensive areas of Honshu Island, including Tokyo and Fukushima (Fig. 1)^8^. According to the Japan Meteorological Agency (JMA), the typhoon was classified as “very strong”, which is the highest category on Japan’s typhoon scale and is equivalent to a Category 5 hurricane^8,9^. On October 12, atmospheric pressures as low as 950 hPa and maximum wind speeds reaching 70 m/s were recorded^8,9^. The total rainfall measured over a 72-h period ranged from 750 to 1000 mm in some regions^8,9^. The extraordinary amount of rainfall that fell over such a short period resulted in a rapid rise in river levels and extensive flooding. According to the Ministry of Land, Infrastructure, Transport and Tourism, Japan, 66 embankments along 47 major rivers collapsed, and 203 rivers overflowed their banks, as of October 15, 2019^8^. In addition to the accumulated radiocesium in forests and rivers, the nuclides in radioactive waste that were being stored temporarily in Fukushima Prefecture, entered the natural environment in runoff resulting from heavy rains^10,11^. The forest area requiring decontamination is extremely large and will require considerable financial resources to remediate. Consequently, further discussions considering the cost-effectiveness and priorities of forest decontamination policies are necessary^3,12,13^.

Tomioka Town in Fukushima Prefecture is located within a 20-km radius of the FDNPS. On April 1, 2017, the Japanese government declared that the residents who previously resided in approximately 88% of the town could return to their homes as air dose rates were at acceptable levels (< 20 mSv/y)^14,15^. Although 3 years have passed since the government declaration, as of March 1, 2020, a total of 2317 of the 11,422 evacuees of Tomioka Town (20.3%) currently reside outside Fukushima Prefecture and 9105 (79.7%) reside elsewhere within the prefecture; the number of residents who have returned to Tomioka Town is still extremely low, at 1212 (9.6%)^16^. Since one of the reasons for the low number of returning residents is considered to be anxiety related to radiation exposure through the consumption of local foodstuffs, scientific investigations are required in order to reassure the public that it is safe to return to the town^14,17–19^.

Previously, we showed that the external exposure dose rates in the residential zone (i.e., areas to which previous residents returned) of Tomioka Town were decreasing^20–23^. However, the external exposure risk in recreation areas, such as the hiking trails in the forests surrounding the town, has not been evaluated sufficiently. Therefore, in the present study, we investigated ambient dose equivalent rates (ambient dose rates), artificial radionuclide spectra, and individual doses in order to evaluate radiation exposure doses (external exposure doses) and the magnitude of environmental contamination in the forest areas surrounding Tomioka Town, Fukushima Prefecture. Moreover, we examined chronological changes in ambient dose rates due to possible attenuation by heavy rainfall events along the Mount Okura hiking trail in Tomioka Town.

## Results

**Figure 2:**
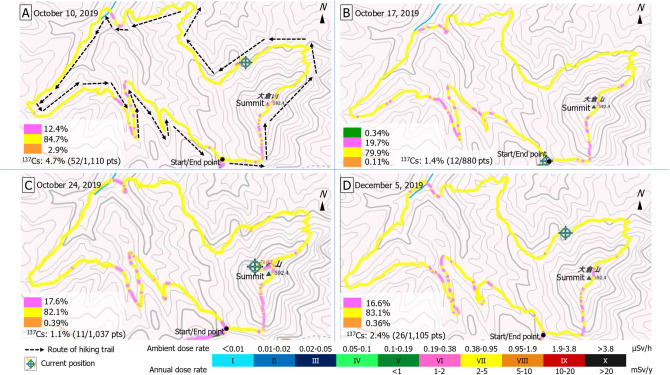
Radiation maps showing color-scaled ambient dose rates along the Mt Okura-hiking trail. The map data collected using a walking survey conducted in October and December 2019 is produced by the first author (YT) using GIS software. Map reprinted with permission under a CC BY license (No. 61-G-081) from Shobunsha Publications, Inc., Tokyo, Japan. https://www.mapple.co.jp/en/; originally copyrighted in 2017 by Chiyoda Technology Corp., Tokyo, Japan.

The frequency distributions of the ambient dose rates (H*(10)) and individual doses (*Hp*(10)) along the Mt Okura hiking trail in Tomioka Town are shown in Table 1. The median ambient dose rates (H*(10)) and external exposure rates (annual estimated dose rates) were 0.45–0.48 µSv/h and 3.9–4.2 mSv/y, respectively. Radiation maps for the walking survey along the Mt Okura hiking trail are shown in Fig. 2. In the radiation mapping component of the study, zone VII (0.38–0.95 μSv/h), zone VI (0.19–0.38 μSv/h) and zone VIII (0.95–1.9 µSv/h) occupied 79.9–84.7%, 12.4–19.7% and 0.11–2.9% of all measurement points, respectively (Fig. 2). Additionally, the dose-forming artificial radionuclides, ^134^Cs and ^137^Cs, were only prevalent at some measurement points (Supplementary S1). The number of measurement points where ^137^Cs could be detected compared to all measurement points was in the range 1.1–4.7% during October to December 2019 (Fig. 2). Moreover, individual doses (*Hp*(10)) along the hiking trail would be in the range 0.21–0.34 μSv/h and annual estimated dose rates were 0.10–0.16 mSv/y, respectively (Table 1).

**Table 1:** Ambient dose rates along the hiking trail on Mt Okura in Tomioka Town, Fukushima Prefecture, during October to December 2019 (see Supplementary S1).

Date (dd/mm/yy)	Device	Average (µSv/h)	Range (µSv/h)	Median (µSv/h)	External exposure dose (mSv/y)	Proportion of ^137^Cs sites (% (pts/pts))
10/10/19	RADI-PROBE	0.51	0.19–1.1^a^	0.48 (0.67)^b^	4.2^c^	4.7 (52/1110)^d^
	D-SHUTTLE	0.34^e^			0.16^f^	–
17/10/19	RADI-PROBE	0.47	0.17–0.96	0.45 (0.63)	3.9	1.4 (12/880)
	D-SHUTTLE	0.21			0.10	–
24/10/19	RADI-PROBE	0.47	0.20–1.1	0.45 (0.60)	3.9	1.1 (11/1037)
	D-SHUTTLE	N/A			N/A	–
05/12/19	RADI-PROBE	0.49	0.19–1.1	0.47 (0.64)	4.1	2.4 (26/1105)
	D-SHUTTLE	0.23			0.11	–

^a^Minimum–maximum (maximum values were measured at the same location).

^b^90th percentile.

^c^Estimated annual dose rate (µSv/h × 24 h × 365 d × 0.001) based on median dose rate using the RADI-PROBE system.

^d^Proportion of measurement sites where ^137^Cs was detected using a RADI-PROBE. The values inside the brackets shows monitoring points where ^137^Cs was detected per all monitoring points.

^e^Individual dose rate for the entire time spent hiking measured using a D-SHUTTLE dosimeter (*Hp*(10)).

^f^Estimated annual dose rate assuming that the average leisure time of Japanese on weekends is 10 h (i.e., 480 h/year) measured using a D-SHUTTLE dosimeter.

**Figure 3:**
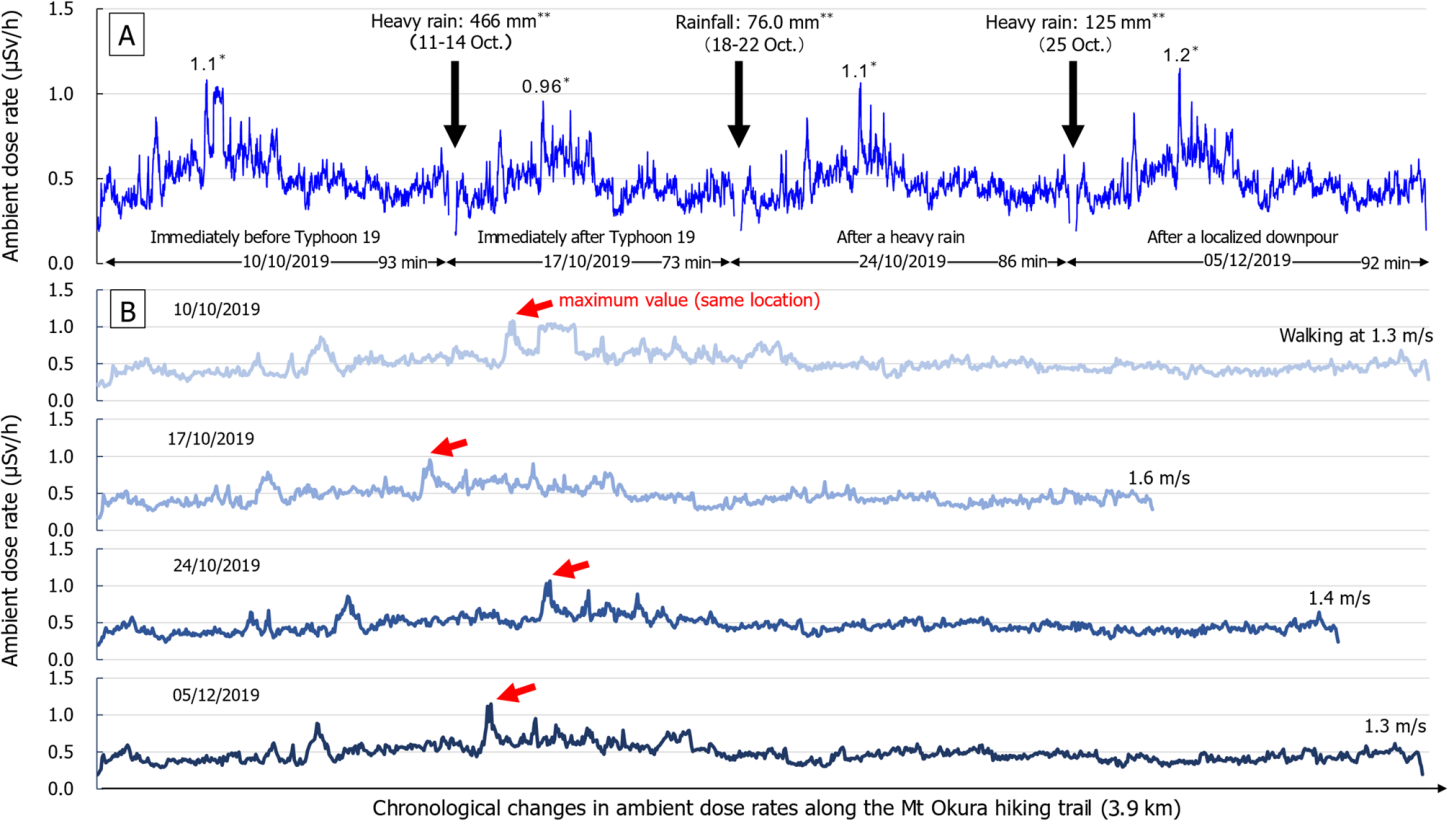
Chronological changes in ambient dose rates along the Mt Okura hiking trail. (**A**) Whole variation in ambient dose rates. The data were obtained by a walking survey conducted during October to December 2019 using a RADI-PROBE system. The single asterisk shows maximum µSv/h values. Maximum values were consistently measured at the same locations (Supplementary S1). The double asterisk shows a heavy rainfall events such as typhoon or downpour and precipitation measured at a weather station in Kawauchi Village near Mt Okura. The 466 mm of precipitation due to Typhoon 19 during October 11 to 14, 2019 was equivalent to three times the monthly average (JMA, Tokyo, Japan. Available from: https://www.data.jma.go.jp/obd/stats/etrn/view/daily_a1.php?prec_no=36&block_no=1129&yeye=2019&month=10&day=&view=); (**B**) Separate variation in ambient dose rates. Red arrows show the same location on the hiking trail (showed the maximum value). Walking speed except for breaks was in the range 1.3–1.6 m/s.

To evaluate chronological changes in ambient dose rates due to heavy rains, moreover, we examined the variation in ambient dose rates along the Mt Okura hiking trail (Fig. 3 and Supplementary S1). The maximum values of ambient dose rates along the hiking trail were 0.96–1.2 µSv/h at the same location (Fig. 3).

## Discussion

According to monitoring information collected by national and local authorities, the prevalent dose-forming artificial radionuclides from various samples in offsite areas around the FDNPS were mainly ^134^Cs and/or ^137^Cs^14,15^. Although the proportion of sites where ^137^Cs was detected was limited (1.1–4.7%) in the present study, maximum ambient radiocesium dose rates were detected at locations where walking speed was in the range 1.3–1.6 m/s (Fig. 3 and Supplementary S1). We also measured the distribution of ambient dose rates in forests around Mt Okura using the RADI-PROBE in 2018^24^. The median ambient dose rate was 0.49 μSv/h (0.19–1.3 μSv/h) along the hiking trail and 0.49 μSv/h (0.14–1.2 μSv/h) off the hiking trail. However, radiocesium was only detected at 2.4% of sites along the hiking trail and at 12.9% sites off the hiking trail^24^. When temporal variations in ambient dose rates in the difficult-to-return to areas of Tomioka Town were evaluated using the RADI-PROBE (car-based survey) during 2018–2019, we confirmed that the dose rates in the area had decreased dramatically due to decontamination efforts^25^. ^137^Cs was detected at 3.8% to 93% of sites using the RADI-PROBE^25^, while the dose-forming artificial radionuclides ^134^Cs and ^137^Cs were only prevalent at some measuring sites (Supplementary S1)^24^.

In 2017, we reported that median ambient dose rates around the homes of the residents who had returned to areas where the evacuation order had been lifted in Tomioka Town were 0.20–0.34 µSv/h (1.7–3.0 mSv/y) and the additional radiation exposure dose was estimated at 1.6 mSv/y based on the guidelines from the Ministry of the Environment, Japan^20^. In 2018, we estimated that the median ambient dose rate along the Mt Okura hiking trail was 0.49 μSv/h, and that the individual dose was 0.44 μSv/h (0.42 mSv/y) (Supplementary S1)^24^. In the present study, the median ambient dose rates (0.45–0.48 µSv/h, 3.9–4.2 mSv/y) were higher along the hiking trail than in the residential areas where the evacuation order had been lifted in Tomioka Town. Moreover, the reduction of ambient dose rates was calculated using dose conversion coefficients under the assumption that the depth profile of radiocesium did not change significantly over time and the initial radioactivity of ^137^Cs was 8.8 PBq^26^. In the present study, the reduction of radiocesium was estimated to be only 0.35% from October to December 2019 (Supplementary S2)^26,27^. Thus, the median ambient dose rates along the Mt Okura hiking trail remained relatively stable in 2019^20,24^.

Rain events during wet weather have been shown to enhance remediation in human-affected areas (paddy fields, farmland and urban areas) and road surface deposits containing ^137^Cs^28,29^. The ^137^Cs that was deposited in the Tomioka river basin near the FDNPS was transported downstream to the ocean after binding to soil particles in river runoff^30^. During the rainy season (May to July) and typhoon season (August to October) in Japan, large amounts of runoff erode unstable stream banks. Compared to sites like Chernobyl, this annual increase in river runoff increased the natural attenuation of radioactive contamination in Fukushima^31^. Differences in weather and geographic conditions have had a marked influence on the rate of natural attenuation processes in Chernobyl and Fukushima^31^. Precipitation differs substantially, with annual averages of about 1500 mm in Fukushima according to the JMA and about 600 mm in Chernobyl^31^. Additionally, the watersheds in Fukushima are hilly with steep slopes^31^. The effective dispersion coefficient for contaminated soils in Fukushima has been shown to be higher than in Chernobyl, and the vertical migration of ^137^Cs in soils showed even greater variability in Chernobyl^31,32^. On the other hand, several reports have suggested that the radiocesium fallout in reservoirs, mineral soil layers and litter substrates has been immobilized in the forested areas of the Fukushima region and that these nuclides are bioavailable in forest ecosystems^33,34^. These scenarios show that radiocesium nuclides constitute a potential long-term source of contaminated particulate matter that will require diligent management for the foreseeable future^33–35^. Therefore, it is suggested that the ambient dose rates, including radiocesium in forest areas along the Mt Okura hiking trail, were stable in the present study.

In the present study, the ambient and cumulative dose rates on October 17, 2019 decreased temporarily. This decrease may have been attributable, not to runoff resulting from a heavy rainfall event (Typhoon 19), but due to the shielding effect that rainwater has by covering the surface soil and forest vegetation (Fig. 3 and Supplementary S3)^36,37^. In other words, ambient dose rates along the Mt Okura hiking trail did not change significantly and the natural attenuation of ambient radiation doses in forests, including that attributed to radiocesium, is expected to decrease slower than the theoretical decay rate^36^. These findings indicate that the radiocesium levels in the forests around Fukushima (including the hiking trail) have remained stable, despite the heavy rains associated with tropical storms such as Typhoon Hagibis and localized downpours^33,34^. Nonetheless, individual doses have decreased slightly from 0.44 μSv/h in 2018 to 0.21–0.34 μSv/h in 2019^24^. Moreover, the estimated annual doses (*Hp*(10)) of 0.10–0.16 mSv/y that were recorded along the hiking trail are considered to be sufficiently low (< 1 mSv/y)^38^. In general, individual doses (*Hp*(10)) are much lower than the ambient doses (H*(10)) because of the shielding effect of obstacles, such as buildings^39^. The dose to people should be estimated carefully, and should therefore consider such effects and/or time factors^39^. Therefore, it is possible that individual doses (*Hp*(10)) may not be affected by the surroundings as strongly as ambient doses (H*(10)).

Limitations of the present study are that the bias associated with the D-SHUTTLE dosimeter positions (investigator) and temperature may be significant; in the present study, the temperature was observed to vary from 17 to 19 °C in October 2019, and from 10 to 13 °C in December 2019^40,41^. In addition, we did not characterize the soils along the Mt Okura hiking trail, although the findings of our previous study showed that dose-forming artificial radiocesium was prevalent in all surface soil samples (0–10 cm) collected in Tomioka Town in 2017^20^. However, in the present study, we used the RADI-PROBE for high and sensitive gamma-ray detection (typically 1400 cps per µSv/h for ^137^Cs)^24,25,42^. Further investigations on individual doses and ambient doses under several weather conditions are needed.

Before the FDNPS accident, the inhabitants of Fukushima enjoyed the blessings of nature by engaging in outdoor activities such as green tourism, harvesting wild plants and mushrooms, freshwater fishing and hiking^20,24,43–45^. However, the number of visitors to the mountains in the year after the accident declined by more than 25% in areas close to the FDNPS and by 40% in areas farther away, and the recovery rate in terms of the number of visitors to the mountains in areas closer to the FDNPS is lower^44^. The effectiveness of the decontamination efforts in the forest zone are dependent upon the specific characteristics of the site, the timing of the measures, and subsequent efforts to remove newly formed litterfall every year^36,46^. These challenges demonstrate the importance of monitoring and assessing the decontamination efforts in the Fukushima restoration zone, particularly in forest areas near residential areas^24,36^.

## Conclusions

In summary, to evaluate external exposure doses and the magnitude of environmental contamination, ambient dose rates, radiocesium spectra derived from the FDNPS, and individual doses, were investigated in forest areas surrounding Tomioka Town, Fukushima Prefecture, from October to December 2019. Additionally, chronological changes in ambient dose rates due to possible attenuation by heavy rainfall events were examined along the Mt Okura Hiking Trail in Tomioka Town. It was confirmed that (1) current ambient dose rates of 0.38–0.95 μSv/h in most forest areas were 79.9–84.7% higher than in residential areas; (2) the number of sites along the hiking trail where ^137^Cs was detected was limited (1.1–4.7%) at the same location; and (3) individual dose rates of 0.21–0.34 μSv/h were lower than ambient dose rates. These findings suggest that radiocesium has remained stable in natural forests that have not been decontaminated, despite the occurrence of heavy rainfall associated with Super Typhoon Hagibis in 2019 and localized downpours. However, the safety of residents’ leisure activities, such as hiking, could be guaranteed if exposure to environmental contamination was monitored using a radiation dosimeter.

## Materials and methods

### Study site

**Figure 4:**
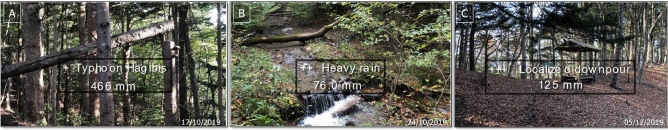
Photos (**A**–**C**) show Mt Okura hiking trail.

The FDNPS (37°25′ N, 141°02′ E) is located on the east coast of Honshu Island, Japan, approximately 200 km northeast of Tokyo. Mt Okura (37°34′ N, 140°93′ E) in Tomioka Town (public office: 37°20′ N, 141°0′ E) is located 13 km southwest of the FDNPS (Figs. 1 and 4). In the present study, we measured ambient dose rates and analyzed the spectra of artificial radionuclides (mainly radiocesium) along the Mt Okura trail (3.9 km) using a walking survey methodology with a RADI-PROBE system (RADI-PROBE; Chiyoda Technology Corp., Tokyo, Japan) and a personal cumulative dosimeter (D-SHUTTLE; Chiyoda Technology Corp.) during October and December in 2019.

### Measurement of radiation dose rate: radiation mapping and chronological changes in radiocesium spectra

The RADI-PROBE is a data acquisition system for vehicle-based and/or walking surveys at sites of radiological accidents^42^. The system consists of several radiation detectors, a Global Positioning System (GPS) receiver, a USB camera, a mobile modem and a laptop computer. This system can record radionuclide spectra and dose rates with date, time and location information, as well as photographs at defined time intervals. The software shipped with the system has a graphical interface that shows gamma-ray energy spectra and a map with color-scaled ambient dose equivalent rates. Temporal variation in the ambient dose equivalent rate is also displayed on the graphical interface. Snapshots taken by a USB camera affixed to the front of the vehicle are used to capture the surrounding environment and weather. In the present study, the ambient dose rates were measured, and geographic coordinates and a photograph were recorded automatically 5-s intervals, with spectrum segments recorded at 0.2-s intervals. Based on the data provided by the Ministry of the Environment, real-time maps with color-scaled ambient dose rates divided into 10 categories (zone I to X) were created, as follows: (Fig. 2)^20^.

**Table:** 

Category (zone)	Ambient dose rate in μSv/h	Annual dose rate in mSv/y
I	< 0.01	
II	0.01–0.02	
III	0.02–0.05	
IV	0.05–0.1	
V	0.1–0.19	< 1
VI	0.19–0.38	1–2
VII	0.38–0.95	2–5
VIII	0.95–1.9	5–10
IX	1.9–3.8	10–20
X	> 3.8	> 20

Gamma-ray detection obtained using the RADI-PROBE was performed using a large thallium-doped cesium iodide scintillator (HDS-101GN, SN; 18001546, Mirion Technologies, Inc., Japan) with high sensitivity (typically 1400 cps per µSv/h for ^137^Cs), as well as a handheld radiation detector. The measurable range of gamma-ray energy for the multichannel analyzer with 512 channels was 30 keV to 6 meV and gamma-ray energy spectra were generated as outputs (Fig. 2). The detected energy peaks for radiocesium (^134^Cs and ^137^Cs) registered in the nuclear library (i.e., detected net count values) and their associated confidence intervals were obtained for the region of interest (with levels 1–10 used as reference values)^24,42^. From the data obtained using this system, we analyzed levels of ^137^Cs (t_1⁄2_ = 30.1 y) detected along the Mt Okura hiking trail and prepared radiation maps. We also evaluated whether natural disasters such as typhoons, heavy rains and floods could affect ambient dose rates.

### Individual doses

The D-SHUTTLE for *Hp*(10) is a light and compact dosimeter designed to measure the exposure of individuals to gamma rays (cumulative doses)^40^. The device incorporates a semiconductor equipped with an error-detection prevention function and a shock sensor. The dose range for gamma-rays that can measured using the device is 0.1 µSv to 99.9999 mSv (total cumulative dose) with doses recorded every hour. We measured actual external doses by affixing the device on the chest of an investigator (the same person) hiking along the trail. Assuming that the average leisure time (sports, hobbies and amusements) of Japanese on weekends is 10 h, we also estimated external exposure doses based on the Survey on Time Use and Leisure Activities, Statistics Bureau, Ministry of Internal Affairs and Communications, Japan^47^.

## Supplementary information

Supplementary Information.
