# Supplementary material for: Radiocesium levels in contaminated forests has remained stable, even after heavy rains due to typhoons and localized downpours
Source: Sci Rep. 2020 Nov 5;10:19215. doi: 10.1038/s41598-020-75857-1 (PMC7645623; doi:10.1038/s41598-020-75857-1)
Supplement: Supplementary file 1 — Supplementary Information. [file 41598_2020_75857_MOESM1_ESM.pdf]

Supplementary Information

Radiocesium levels in contaminated forests has remained stable, even after heavy rains due to typhoons and localized downpours

Yasuyuki Taira*, Masahiko Matsuo, Takumi Yamaguchi, Yumiko Yamada, Makiko Orita and Noboru Takamura

Department of Global Health, Medicine and Welfare, Atomic Bomb Disease Institute, Nagasaki University, Nagasaki, Japan

*Corresponding author: Yasuyuki Taira
Department of Global Health, Medicine and Welfare, Atomic Bomb Disease Institute, Nagasaki University
1-12-4 Sakamoto, Nagasaki city, Nagasaki 852-8523, Japan
Phone: +81-95-819-7171
Fax: +81-95-819-7172
E-mail: y-taira@nagasaki-u.ac.jp

Contents

S1. Sites where ^{134}C and/or ^{137}Cs could be detected compared to all of the sites sampled using a Radi-probe system in 2018–2019 (raw data files).

Data sheets were prepared using Microsoft Excel (**Table S1-1**, **S1-2**, **S1-3**, **S1-4** and **S1-5**) in **Supplementary S1** (pp.4-8).

S2. Statistical analysis of ambient dose rates performed using data collected along the Mt Okura hiking trail in Tomioka Town, Fukushima Prefecture using a Radi-probe system in 2018–2019 (raw data files).

Data sheets prepared using Microsoft Excel (**Table S1-1**, **S1-2**, **S1-3**, **S1-4** and **S1-5**) in **Supplementary S1** (pp.4-8).

S3. Radiation maps showing color-scaled ambient dose rates measured along the Mt Okura hiking trail in Tomioka Town, Fukushima Prefecture using a Radi-probe system in 2018–2019.

See maps (**Fig S1-1**, **S1-2**, **S1-3**, **S1-4** and **S1-5)*** in **Supplementary S1** (pp.9-13).

S4. Chronological changes in ambient dose rates measured along the Mt Okura hiking trail in 2018–2019.

See **Fig S1-6**, **S1-7**, **S1-8** and **S1-9** in **Supplementary S1** (pp.14-17).

S5. Estimated reduction of the ambient dose rates within the Mt Okura hiking trail.
See **Table S2-1** in **Supplementary S2** (pp.18).

S6. Ambient dose rates and chronological changes in ambient dose rates measured using a SPRDJ** personal radiation detector (RadEye, ThermoFisher Scientific, Waltham, MA) to corroborate Radi-probe data in 2018–2019.

See **Table S3-1**, **S3-2**, **S3-3**, **S3-4**, **S3-5** and **Fig S3-1** in **Supplementary S3** (pp.19-24).

*These maps were produced by the first author (Y.T.) and modified by using Excel software, from the map obtained by the walking survey using the RADI-PROBE system made during October 2018 and December 2019 (GIS software: Shobunsha Publications, Inc., Tokyo, Japan. https://www.mapple.co.jp/en/. The RADI-PROBE system: Chiyoda Technology Corp., Tokyo, Japan. http://www.c-technol.co.jp/eng). Reprinted from the map software for the RADI-PROBE system under a CC BY license, with permission (No. 61-G-081) from Shobunsha Publications, Inc., Tokyo, Japan; original copyright 2017 and Chiyoda Technology Corp., Tokyo, Japan.

**The RADEYE SPRDJ is a small, highly sensitive, portable spectroscopic device used for detecting and localizing sources of gamma- and neutron-rays [1-2]. The device can distinguish between gamma-rays emitted by artificial radionuclides and natural background sources. In addition, energy-compensated dose rates of gamma-ray (Sv/h) and gamma-ray exposure can be measured at specific time intervals. The device incorporates a single CsI (Tl) cesium iodide scintillator equipped with a miniature silicon photomultiplier which

allows for the detection of very low radiation levels; gamma radiation can be measured in the range 0.01 μSv/h to 250 μSv/h or 60 keV to 1.0 MeV. The energy resolution is 7.5% (662 keV) and 1024 ch (40 keV-3.0 MeV). A total of 41 radionuclide species can be registered with this device, including ^{134}Cs and ^{137}Cs.

In the present study, we conducted a preliminary evaluation of the chronological changes in ambient dose rates at 60-second intervals using an SPRDJ dosimeter to assess the effects of typhoons, heavy rain and floods on the attenuation of radioactivity levels on Mt Okura. Because the preliminary data obtained using the SPRDJ dosimeter showed dose-rate changes that were comparable to those obtained using a Radi-probe system (RADI-PROBE; Chiyoda Technology Corp., Tokyo, Japan), we consider that the data presented in the present study are accurate.

References:

[1] RadEye SPRD-ER Personal Radiation Detector, Geiger Counters for Radiation Detection, Thermo Fisher Scientific Messtechnik GmbH, Erlangen, Germany. Available from: https://www.thermofisher.com/order/catalog/product/425082542

[2] Chiyoda Technol Corporation, Tokyo, Japan. Available from: http://www.c-technol.co.jp/eng

Table S1-1 Data sheet for October 10, 2018

Measuring points	<0.19	0.19-0.38	0.38-0.95	0.95-1.9	1.9-3.8	>3.8	μSv/h	Median	0.49	μSv/h
1016	1	81	928	6	0	0			4.3	mSv/y
	Ratio	Ratio	Ratio	Ratio	Ratio	Ratio		90th percentile	0.68	
	0.10	7.97	91.34	0.59	0.00	0.00	%	Average	0.52	
Detected ^{137}Cs	2.4	%						Min	0.19	
								Max	1.3	

Date	Time	Latitude		Longitude		Altitude		Dose rate (μSv/h)	Temperature	Detected ^{137}Cs	Levels 1-10	Detected ^{134}Cs	Levels 1-10
2018/10/25	14:35:41	3720.165	N	14055.5877	E	509.7	M	0.18758	19	0	0	0	0
2018/10/25	14:35:46	3720.1653	N	14055.5894	E	509.7	M	0.19364	19	0	0	0	0
2018/10/25	14:35:51	3720.1653	N	14055.5906	E	509.3	M	0.21245	19	0	0	0	0
2018/10/25	14:35:56	3720.1665	N	14055.592	E	509.6	M	0.26113	19	0	0	0	0
2018/10/25	14:36:01	3720.1675	N	14055.5922	E	511	M	0.30711	19	0	0	0	0
2018/10/25	14:36:06	3720.169	N	14055.5917	E	512.8	M	0.47522	19	0	0	0	0
2018/10/25	14:36:11	3720.1709	N	14055.5924	E	513.8	M	0.45256	19	0	0	0	0
2018/10/25	14:36:16	3720.1728	N	14055.5937	E	515.1	M	0.40764	19	0	0	0	0
2018/10/25	14:36:21	3720.1737	N	14055.5946	E	516	M	0.41469	19	0	0	0	0
2018/10/25	14:36:26	3720.1744	N	14055.5962	E	516.7	M	0.43977	19	0	0	0	0
2018/10/25	14:36:31	3720.1746	N	14055.5973	E	518.6	M	0.37128	19	0	0	0	0
2018/10/25	14:36:36	3720.1754	N	14055.5985	E	519.4	M	0.40388	19	0	0	0	0
2018/10/25	15:03:54	3720.3491	N	14055.6522	E	515.2	M	0.90681	19	0	0	0	0
2018/10/25	15:03:59	3720.3482	N	14055.6519	E	514.9	M	0.93081	Ind.	137Cs	4	134Cs	1
2018/10/25	15:04:04	3720.3479	N	14055.6508	E	514.7	M	1.2709	Ind.	137Cs	5	134Cs	1
2018/10/25	15:04:09	3720.3479	N	14055.6504	E	514.5	M	1.0115	Ind.	137Cs	5	134Cs	1
2018/10/25	15:04:14	3720.3482	N	14055.6505	E	512.8	M	1.1999	Ind.	137Cs	6	134Cs	1
2018/10/25	15:04:19	3720.3494	N	14055.6506	E	510.8	M	1.2706	Ind.	137Cs	7	134Cs	2
2018/10/25	15:04:24	3720.3509	N	14055.6496	E	510.8	M	0.87633	Ind.	137Cs	7	134Cs	2
2018/10/25	15:04:29	3720.3541	N	14055.648	E	510.9	M	0.93086	Ind.	137Cs	8	134Cs	2
2018/10/25	15:04:34	3720.3569	N	14055.6448	E	510.7	M	0.89711	Ind.	137Cs	8	134Cs	2
2018/10/25	15:04:39	3720.3591	N	14055.6426	E	510.5	M	0.81411	Ind.	137Cs	8	134Cs	2
2018/10/25	15:04:44	3720.3597	N	14055.6405	E	509.9	M	0.70871	19	0	0	0	0
2018/10/25	15:04:49	3720.3608	N	14055.6379	E	510.3	M	0.69635	19	0	0	0	0
2018/10/25	15:04:54	3720.3599	N	14055.6348	E	509.2	M	0.74838	19	0	0	0	0
2018/10/25	15:04:59	3720.3589	N	14055.6338	E	508.5	M	0.73396	19	0	0	0	0
2018/10/25	15:05:04	3720.3582	N	14055.6321	E	506.7	M	0.76706	19	0	0	0	0
2018/10/25	15:05:09	3720.3569	N	14055.631	E	506.9	M	0.67204	19	0	0	0	0
2018/10/25	15:05:14	3720.3555	N	14055.63	E	509.6	M	0.67609	19	0	0	0	0
2018/10/25	15:05:19	3720.3558	N	14055.6293	E	510.1	M	0.6815	19	0	0	0	0
2018/10/25	15:05:24	3720.3527	N	14055.6273	E	510.9	M	0.70442	19	0	0	0	0
2018/10/25	15:05:29	3720.3508	N	14055.6262	E	511.2	M	0.74224	19	0	0	0	0
2018/10/25	15:05:34	3720.349	N	14055.6242	E	511.7	M	0.68328	19	0	0	0	0
2018/10/25	15:05:39	3720.3486	N	14055.6233	E	511.8	M	0.64456	19	0	0	0	0
2018/10/25	15:05:44	3720.3476	N	14055.6211	E	512.8	M	0.6515	19	0	0	0	0
2018/10/25	15:05:49	3720.3453	N	14055.6198	E	513.2	M	0.70744	19	0	0	0	0
2018/10/25	15:05:54	3720.3428	N	14055.6187	E	513.1	M	0.63847	19	0	0	0	0
2018/10/25	15:59:09	3720.2117	N	14055.5538	E	504.3	M	0.65446	19	0	0	0	0
2018/10/25	15:59:14	3720.2105	N	14055.5558	E	505.5	M	0.58182	19	0	0	0	0
2018/10/25	15:59:19	3720.2096	N	14055.5581	E	506.7	M	0.56425	19	0	0	0	0
2018/10/25	15:59:24	3720.2086	N	14055.5601	E	507.2	M	0.48021	19	0	0	0	0
2018/10/25	15:59:29	3720.2063	N	14055.5614	E	509.3	M	0.47842	19	0	0	0	0
2018/10/25	15:59:34	3720.2047	N	14055.5628	E	510.9	M	0.43409	19	0	0	0	0
2018/10/25	15:59:40	3720.2026	N	14055.5643	E	512.1	M	0.39658	19	0	0	0	0
2018/10/25	15:59:44	3720.2009	N	14055.566	E	512.1	M	0.40609	19	0	0	0	0
2018/10/25	15:59:49	3720.1983	N	14055.5675	E	513.2	M	0.43378	19	0	0	0	0
2018/10/25	15:59:54	3720.1968	N	14055.5696	E	513.6	M	0.44698	19	0	0	0	0
2018/10/25	15:59:59	3720.1945	N	14055.5713	E	514.3	M	0.46354	19	0	0	0	0
2018/10/25	16:00:04	3720.1917	N	14055.5728	E	514.9	M	0.45911	19	0	0	0	0
2018/10/25	16:00:10	3720.1877	N	14055.5758	E	516.7	M	0.45778	19	0	0	0	0
2018/10/25	16:00:14	3720.184	N	14055.578	E	517.6	M	0.51374	19	0	0	0	0
2018/10/25	16:00:19	3720.1818	N	14055.5803	E	517.8	M	0.499	19	0	0	0	0
2018/10/25	16:00:24	3720.1794	N	14055.5836	E	519	M	0.46619	19	0	0	0	0
2018/10/25	16:00:29	3720.1762	N	14055.5862	E	519.8	M	0.42054	19	0	0	0	0
2018/10/25	16:00:34	3720.1739	N	14055.5869	E	518.3	M	0.32491	19	0	0	0	0
2018/10/25	16:00:39	3720.1721	N	14055.5892	E	515.6	M	0.23362	19	0	0	0	0

Table S1-2. Data sheet for October 10, 2019

Measuring points	<0.19	0.19-0.38	0.38-0.95	0.95-1.9	1.9-3.8	>3.8	μSv/h	Median	0.48	μSv/h
1110	0	138	940	32	0	0			4.2	mSv/y
	Ratio	Ratio	Ratio	Ratio	Ratio	Ratio		90th percentile	0.67	
	0.00	12.432	84.685	2.883	0.00	0.00	%	Average	0.51	
Detected ^{137}Cs	4.7	%						Min	0.19	
								Max	1.1	

Date	Time	Latitude		Longitude		Altitude		Dose rate (μSv/h)	Temperature	Detected ^{137}Cs	Levels 1-10	Detected ^{134}Cs	Levels 1-10
2019/10/10	13:39:32	3720.1628	N	14055.591	E	509.8	M	0.22315	19	0	0	0	0
2019/10/10	13:39:37	3720.1624	N	14055.5905	E	508.9	M	0.20226	19	0	0	0	0
[illegible]	[illegible]	[illegible]	N	[illegible]	E	509.2	M	0.23266	19	0	0	0	0
2019/10/10	14:08:09	3720.3488	N	14055.6475	E	519.9	M	0.92169	Ind.	137Cs	[illegible]	134Cs	1
2019/10/10	14:08:14	3720.3487	N	14055.6464	E	520.9	M	1.0589	Ind.	137Cs	4	134Cs	1
2019/10/10	14:08:19	3720.3498	N	14055.6445	E	519.5	M	0.89788	Ind.	137Cs	5	134Cs	1
2019/10/10	14:08:25	3720.3505	N	14055.6412	E	517.1	M	1.0816	Ind.	137Cs	5	134Cs	1
2019/10/10	14:08:30	3720.3508	N	14055.6401	E	513.1	M	1.065	Ind.	137Cs	6	134Cs	1
2019/10/10	14:08:34	3720.3517	N	14055.64	E	509	M	0.83087	Ind.	137Cs	6	134Cs	1
2019/10/10	14:08:39	3720.3527	N	14055.6401	E	507	M	0.76257	Ind.	137Cs	6	134Cs	1
2019/10/10	14:08:44	3720.3535	N	14055.6401	E	507.2	M	0.85443	Ind.	137Cs	6	134Cs	1
2019/10/10	14:08:49	3720.355	N	14055.6389	E	507.7	M	0.79645	Ind.	137Cs	6	134Cs	1
2019/10/10	14:08:54	3720.3577	N	14055.6348	E	504.6	M	0.71716	19	0	0	0	0
2019/10/10	14:08:59	3720.3594	N	14055.6334	E	506.5	M	0.70137	19	0	0	0	0
2019/10/10	14:09:04	3720.3594	N	14055.6321	E	507.8	M	0.7068	19	0	0	0	0
2019/10/10	14:09:09	3720.3583	N	14055.6317	E	509.8	M	0.64678	19	0	0	0	0
2019/10/10	14:09:14	3720.3573	N	14055.6317	E	511.4	M	0.67142	19	0	0	0	0
2019/10/10	14:09:19	3720.3565	N	14055.6313	E	512.2	M	0.66379	19	0	0	0	0
2019/10/10	14:09:24	3720.3565	N	14055.6315	E	514.8	M	0.62359	19	0	0	0	0
2019/10/10	14:09:29	3720.3559	N	14055.631	E	515.6	M	0.69014	19	0	0	0	0
2019/10/10	14:09:34	3720.3547	N	14055.6314	E	521.8	M	0.64114	19	0	0	0	0
2019/10/10	14:09:40	3720.3536	N	14055.6314	E	522.8	M	0.67866	19	0	0	0	0
2019/10/10	14:09:44	3720.3518	N	14055.6307	E	525.4	M	0.63262	19	0	0	0	0
2019/10/10	14:09:49	3720.3521	N	14055.6284	E	527.3	M	0.6266	19	0	0	0	0
2019/10/10	14:09:54	3720.3522	N	14055.6281	E	527.2	M	0.64969	19	0	0	0	0
2019/10/10	14:09:59	3720.3521	N	14055.628	E	527	M	0.69368	19	0	0	0	0
2019/10/10	14:10:04	3720.3521	N	14055.6277	E	527	M	0.70443	19	0	0	0	0
2019/10/10	14:10:09	3720.3501	N	14055.6269	E	524.2	M	0.64962	19	0	0	0	0
2019/10/10	14:10:14	3720.349	N	14055.6267	E	522.7	M	0.97861	19	0	0	0	0
2019/10/10	14:10:19	3720.3483	N	14055.6256	E	521.4	M	1.008	19	0	0	0	0
2019/10/10	14:10:24	3720.3481	N	14055.6221	E	520.4	M	0.97263	Ind.	137Cs	4	134Cs	1
2019/10/10	14:10:30	3720.3475	N	14055.6222	E	519.9	M	1.0105	Ind.	137Cs	5	134Cs	1
2019/10/10	14:10:35	3720.3474	N	14055.6222	E	519.9	M	0.99412	Ind.	137Cs	5	134Cs	1
2019/10/10	14:10:40	3720.3474	N	14055.6222	E	519.9	M	0.94098	Ind.	137Cs	6	134Cs	1
2019/10/10	14:10:45	3720.3474	N	14055.6221	E	519.9	M	0.97264	Ind.	137Cs	7	134Cs	1
2019/10/10	14:10:50	3720.3474	N	14055.6221	E	519.9	M	1.0363	Ind.	137Cs	7	134Cs	1
2019/10/10	14:10:56	3720.3474	N	14055.6221	E	519.9	M	1.0365	Ind.	137Cs	8	134Cs	1
2019/10/10	14:11:01	3720.3474	N	14055.6221	E	519.9	M	1.0196	Ind.	137Cs	8	134Cs	1
2019/10/10	14:11:06	3720.3474	N	14055.6221	E	519.9	M	1.0412	Ind.	137Cs	9	134Cs	1
2019/10/10	14:11:12	3720.3474	N	14055.6221	E	519.9	M	0.97848	Ind.	137Cs	9	134Cs	1
2019/10/10	14:11:17	3720.3474	N	14055.6221	E	519.9	M	0.99562	Ind.	137Cs	10	134Cs	2
2019/10/10	14:11:22	3720.3474	N	14055.6221	E	519.9	M	1.0411	Ind.	137Cs	10	134Cs	2
2019/10/10	14:11:27	3720.3474	N	14055.6221	E	519.9	M	1.0119	Ind.	137Cs	10	134Cs	2
2019/10/10	14:11:32	3720.3474	N	14055.6218	E	519.9	M	0.98848	Ind.	137Cs	10	134Cs	2
2019/10/10	14:11:37	3720.3474	N	14055.6218	E	519.9	M	1.0064	Ind.	137Cs	10	134Cs	2
2019/10/10	14:11:42	3720.3474	N	14055.6218	E	519.8	M	0.97902	Ind.	137Cs	10	134Cs	2
2019/10/10	14:11:47	3720.3473	N	14055.6215	E	519.8	M	1.0165	Ind.	137Cs	10	134Cs	2
2019/10/10	14:11:52	3720.3469	N	14055.6202	E	519.5	M	1.0021	Ind.	137Cs	10	134Cs	2
2019/10/10	14:11:57	3720.3464	N	14055.6213	E	519.5	M	0.95174	Ind.	137Cs	10	134Cs	2
2019/10/10	14:12:03	3720.3461	N	14055.6213	E	519.6	M	0.97359	Ind.	137Cs	10	134Cs	2
[illegible]	[illegible]	[illegible]	[illegible]	[illegible]	[illegible]	[illegible]	M	0.97563	Ind.	137Cs	10	134Cs	2
2019/10/10	15:11:35	3720.1864	N	14055.5732	E	531.7	M	[illegible]	[illegible]	[illegible]	[illegible]	[illegible]	[illegible]
2019/10/10	15:11:40	3720.183	N	14055.5751	E	533	M	0.52521	19	0	0	0	0
2019/10/10	15:11:44	3720.1807	N	14055.5782	E	533.8	M	0.51891	19	0	0	0	0
2019/10/10	15:11:50	3720.1788	N	14055.5798	E	534.9	M	0.54552	19	0	0	0	0
2019/10/10	15:11:56	3720.1768	N	14055.5818	E	535.9	M	0.46101	19	0	0	0	0
2019/10/10	15:12:01	3720.1724	N	14055.5845	E	538.3	M	0.3894	19	0	0	0	0
2019/10/10	15:12:06	3720.1692	N	14055.5856	E	539.6	M	0.28725	19	0	0	0	0

Table S1-3. Data sheet for October 17, 2019

Measuring points	<0.19	0.19-0.38	0.38-0.95	0.95-1.9	1.9-3.8	>3.8	μSv/h
880	3	173	703	1	0	0	
	Ratio	Ratio	Ratio	Ratio	Ratio	Ratio	
	0.34	19.66	79.89	0.11	0.00	0.00	%
Detected ^{137}Cs	1.4	%					

Median	0.45	μSv/h
	4.0	mSv/y
90th percentile	0.63	
Average	0.47	
Min	0.17	
Max	0.96	

Date	Time	Latitude		Longitude		Altitude		Dose rate (μSv/h)	Temperature	Detected ^{137}Cs	Levels 1-10	Detected ^{134}Cs	Levels 1-10
2019/10/17	14:56:47	3720.1633	N	14055.5773	E	517.9	M	0.1846	17	0	0	0	0
2019/10/17	14:56:52	3720.164	N	14055.5835	E	515.9	M	0.16684	17	0	0	0	0
2019/10/17	14:56:57	3720.1655	N	14055.5879	E	515.7	M	0.17011	17	0	0	0	0
2019/10/17	14:57:03	3720.1657	N	14055.5904	E	516.1	M	0.21687	17	0	0	0	0
2019/10/17	14:57:08	3720.1675	N	14055.5913	E	516	M	0.26379	17	0	0	0	0
2019/10/17	14:57:12	3720.1692	N	14055.5912	E	515.2	M	0.48307	17	0	0	0	0
2019/10/17	14:57:17	3720.1716	N	14055.5924	E	515.9	M	0.4312	17	0	0	0	0
2019/10/17	14:57:22	3720.1729	N	14055.5929	E	516.7	M	0.36604	17	0	0	0	0
2019/10/17	14:57:27	3720.1733	N	14055.5943	E	517.3	M	0.37763	17	0	0	0	0
2019/10/17	15:08:13	3720.304	N	14055.7097	E	586.6	M	0.64425	17	0	0	0	0
2019/10/17	15:08:19	3720.3044	N	14055.7111	E	586.5	M	0.71473	17	0	0	0	0
2019/10/17	15:08:24	3720.3051	N	14055.713	E	585.7	M	0.68483	Ind.	137Cs	4	134Cs	0
2019/10/17	15:08:29	3720.3059	N	14055.7144	E	585.9	M	0.69503	Ind.	137Cs	4	134Cs	0
2019/10/17	15:08:35	3720.3062	N	14055.716	E	586.1	M	0.68815	Ind.	137Cs	5	134Cs	0
2019/10/17	15:08:40	3720.3063	N	14055.7173	E	585.6	M	0.78507	Ind.	137Cs	5	134Cs	1
2019/10/17	15:08:44	3720.3068	N	14055.7199	E	584.8	M	0.69777	Ind.	137Cs	6	134Cs	1
2019/10/17	15:08:49	3720.3071	N	14055.7218	E	583.6	M	0.745	Ind.	137Cs	6	134Cs	1
2019/10/17	15:08:55	3720.3074	N	14055.7231	E	582.7	M	0.64831	Ind.	137Cs	7	134Cs	1
2019/10/17	15:09:00	3720.3071	N	14055.7248	E	582.5	M	0.62748	Ind.	137Cs	7	134Cs	1
2019/10/17	15:09:05	3720.3074	N	14055.7258	E	581.1	M	0.63223	17	0	0	0	0
2019/10/17	15:09:10	3720.3077	N	14055.7272	E	580.3	M	0.56552	17	0	0	0	0
2019/10/17	15:09:15	3720.3079	N	14055.728	E	579.5	M	0.58039	Ind.	137Cs	8	134Cs	1
2019/10/17	15:09:19	3720.3089	N	14055.7298	E	577.5	M	0.57116	17	0	0	0	0
2019/10/17	15:19:25	3720.3554	N	14055.6533	E	514.1	M	0.64177	17	0	0	0	0
2019/10/17	15:19:29	3720.3544	N	14055.653	E	515.2	M	0.82881	17	0	0	0	0
2019/10/17	15:19:34	3720.3524	N	14055.6533	E	515.3	M	0.71496	17	0	0	0	0
2019/10/17	15:19:39	3720.3504	N	14055.6525	E	516	M	0.75869	17	0	0	0	0
2019/10/17	15:19:45	3720.3507	N	14055.6511	E	515.8	M	0.84742	17	0	0	0	0
2019/10/17	15:19:50	3720.3507	N	14055.6502	E	515.6	M	0.82704	17	0	0	0	0
2019/10/17	15:19:56	3720.3508	N	14055.649	E	515.6	M	0.95617	Ind.	137Cs	4	17	0
2019/10/17	15:20:01	3720.3503	N	14055.6467	E	515.1	M	0.90559	Ind.	137Cs	5	134Cs	0
2019/10/17	15:20:06	3720.3503	N	14055.6461	E	514.2	M	0.74828	17	0	0	0	0
2019/10/17	15:20:11	3720.3505	N	14055.6438	E	514.2	M	0.73261	Ind.	137Cs	5	134Cs	0
2019/10/17	15:20:15	3720.3524	N	14055.6416	E	515.3	M	0.69701	17	0	0	0	0
2019/10/17	15:20:21	3720.3541	N	14055.6394	E	515.9	M	0.65427	17	0	0	0	0
2019/10/17	15:20:26	3720.3551	N	14055.6367	E	515.7	M	0.59556	17	0	0	0	0
2019/10/17	15:20:30	3720.3562	N	14055.6341	E	514.7	M	0.71817	17	0	0	0	0
2019/10/17	15:20:35	3720.3559	N	14055.6329	E	514.1	M	0.68571	17	0	0	0	0
2019/10/17	15:20:40	3720.3561	N	14055.6321	E	513.5	M	0.60218	17	0	0	0	0
2019/10/17	15:20:45	3720.3555	N	14055.6311	E	512.8	M	0.64595	17	0	0	0	0
2019/10/17	15:20:50	3720.3542	N	14055.6307	E	511.7	M	0.64069	17	0	0	0	0
2019/10/17	15:20:55	3720.3514	N	14055.6293	E	511.1	M	0.64198	17	0	0	0	0
2019/10/17	16:09:05	3720.2097	N	14055.5548	E	512.3	M	0.45686	17	0	0	0	0
2019/10/17	16:09:10	3720.2074	N	14055.5586	E	512.9	M	0.42543	17	0	0	0	0
2019/10/17	16:09:15	3720.2054	N	14055.5621	E	514.6	M	0.39804	17	0	0	0	0
2019/10/17	16:09:20	3720.2024	N	14055.5651	E	515.5	M	0.40491	17	0	0	0	0
2019/10/17	16:09:25	3720.1992	N	14055.5682	E	515.5	M	0.3831	17	0	0	0	0
2019/10/17	16:09:30	3720.1964	N	14055.572	E	515.9	M	0.42061	17	0	0	0	0
2019/10/17	16:09:35	3720.1944	N	14055.5746	E	516.5	M	0.40657	17	0	0	0	0
2019/10/17	16:09:40	3720.1909	N	14055.5772	E	518.2	M	0.3818	17	0	0	0	0
2019/10/17	16:09:45	3720.1883	N	14055.5785	E	518.1	M	0.46959	17	0	0	0	0
2019/10/17	16:09:50	3720.1853	N	14055.5806	E	518.6	M	0.42295	17	0	0	0	0
2019/10/17	16:09:55	3720.1827	N	14055.5823	E	520.6	M	0.42446	17	0	0	0	0
2019/10/17	16:10:00	3720.1786	N	14055.5856	E	521.2	M	0.41959	17	0	0	0	0
2019/10/17	16:10:05	3720.1751	N	14055.5871	E	521.6	M	0.3283	17	0	0	0	0
2019/10/17	16:10:10	3720.171	N	14055.589	E	520.6	M	0.28062	17	0	0	0	0

Table S1-4. Data sheet for October 24, 2019

Measuring points	<0.19	0.19-0.38	0.38-0.95	0.95-1.9	1.9-3.8	>3.8	
1037	0	182	851	4	0	0	μ Sv/h
	Ratio	Ratio	Ratio	Ratio	Ratio	Ratio	
	0.00	17.55	82.06	0.39	0.00	0.00	%
Detected ^{137}Cs	1.1	%					

Median	0.45	μ Sv/h
	4.0	mSv/y
90th percentile	0.60	
Average	0.47	
Min	0.20	
Max	1.1	

Date	Time	Latitude		Longitude		Altitude		Dose rate (μ Sv/h)	Temperature	Detected ^{137}Cs	Levels 1-10	Detected ^{134}Cs	Levels 1-10
2019/10/24	13:07:49	3720.1649	N	14055.5862	E	517.3	M	0.19594	19	0	0	0	0
2019/10/24	13:07:54	3720.1651	N	14055.5868	E	517.3	M	0.19823	19	0	0	0	0
2019/10/24	13:07:59	3720.1651	N	14055.5879	E	517.5	M	0.23186	19	0	0	0	0
2019/10/24	13:08:04	3720.1662	N	14055.5921	E	518.2	M	0.24737	19	0	0	0	0
2019/10/24	13:08:09	3720.1653	N	14055.5922	E	518.7	M	0.30735	19	0	0	0	0
2019/10/24	13:08:14	3720.1655	N	14055.5927	E	518.4	M	0.29788	19	0	0	0	0
2019/10/24	13:08:19	3720.1673	N	14055.5927	E	519.2	M	0.36968	19	0	0	0	0
2019/10/24	13:08:24	3720.17	N	14055.5932	E	519.8	M	0.4321	19	0	0	0	0
2019/10/24	13:08:29	3720.1723	N	14055.5938	E	520.4	M	0.39588	19	0	0	0	0
2019/10/24	13:08:34	3720.1744	N	14055.5947	E	522.1	M	0.36871	19	0	0	0	0
[illegible]	[illegible]	[illegible]	[illegible]	[illegible]	E	[illegible]	M	[illegible]	19	0	0	0	0
2019/10/24	13:25:05	3720.3029	N	14055.7155	E	579.3	M	0.72032	[illegible]	[illegible]	[illegible]	[illegible]	0
2019/10/24	13:25:11	3720.3029	N	14055.7166	E	579.7	M	0.76214	Ind.	137Cs	4	19	0
2019/10/24	13:25:16	3720.3029	N	14055.7183	E	579.1	M	0.85746	Ind.	137Cs	5	19	0
2019/10/24	13:25:21	3720.3031	N	14055.7198	E	579	M	0.84164	Ind.	137Cs	5	134Cs	0
2019/10/24	13:25:26	3720.3033	N	14055.7219	E	578.7	M	0.77281	Ind.	137Cs	6	134Cs	1
2019/10/24	13:25:31	3720.3041	N	14055.724	E	577.9	M	0.62431	Ind.	137Cs	6	134Cs	1
2019/10/24	13:25:36	3720.305	N	14055.7253	E	577.1	M	0.72873	Ind.	137Cs	7	134Cs	1
2019/10/24	13:25:41	3720.3053	N	14055.7267	E	576.1	M	0.60235	-999	0	0	0	0
2019/10/24	13:25:46	3720.306	N	14055.7278	E	573.9	M	0.59716	Ind.	137Cs	7	134Cs	1
2019/10/24	13:25:51	3720.3064	N	14055.7295	E	572.8	M	0.55863	19	0	0	0	0
[illegible]	[illegible]	[illegible]	[illegible]	[illegible]	E	[illegible]	M	[illegible]	19	0	0	0	0
2019/10/24	13:39:02	3720.3535	N	14055.6505	E	513.9	M	0.96003	[illegible]	[illegible]	[illegible]	[illegible]	0
2019/10/24	13:39:08	3720.3542	N	14055.6514	E	512.6	M	1.0298	Ind.	137Cs	4	-999	0
2019/10/24	13:39:13	3720.3565	N	14055.6504	E	511.4	M	0.86932	Ind.	137Cs	5	134Cs	0
2019/10/24	13:39:17	3720.3565	N	14055.6506	E	512.1	M	1.0247	19	0	0	0	0
2019/10/24	13:39:23	3720.3573	N	14055.6499	E	511.6	M	1.0645	Ind.	137Cs	6	134Cs	1
2019/10/24	13:39:28	3720.359	N	14055.6469	E	513	M	0.76722	Ind.	137Cs	6	134Cs	1
2019/10/24	13:39:33	3720.36	N	14055.6458	E	512.7	M	0.7173	19	0	0	0	0
2019/10/24	13:39:38	3720.3611	N	14055.6439	E	513	M	0.78364	19	0	0	0	0
2019/10/24	13:39:43	3720.3619	N	14055.6417	E	512.4	M	0.70618	19	0	0	0	0
2019/10/24	13:39:48	3720.3624	N	14055.6388	E	511.3	M	0.6498	19	0	0	0	0
2019/10/24	13:39:53	3720.3615	N	14055.6355	E	508.2	M	0.73285	19	0	0	0	0
2019/10/24	13:39:58	3720.3614	N	14055.6313	E	506.7	M	0.6591	19	0	0	0	0
2019/10/24	13:40:02	3720.3624	N	14055.628	E	507.1	M	0.66811	19	0	0	0	0
2019/10/24	13:40:08	3720.3617	N	14055.6271	E	508.1	M	0.63051	19	0	0	0	0
2019/10/24	13:40:13	3720.3606	N	14055.6259	E	508.9	M	0.63283	19	0	0	0	0
2019/10/24	13:40:18	3720.359	N	14055.6244	E	509.2	M	0.6237	19	0	0	0	0
2019/10/24	13:40:23	3720.3584	N	14055.6216	E	510	M	0.69618	19	0	0	0	0
2019/10/24	13:40:28	3720.3568	N	14055.6197	E	511.4	M	0.68709	19	0	0	0	0
2019/10/24	14:32:57	3720.2086	N	14055.5382	E	499.3	M	0.64091	18.9659996	0	0	0	0
2019/10/24	14:33:01	3720.2068	N	14055.542	E	499.6	M	0.55684	18.70899963	0	0	0	0
2019/10/24	14:33:06	3720.2065	N	14055.5468	E	501	M	0.53663	18.82799911	0	0	0	0
2019/10/24	14:33:12	3720.2062	N	14055.5506	E	502	M	0.50102	18.89800072	0	0	0	0
2019/10/24	14:33:17	3720.2052	N	14055.5548	E	503.4	M	0.46665	18.79299927	0	0	0	0
2019/10/24	14:33:22	3720.2051	N	14055.5574	E	504.9	M	0.44253	18.87800026	0	0	0	0
2019/10/24	14:33:27	3720.2039	N	14055.5597	E	505.9	M	0.41797	18.82799911	0	0	0	0
2019/10/24	14:33:31	3720.2043	N	14055.5623	E	507	M	0.4205	18.89800072	0	0	0	0
2019/10/24	14:33:37	3720.203	N	14055.5656	E	507.4	M	0.42052	18.85899925	0	0	0	0
2019/10/24	14:33:42	3720.2012	N	14055.5683	E	508	M	0.43828	18.84399986	0	0	0	0
2019/10/24	14:33:47	3720.2	N	14055.5704	E	508.1	M	0.38951	18.72699928	0	0	0	0
2019/10/24	14:33:52	3720.1976	N	14055.5735	E	508.5	M	0.4251	18.75799942	0	0	0	0
2019/10/24	14:33:57	3720.1938	N	14055.5784	E	509.3	M	0.46452	18.7670002	0	0	0	0
2019/10/24	14:34:02	3720.1911	N	14055.5818	E	509.3	M	0.4333	18.86199951	0	0	0	0
2019/10/24	14:34:07	3720.1881	N	14055.5855	E	508.6	M	0.41956	18.91900063	0	0	0	0
2019/10/24	14:34:12	3720.1817	N	14055.5901	E	508.3	M	0.31361	18.95199966	0	0	0	0
2019/10/24	14:34:17	3720.1777	N	14055.5936	E	509	M	0.23946	18.90600014	0	0	0	0

Table S1-5. Data sheet for December 5, 2019

Measuring points	<0.19	0.19-0.38	0.38-0.95	0.95-1.9	1.9-3.8	>3.8		Median	0.47	μSv/h
1105	0	183	918	4	0	0	μSv/h		4.1	mSv/y
	Ratio	Ratio	Ratio	Ratio	Ratio	Ratio		90th percentile	0.64	
	0.00	16.56	83.08	0.36	0.00	0.00	%	Average	0.49	
Detected ^{137}Cs	2.4	%						Min	0.19	
								Max	1.1	

Date	Time	Latitude		Longitude		Altitude		Dose rate (μSv/h)	Temperature	Detected ^{137}Cs	Levels 1-10	Detected ^{134}Cs	Levels 1-10
2019/12/5	10:09:06	3720.1635	N	14055.5911	E	513.6	M	0.1923	13	0	0	0	0
2019/12/5	10:09:11	3720.1623	N	14055.5913	E	512.6	M	0.21136	13	0	0	0	0
2019/12/5	10:09:17	3720.1621	N	14055.5916	E	513.8	M	0.24232	13	0	0	0	0
2019/12/5	10:09:21	3720.1623	N	14055.5905	E	515.5	M	0.26836	13	0	0	0	0
2019/12/5	10:09:26	3720.1633	N	14055.5895	E	518.3	M	0.42296	13	0	0	0	0
2019/12/5	10:09:31	3720.165	N	14055.5896	E	518.9	M	0.46409	13	0	0	0	0
2019/12/5	10:09:36	3720.167	N	14055.5899	E	519.3	M	0.3993	13	0	0	0	0
2019/12/5	10:09:42	3720.1707	N	14055.5926	E	519.9	M	0.354	13	0	0	0	0
2019/12/5	10:09:47	3720.1727	N	14055.5942	E	519.5	M	0.36963	13	0	0	0	0
2019/12/5	10:09:52	3720.175	N	14055.5965	E	519.6	M	0.38239	13	0	0	0	0
2019/12/5	10:09:57	3720.1757	N	14055.5976	E	520.8	M	0.38906	13	0	0	0	0
2019/12/5	10:10:01	3720.1757	N	14055.5987	E	520.5	M	0.39637	13	0	0	0	0
2019/12/5	10:10:06	3720.1752	N	14055.6008	E	520.9	M	0.41807	13	0	0	0	0
2019/12/5	10:10:11	3720.176	N	14055.603	E	522	M	0.45006	13	0	0	0	0
2019/12/5	10:10:16	3720.1758	N	14055.6034	E	523.8	M	0.43958	13	0	0	0	0
2019/12/5	10:10:21	3720.1752	N	14055.6044	E	524.9	M	0.47531	13	0	0	0	0
2019/12/5	10:10:26	3720.1755	N	14055.607	E	525	M	0.44176	13	0	0	0	0
2019/12/5	10:10:32	3720.1754	N	14055.6096	E	525.3	M	0.44514	13	0	0	0	0
2019/12/5	10:10:37	3720.1751	N	14055.6136	E	524.4	M	0.48537	Ind.	137Cs	3	0	0
2019/12/5	10:10:41	3720.1729	N	14055.6178	E	525.2	M	0.50308	Ind.	137Cs	4	0	0
2019/12/5	10:10:47	3720.1725	N	14055.6217	E	525.8	M	0.46643	Ind.	137Cs	4	0	0
2019/12/5	10:10:52	3720.1734	N	14055.6261	E	526	M	0.53157	Ind.	137Cs	4	134Cs	0
2019/12/5	10:10:56	3720.1751	N	14055.6301	E	527.6	M	0.49133	Ind.	137Cs	5	134Cs	0
2019/12/5	10:11:01	3720.1765	N	14055.6334	E	527.8	M	0.53425	Ind.	137Cs	5	134Cs	0
2019/12/5	10:11:06	3720.1775	N	14055.6377	E	529	M	0.56601	Ind.	137Cs	5	134Cs	0
2019/12/5	10:11:11	3720.1789	N	14055.6427	E	532.6	M	0.59465	Ind.	137Cs	6	134Cs	1
2019/12/5	10:11:16	3720.1778	N	14055.6452	E	533.3	M	0.57037	Ind.	137Cs	6	134Cs	1
2019/12/5	10:11:22	3720.1765	N	14055.6471	E	534	M	0.51028	Ind.	137Cs	6	134Cs	1
2019/12/5	10:11:27	3720.1756	N	14055.6498	E	533.8	M	0.42988	Ind.	137Cs	6	134Cs	1
2019/12/5	10:11:32	3720.1753	N	14055.6523	E	534.5	M	0.39167	Ind.	137Cs	7	134Cs	1
2019/12/5	10:11:36	3720.1736	N	14055.6549	E	536.3	M	0.40269	13	0	0	0	0
2019/12/5	10:11:42	3720.172	N	14055.6595	E	537.9	M	0.43731	Ind.	137Cs	7	134Cs	1
2019/12/5	10:11:47	3720.1719	N	14055.6615	E	538.7	M	0.40737	13	0	0	0	0
2019/12/5	10:36:24	3720.3557	N	[illegible]	E	[illegible]	M	[illegible]	[illegible]	[illegible]	[illegible]	[illegible]	[illegible]
2019/12/5	10:36:29	3720.3562	N	14055.6393	E	517.7	M	1.1496	Ind.	137Cs	5	134Cs	1
2019/12/5	10:36:34	3720.3571	N	14055.638	E	517.4	M	0.80914	Ind.	137Cs	6	134Cs	1
2019/12/5	10:36:39	3720.3578	N	14055.6374	E	516.6	M	0.8601	Ind.	137Cs	6	134Cs	1
2019/12/5	10:36:44	3720.3589	N	14055.6356	E	515.5	M	0.80902	Ind.	137Cs	6	134Cs	1
2019/12/5	10:36:49	3720.3597	N	14055.6335	E	511.8	M	0.76397	Ind.	137Cs	7	134Cs	1
2019/12/5	10:36:54	3720.361	N	14055.6318	E	511.5	M	0.69747	11	0	0	0	0
2019/12/5	11:39:54	3720.2151	N	14055.5695	E	510.1	M	0.48189	11	[illegible]	[illegible]	[illegible]	0
2019/12/5	11:40:00	3720.2131	N	14055.5711	E	512.2	M	0.43058	11	0	0	0	0
2019/12/5	11:40:04	3720.2084	N	14055.5725	E	512.8	M	0.39818	11	0	0	0	0
2019/12/5	11:40:09	3720.205	N	14055.5739	E	513.2	M	0.36117	10	0	0	0	0
2019/12/5	11:40:14	3720.2025	N	14055.5761	E	513.1	M	0.38184	-999	0	0	0	0
2019/12/5	11:40:19	3720.1993	N	14055.578	E	512.2	M	0.43484	10	0	0	0	0
2019/12/5	11:40:24	3720.1965	N	14055.5794	E	512.1	M	0.40829	10	0	0	0	0
2019/12/5	11:40:30	3720.1959	N	14055.5821	E	512.3	M	0.42107	10	0	0	0	0
2019/12/5	11:40:36	3720.1929	N	14055.5843	E	512.9	M	0.45401	10	0	0	0	0
2019/12/5	11:40:40	3720.1903	N	14055.5852	E	513.8	M	0.46663	10	0	0	0	0
2019/12/5	11:40:45	3720.1881	N	14055.5864	E	513.7	M	0.48469	11	0	0	0	0
2019/12/5	11:40:50	3720.1849	N	14055.5887	E	514	M	0.49821	10	0	0	0	0
2019/12/5	11:40:55	3720.1818	N	14055.5908	E	515.1	M	0.49452	10	0	0	0	0
2019/12/5	11:41:00	3720.1798	N	14055.5922	E	515.1	M	0.44411	11	0	0	0	0
2019/12/5	11:41:05	3720.1771	N	14055.5929	E	515.4	M	0.34879	11	0	0	0	0
2019/12/5	11:41:10	3720.1742	N	14055.5922	E	514.8	M	0.30072	11	0	0	0	0
2019/12/5	11:41:15	3720.173	N	14055.5917	E	514.3	M	0.19707	11	0	0	0	0

Fig S1-1. Radiation map for October 10, 2018

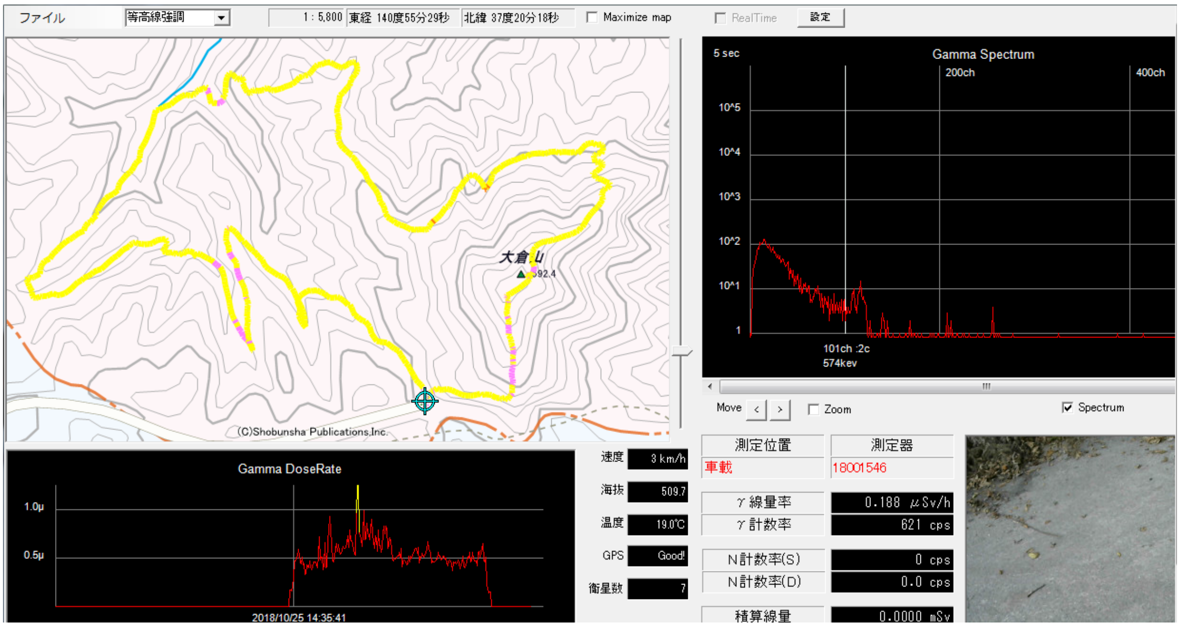

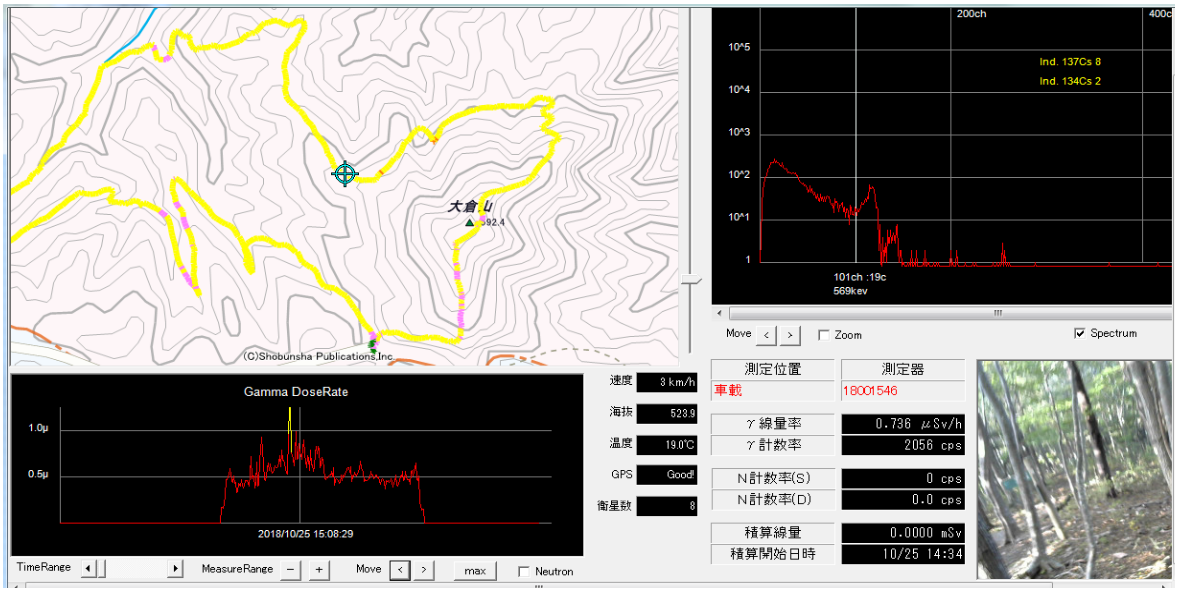

Fig S1-2. Radiation map for October 10, 2019

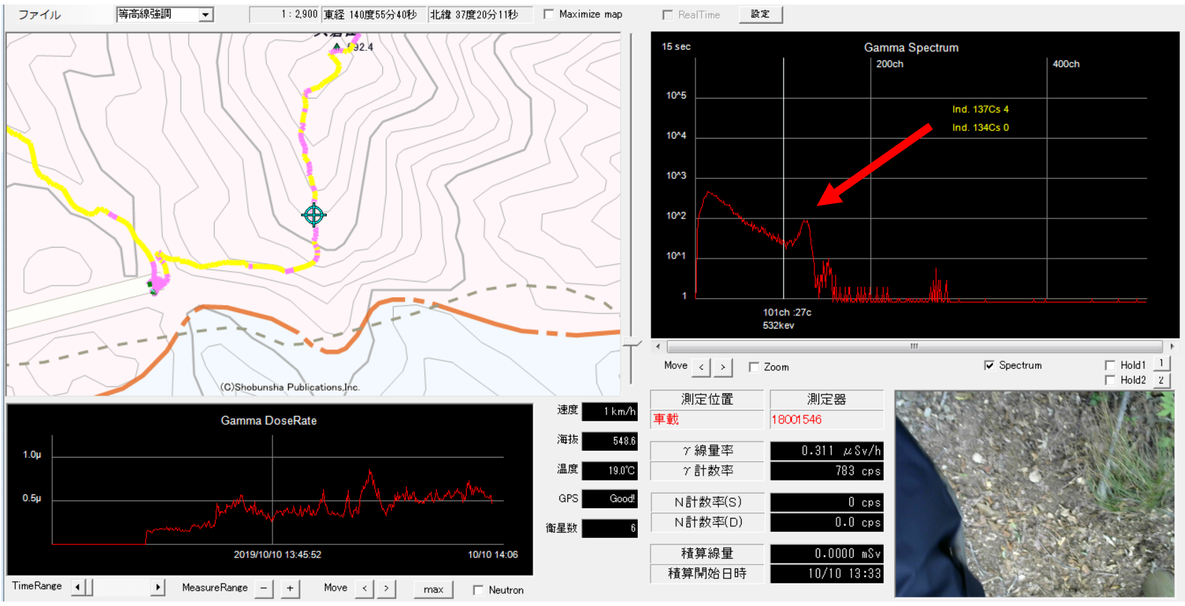

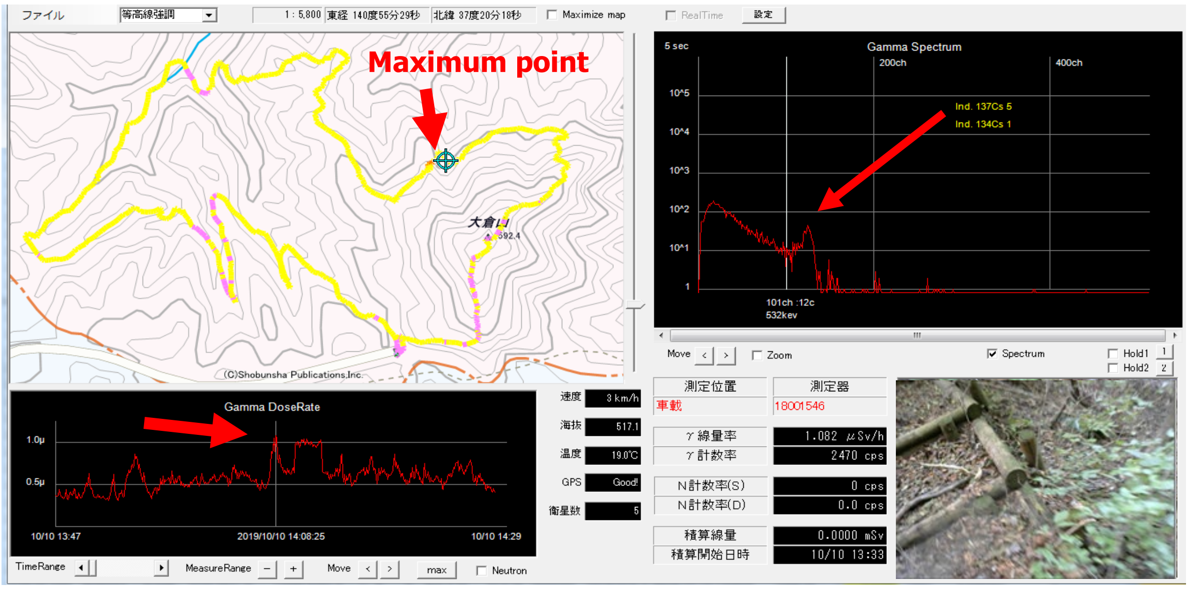

Fig S1-3. Radiation map for October 17, 2019

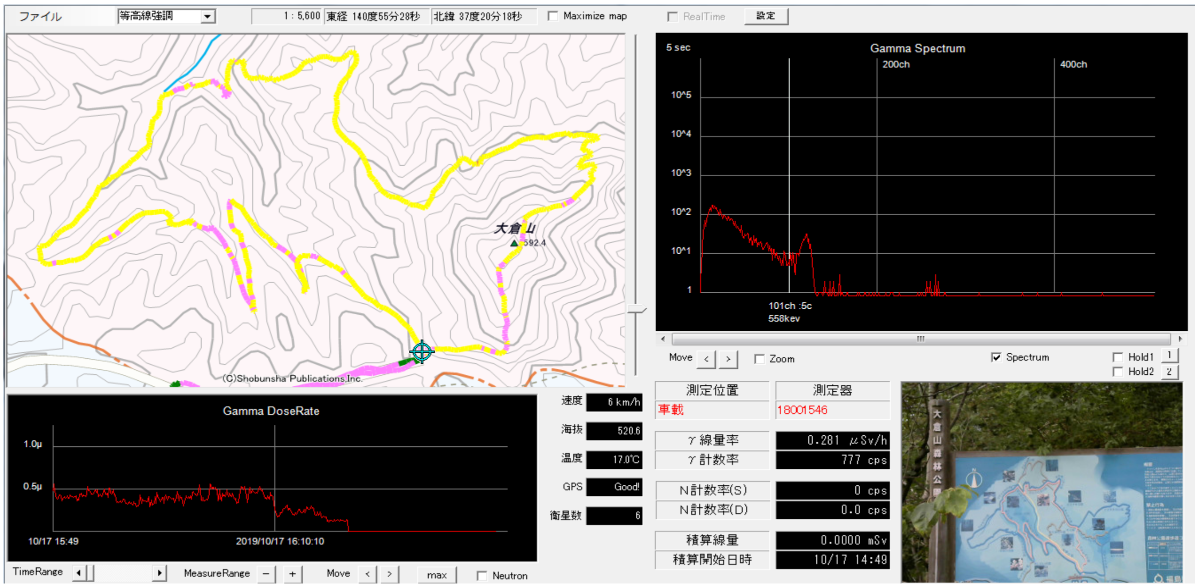

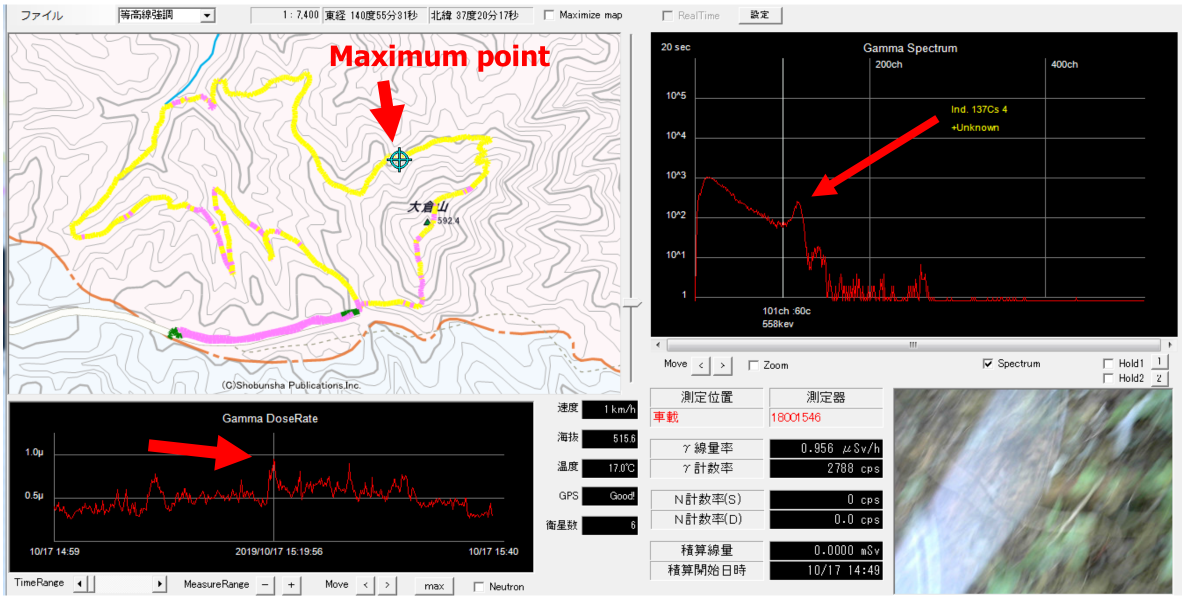

Fig S1-4. Radiation map for October 24, 2019

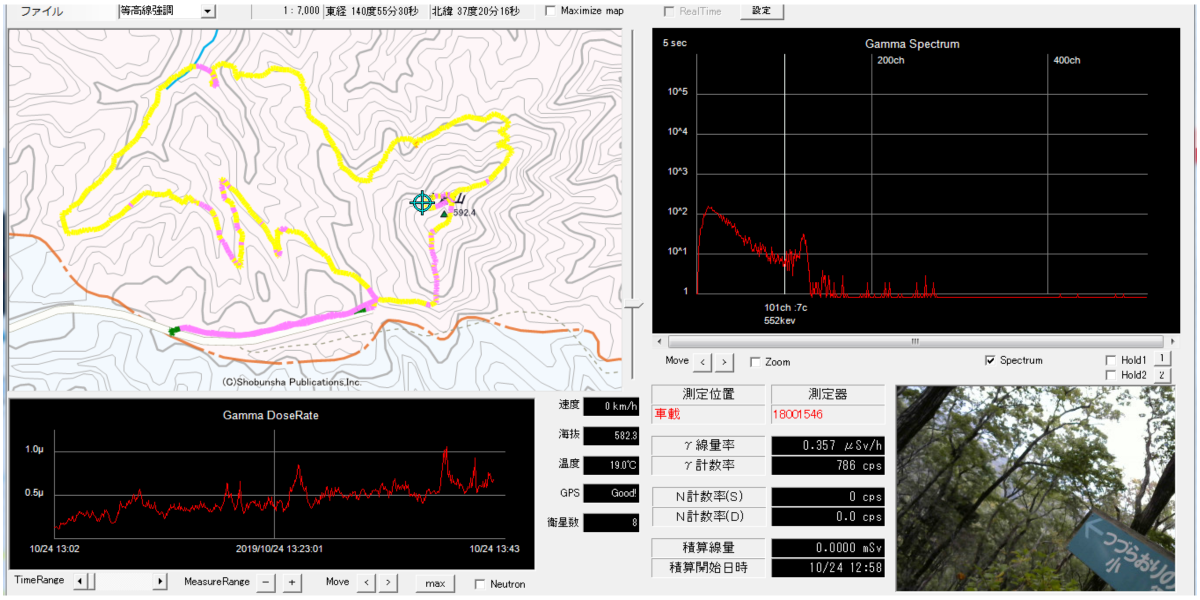

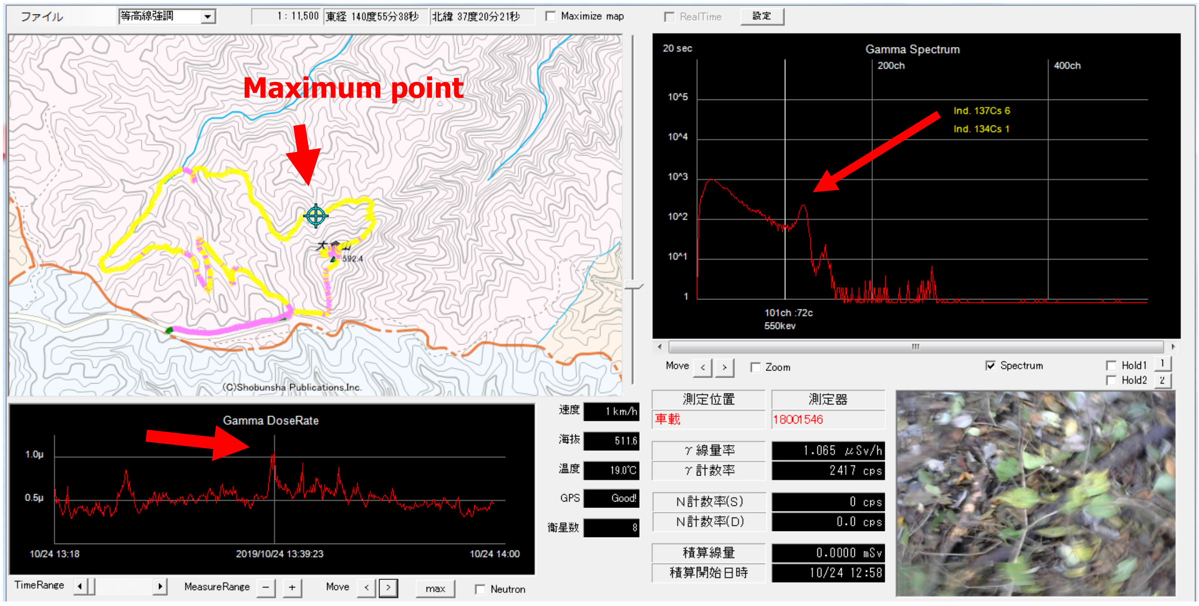

Fig S1-5. Radiation map for December 5, 2019

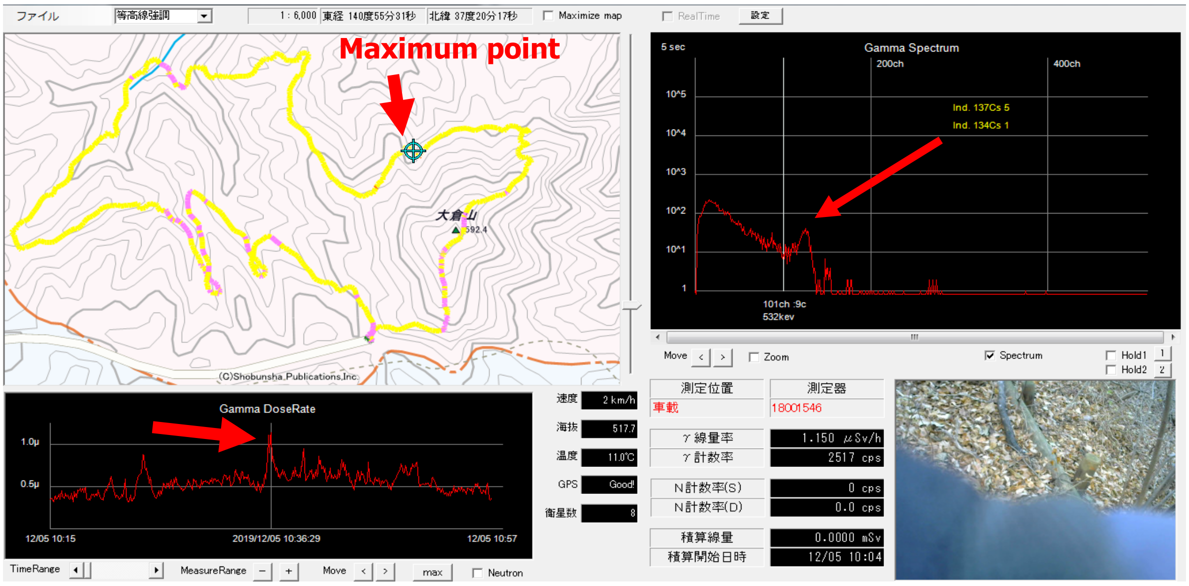

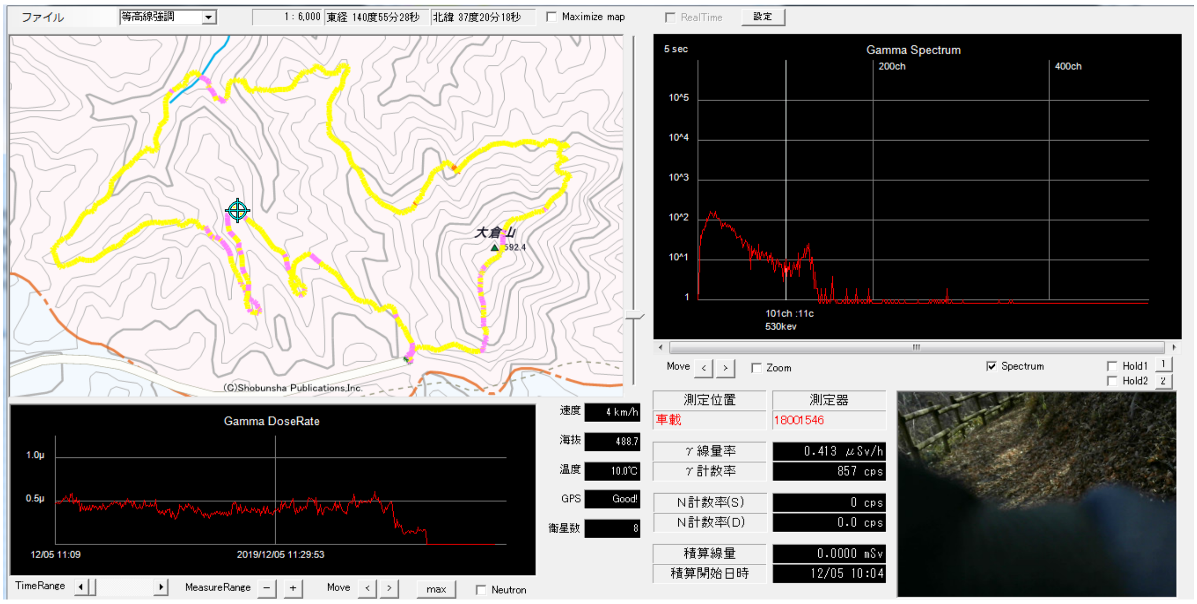

Fig S1-6. Chronological changes in ambient dose rates along the Mt Okura hiking trail

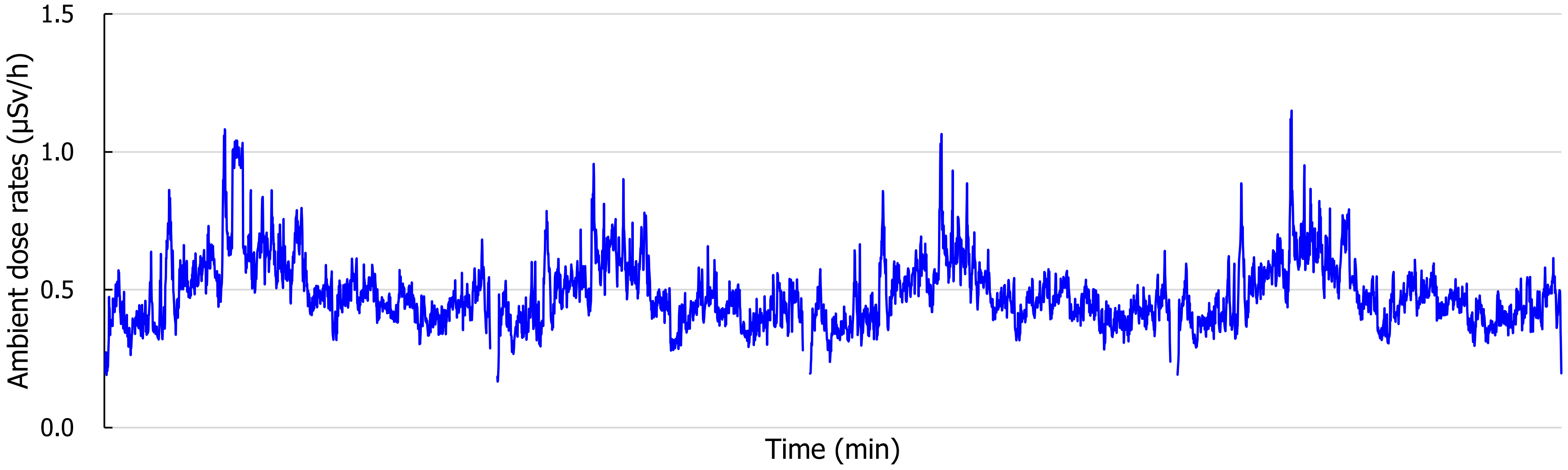

Fig S1-6 shows the variation in ambient dose rates along the Mt Okura hiking trail based on data collected by walking survey conducted using a Radi-probe system in October and December 2019. This figure is the same as Fig 3 (A).

Fig S1-7. Variation in ambient dose rates along the Mt Okura hiking trail

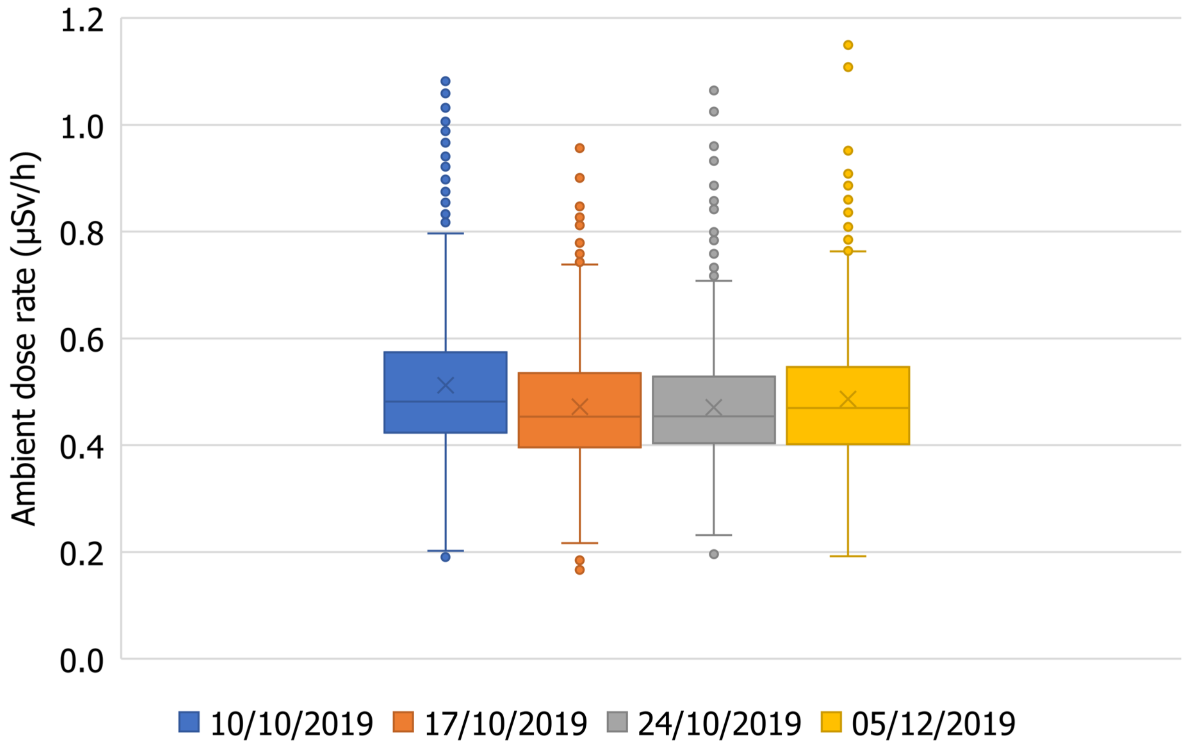

Fig S1-7 shows box plots of the variation in ambient dose rates based on data collected by walking survey conducted using a Radi-probe system in October and December 2019.

Fig S1-8. Chronological changes of ambient dose rates along the Mt Okura hiking trail

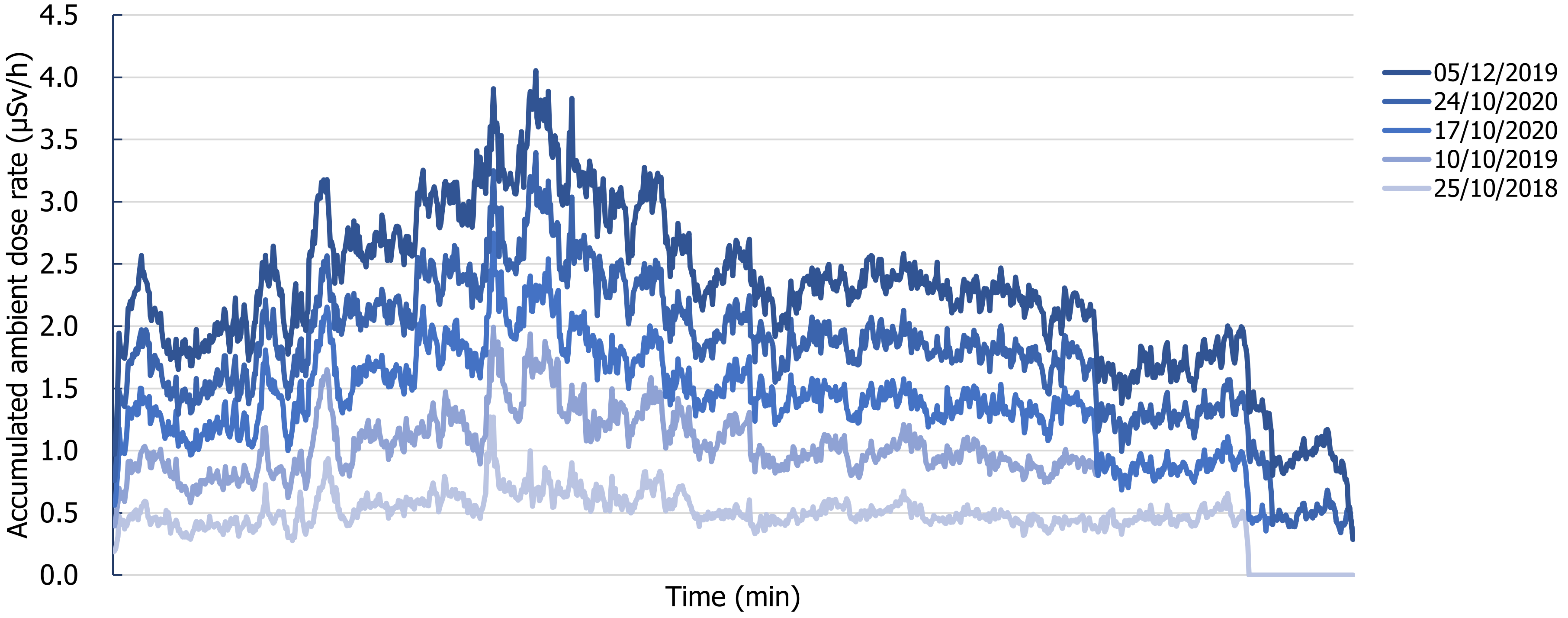

Fig S1-8 shows a stacked line graph of accumulated ambient dose rates based on the data collected by walking survey conducted using a Radi-probe system in October and December 2019.

Fig S1-9. Chronological changes in ambient dose rates along the Mt Okura hiking trail

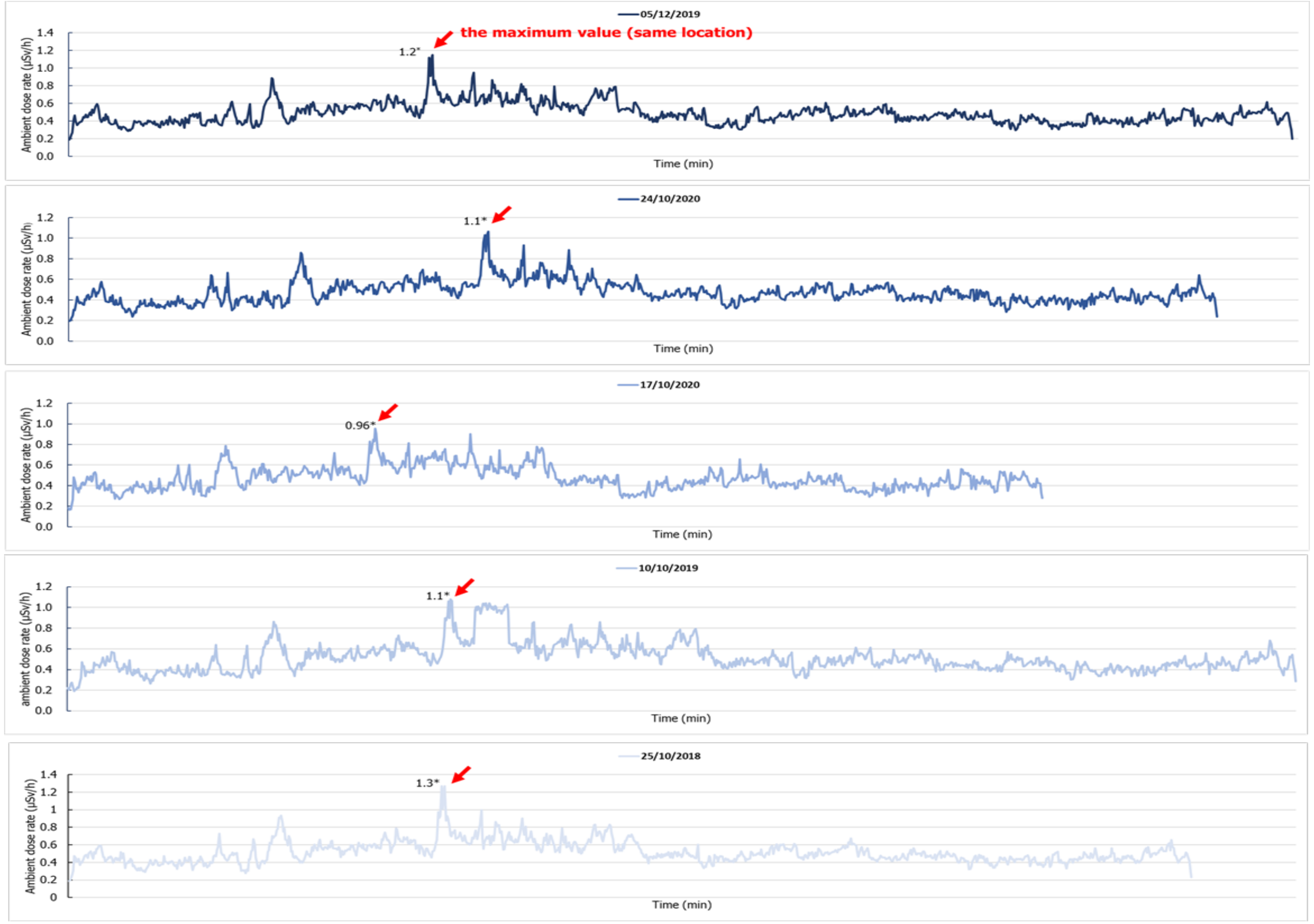

Fig S1-9 shows a stacked line graph of ambient dose rates based on data collected by walking survey conducted using a Radi-probe system in October and December 2019. Single asterisks show the maximum μSv/h values obtained using the Radi-probe. Red arrows show the same location on the hiking trail.

Table S2-1 Estimated reduction of the ambient dose rates within the Mt Okura hiking trail

No.	Release date	survey date	Days after the release	Years after release	The radioactivty of Cs-134 (PBq)	The radioactivty of Cs-137 (PBq)	Ambient dose by Cs-134 ($\times 10^9$ Sv/h)	Ambient dose by Cs-137 ($\times 10^9$ Sv/h)	Total ($\times 10^9$ Sv/h)
1	2011/3/14	2018/10/25	2782	7.6	0.70	7.39	5.9	23.3	29.2
2	2011/3/14	2019/10/10	3132	8.6	0.51	7.23	4.3	22.8	27.1
3	2011/3/14	2019/10/17	3139	8.6	0.50	7.22	4.3	22.8	27.0
4	2011/3/14	2019/10/24	3146	8.6	0.50	7.22	4.2	22.7	27.0
5	2011/3/14	2019/12/5	3188	8.7	0.48	7.20	4.1	22.7	26.8
						physical decay (No.1-5)	31.2%	2.5%	8.3%
						physical decay (No.2-5)	**5.0%**	**0.35%**	**1.1%**

^{134}Cs (2.065 years of half-life) and ^{137}Cs (30.17 years of half-life) were equal 9.0, and 8.8 PBq at release time.

Ambient dose equivalent conversion coefficients for radionuclides exponentially distributed in the ground: Cs-134: 8.5 μSv/h per Bq/m^2; Cs-137: 3.15 μSv/h per Bq/m^2.[1]

1. Saito K, Petoussi-Henss N. 2014. Ambient dose equivalent conversion coefficients for radionuclides exponentially distributed in the ground. *Journal of Nuclear Science and Technology* 51:1274–1287. DOI: 10.1080/00223131.2014.919885.

Table S3-1. Data sheet for October 25, 2018

RadEye SPRD History	Version 1.42.0	30												
Date (extracted data)	Serial number	Version	Type	Unit GUnit N	Timer interval	Flag 1	Flag 2	Flag 3	Flag 4	Flag Unit G	Flag Unit N	Flag Type	Index Unit	HistCalMode
25/10/2018	10110	RadEye SPRD E1.16	Sv/h	cps	60	64	183	0	2056	261	0	48	5	0

Survey	
Average（μSv/h)	0.54
Median（μSv/h)	0.51
90th percentile	0.72
Min	0.18
Max	1.01
Estimated annual dose rate（mSv/y)	4.4

Date	Mean G	Unit	Max G/Bgnd G	Unit	Mean DR-G[μSv/h]	Unit	Max DR[μSv/h]/nc	Unit	N[cps]	Unit	Max N/Bgnd N[cps]	Unit	M. time	Temperature	#	Status	Date [dd.mm.yy]	Time [hh:mm]
43398.60882	7.33E+01	cps	8.23E+01	cps	1.77E-01	μSv/h	2.46E-01	μSv/h	8.33E-02	cps	7.49E-02	cps	60	19	0	21760	25.10.18	14:36
43398.60951	9.48E+01	cps	1.55E+02	cps	2.08E-01	μSv/h	2.78E-01	μSv/h	1.00E-01	cps	1.26E-01	cps	60	19	0	21760	25.10.18	14:37
43398.6102	1.66E+02	cps	1.87E+02	cps	4.83E-01	μSv/h	5.65E-01	μSv/h	1.00E-01	cps	1.07E-01	cps	60	19	0	21760	25.10.18	14:38
43398.61087	1.92E+02	cps	2.10E+02	cps	5.38E-01	μSv/h	5.63E-01	μSv/h	1.17E-01	cps	1.50E-01	cps	60	19	0	21760	25.10.18	14:39
43398.6116	1.78E+02	cps	2.14E+02	cps	5.25E-01	μSv/h	5.75E-01	μSv/h	1.00E-01	cps	1.22E-01	cps	60	20	0	21760	25.10.18	14:40
43398.61229	1.71E+02	cps	1.97E+02	cps	4.69E-01	μSv/h	5.59E-01	μSv/h	8.33E-02	cps	1.50E-01	cps	60	20	0	21760	25.10.18	14:41
43398.61299	1.50E+02	cps	1.70E+02	cps	4.16E-01	μSv/h	4.92E-01	μSv/h	3.33E-02	cps	1.14E-01	cps	60	20	0	21760	25.10.18	14:42
43398.61368	1.41E+02	cps	1.84E+02	cps	3.62E-01	μSv/h	4.99E-01	μSv/h	6.67E-02	cps	9.72E-02	cps	60	20	0	21760	25.10.18	14:43
43398.61438	1.58E+02	cps	1.81E+02	cps	4.35E-01	μSv/h	5.19E-01	μSv/h	1.17E-01	cps	1.17E-01	cps	60	20	0	21760	25.10.18	14:44
43398.61505	1.56E+02	cps	1.76E+02	cps	4.52E-01	μSv/h	5.09E-01	μSv/h	6.67E-02	cps	1.33E-01	cps	60	20	0	21760	25.10.18	14:45
43398.61575	1.62E+02	cps	1.81E+02	cps	4.82E-01	μSv/h	6.32E-01	μSv/h	2.00E-01	cps	1.87E-01	cps	60	20	0	21760	25.10.18	14:46
43398.61645	1.53E+02	cps	1.73E+02	cps	4.26E-01	μSv/h	5.63E-01	μSv/h	8.33E-02	cps	1.92E-01	cps	60	20	0	21760	25.10.18	14:47
43398.61715	1.66E+02	cps	1.99E+02	cps	4.71E-01	μSv/h	6.57E-01	μSv/h	1.00E-01	cps	1.61E-01	cps	60	20	0	21760	25.10.18	14:48
43398.61785	1.97E+02	cps	2.51E+02	cps	5.21E-01	μSv/h	7.00E-01	μSv/h	1.00E-01	cps	1.40E-01	cps	60	20	0	21760	25.10.18	14:49
43398.61854	1.77E+02	cps	2.11E+02	cps	4.59E-01	μSv/h	5.61E-01	μSv/h	1.33E-01	cps	1.79E-01	cps	60	20	0	21760	25.10.18	14:50
43398.61924	1.72E+02	cps	2.66E+02	cps	4.68E-01	μSv/h	6.43E-01	μSv/h	1.67E-02	cps	1.02E-01	cps	60	20	0	21760	25.10.18	14:51
43398.61993	1.78E+02	cps	2.39E+02	cps	4.58E-01	μSv/h	5.74E-01	μSv/h	1.33E-01	cps	1.17E-01	cps	60	20	0	21760	25.10.18	14:52
43398.62063	2.87E+02	cps	3.41E+02	cps	8.90E-01	μSv/h	1.07E+00	μSv/h	1.33E-01	cps	1.61E-01	cps	60	20	0	21760	25.10.18	14:53
43398.62131	2.39E+02	cps	2.99E+02	cps	6.60E-01	μSv/h	1.02E+00	μSv/h	6.67E-02	cps	1.17E-01	cps	60	20	0	21760	25.10.18	14:54
43398.622	1.79E+02	cps	2.00E+02	cps	4.68E-01	μSv/h	5.68E-01	μSv/h	3.33E-02	cps	9.45E-02	cps	60	20	0	21760	25.10.18	14:55
43398.6227	2.09E+02	cps	2.30E+02	cps	6.34E-01	μSv/h	6.76E-01	μSv/h	1.00E-01	cps	9.73E-02	cps	60	20	0	21760	25.10.18	14:56
43398.62339	2.21E+02	cps	2.70E+02	cps	6.68E-01	μSv/h	7.32E-01	μSv/h	6.67E-02	cps	1.04E-01	cps	60	20	0	21760	25.10.18	14:57
43398.62409	2.09E+02	cps	2.30E+02	cps	6.05E-01	μSv/h	6.49E-01	μSv/h	0.00E+00	cps	6.23E-02	cps	60	20	0	21760	25.10.18	14:58
43398.62479	2.21E+02	cps	2.53E+02	cps	6.27E-01	μSv/h	7.40E-01	μSv/h	8.33E-02	cps	1.12E-01	cps	60	20	0	21760	25.10.18	14:59
43398.62548	2.24E+02	cps	2.54E+02	cps	6.49E-01	μSv/h	6.91E-01	μSv/h	1.00E-01	cps	1.57E-01	cps	60	20	0	21760	25.10.18	15:00
43398.62618	2.35E+02	cps	2.59E+02	cps	6.82E-01	μSv/h	7.60E-01	μSv/h	1.00E-01	cps	1.20E-01	cps	60	20	0	21760	25.10.18	15:01
43398.62686	2.43E+02	cps	3.05E+02	cps	7.03E-01	μSv/h	1.03E+00	μSv/h	1.50E-01	cps	1.48E-01	cps	60	20	0	21760	25.10.18	15:02
43398.62756	2.46E+02	cps	2.78E+02	cps	7.01E-01	μSv/h	7.95E-01	μSv/h	6.67E-02	cps	1.27E-01	cps	60	20	0	21760	25.10.18	15:03
43398.62826	2.13E+02	cps	2.41E+02	cps	5.89E-01	μSv/h	6.81E-01	μSv/h	1.67E-01	cps	1.56E-01	cps	60	20	0	21760	25.10.18	15:04
43398.62896	2.48E+02	cps	3.90E+02	cps	6.97E-01	μSv/h	1.24E+00	μSv/h	6.67E-02	cps	1.31E-01	cps	60	20	0	21760	25.10.18	15:05
43398.62965	3.32E+02	cps	4.46E+02	cps	1.01E+00	μSv/h	1.54E+00	μSv/h	1.00E-01	cps	1.37E-01	cps	60	20	0	21760	25.10.18	15:06
43398.63035	2.51E+02	cps	2.77E+02	cps	7.39E-01	μSv/h	8.18E-01	μSv/h	1.33E-01	cps	1.49E-01	cps	60	20	0	21760	25.10.18	15:07
43398.63104	2.46E+02	cps	3.07E+02	cps	7.05E-01	μSv/h	8.81E-01	μSv/h	1.67E-02	cps	1.09E-01	cps	60	20	0	21760	25.10.18	15:08
43398.63174	2.60E+02	cps	3.42E+02	cps	7.58E-01	μSv/h	1.06E+00	μSv/h	5.00E-02	cps	6.99E-02	cps	60	20	0	21760	25.10.18	15:09
43398.63242	2.55E+02	cps	3.21E+02	cps	7.50E-01	μSv/h	9.01E-01	μSv/h	1.17E-01	cps	1.20E-01	cps	60	19	0	21760	25.10.18	15:10
43398.63313	2.48E+02	cps	2.74E+02	cps	7.26E-01	μSv/h	7.76E-01	μSv/h	1.17E-01	cps	1.41E-01	cps	60	19	0	21760	25.10.18	15:11
43398.63381	2.72E+02	cps	3.27E+02	cps	7.58E-01	μSv/h	9.14E-01	μSv/h	8.33E-02	cps	1.20E-01	cps	60	19	0	21760	25.10.18	15:12
43398.63451	2.29E+02	cps	2.64E+02	cps	6.16E-01	μSv/h	7.69E-01	μSv/h	3.33E-02	cps	1.04E-01	cps	60	19	0	21760	25.10.18	15:13
43398.6352	2.34E+02	cps	2.66E+02	cps	6.60E-01	μSv/h	7.61E-01	μSv/h	1.00E-01	cps	1.06E-01	cps	60	19	0	21760	25.10.18	15:14
43398.6359	2.37E+02	cps	2.85E+02	cps	6.90E-01	μSv/h	9.04E-01	μSv/h	1.50E-01	cps	1.48E-01	cps	60	19	0	21760	25.10.18	15:15
43398.6366	2.16E+02	cps	2.45E+02	cps	6.34E-01	μSv/h	7.58E-01	μSv/h	1.00E-01	cps	1.73E-01	cps	60	19	0	21760	25.10.18	15:16
43398.63729	2.65E+02	cps	3.03E+02	cps	7.83E-01	μSv/h	9.02E-01	μSv/h	5.00E-02	cps	1.06E-01	cps	60	19	0	21760	25.10.18	15:17
43398.63799	2.73E+02	cps	2.93E+02	cps	8.18E-01	μSv/h	9.37E-01	μSv/h	5.00E-02	cps	7.50E-02	cps	60	19	0	21760	25.10.18	15:18
43398.63868	2.23E+02	cps	2.61E+02	cps	6.14E-01	μSv/h	9.18E-01	μSv/h	1.17E-01	cps	1.00E-01	cps	60	19	0	21760	25.10.18	15:19
43398.63936	2.32E+02	cps	2.50E+02	cps	7.03E-01	μSv/h	8.80E-01	μSv/h	1.00E-01	cps	1.23E-01	cps	60	19	0	21760	25.10.18	15:20
43398.64007	1.86E+02	cps	2.09E+02	cps	5.28E-01	μSv/h	6.14E-01	μSv/h	1.00E-01	cps	1.47E-01	cps	60	19	0	21760	25.10.18	15:21
43398.64076	1.85E+02	cps	1.96E+02	cps	5.22E-01	μSv/h	5.48E-01	μSv/h	1.00E-01	cps	1.21E-01	cps	60	19	0	21760	25.10.18	15:22
43398.64146	1.95E+02	cps	2.03E+02	cps	5.42E-01	μSv/h	5.61E-01	μSv/h	1.00E-01	cps	1.41E-01	cps	60	19	0	21760	25.10.18	15:23
43398.64215	1.91E+02	cps	2.20E+02	cps	5.37E-01	μSv/h	6.01E-01	μSv/h	6.67E-02	cps	1.03E-01	cps	60	19	0	21760	25.10.18	15:24
43398.64282	1.76E+02	cps	2.27E+02	cps	4.62E-01	μSv/h	6.68E-01	μSv/h	8.33E-02	cps	9.75E-02	cps	60	19	0	21760	25.10.18	15:25
43398.64352	1.62E+02	cps	1.88E+02	cps	4.06E-01	μSv/h	4.48E-01	μSv/h	8.33E-02	cps	9.58E-02	cps	60	19	0	21760	25.10.18	15:26
43398.64423	1.67E+02	cps	1.85E+02	cps	4.32E-01	μSv/h	5.71E-01	μSv/h	8.33E-02	cps	9.39E-02	cps	60	19	0	21760	25.10.18	15:27
43398.64493	1.74E+02	cps	1.89E+02	cps	4.36E-01	μSv/h	4.72E-01	μSv/h	8.33E-02	cps	1.03E-01	cps	60	19	0	21760	25.10.18	15:28
43398.64561	1.82E+02	cps	1.98E+02	cps	4.89E-01	μSv/h	5.78E-01	μSv/h	1.17E-01	cps	1.30E-01	cps	60	19	0	21760	25.10.18	15:29
43398.64632	1.91E+02	cps	2.05E+02	cps	4.94E-01	μSv/h	5.53E-01	μSv/h	8.33E-02	cps	1.15E-01	cps	60	19	0	21760	25.10.18	15:30
43398.647	2.16E+02	cps	2.41E+02	cps	5.55E-01	μSv/h	6.11E-01	μSv/h	3.33E-02	cps	9.22E-02	cps	60	19	0	21760	25.10.18	15:31
43398.64771	2.12E+02	cps	2.30E+02	cps	5.60E-01	μSv/h	6.57E-01	μSv/h	5.00E-02	cps	6.86E-02	cps	60	19	0	21760	25.10.18	15:32
43398.6484	1.82E+02	cps	2.03E+02	cps	4.48E-01	μSv/h	6.14E-01	μSv/h	5.00E-02	cps	8.01E-02	cps	60	19	0	21760	25.10.18	15:33
43398.64909	1.99E+02	cps	2.41E+02	cps	5.23E-01	μSv/h	6.58E-01	μSv/h	8.33E-02	cps	8.98E-02	cps	60	19	0	21760	25.10.18	15:34
43398.64979	1.98E+02	cps	2.24E+02	cps	5.15E-01	μSv/h	5.66E-01	μSv/h	8.33E-02	cps	1.03E-01	cps	60	19	0	21760	25.10.18	15:35
43398.65049	2.19E+02	cps	2.30E+02	cps	5.74E-01	μSv/h	6.16E-01	μSv/h	8.33E-02	cps	1.21E-01	cps	60	19	0	21760	25.10.18	15:36
43398.65118	2.23E+02	cps	2.48E+02	cps	5.85E-01	μSv/h	6.31E-01	μSv/h	8.33E-02	cps	9.69E-02	cps	60	19	0	21760	25.10.18	15:37
43398.65186	1.90E+02	cps	2.14E+02	cps	4.94E-01	μSv/h	5.82E-01	μSv/h	6.67E-02	cps	9.14E-02	cps	60	19	0	21760	25.10.18	15:38
43398.65257	1.82E+02	cps	1.91E+02	cps	4.72E-01	μSv/h	5.03E-01	μSv/h	1.00E-01	cps	1.13E-01	cps	60	19	0	21760	25.10.18	15:39
43398.65326	1.80E+02	cps	1.91E+02	cps	4.93E-01	μSv/h	6.29E-01	μSv/h	8.33E-02	cps	1.21E-01	cps	60	19	0	21760	25.10.18	15:40
43398.65396	1.91E+02	cps	2.12E+02	cps	5.34E-01	μSv/h	5.96E-01	μSv/h	5.00E-02	cps	8.83E-02	cps	60	19	0	21760	25.10.18	15:41
43398.65465	1.89E+02	cps	2.06E+02	cps	5.26E-01	μSv/h	5.68E-01	μSv/h	8.33E-02	cps	9.24E-02	cps	60	19	0	21760	25.10.18	15:42
43398.65535	1.85E+02	cps	2.09E+02	cps	4.90E-01	μSv/h	5.57E-01	μSv/h	1.00E-01	cps	1.14E-01	cps	60	19	0	21760	25.10.18	15:43
43398.65603	1.94E+02	cps	2.11E+02	cps	4.89E-01	μSv/h	5.25E-01	μSv/h	0.00E+00	cps	9.94E-02	cps	60	19	0	21760	25.10.18	15:44
43398.65674	1.71E+02	cps	1.88E+02	cps	4.23E-01	μSv/h	4.72E-01	μSv/h	6.67E-02	cps	6.55E-02	cps	60	19	0	21760	25.10.18	15:45
43398.65742	1.69E+02	cps	2.12E+02	cps	4.10E-01	μSv/h	4.76E-01	μSv/h	8.33E-02	cps	1.08E-01	cps	60	19	0	21760	25.10.18	15:46
43398.65809	1.66E+02	cps	1.97E+02	cps	3.91E-01	μSv/h	5.01E-01	μSv/h	1.00E-01	cps	1.10E-01	cps	60	19	0	21760	25.10.18	15:47
43398.65882	1.67E+02	cps	1.96E+02	cps	4.31E-01	μSv/h	5.87E-01	μSv/h	1.17E-01	cps	1.37E-01	cps	60	19	0	21760	25.10.18	15:48
43398.65951	1.62E+02	cps	1.73E+02	cps	4.02E-01	μSv/h	4.79E-01	μSv/h	3.33E-02	cps	1.08E-01	cps	60	18	0	21760	25.10.18	15:49
43398.6602	1.70E+02	cps	1.83E+02	cps	4.63E-01	μSv/h	4.73E-01	μSv/h	6.67E-02	cps	7.72E-02	cps	60	18	0	21760	25.10.18	15:50
43398.6609	1.60E+02	cps	1.74E+02	cps	4.09E-01	μSv/h	4.60E-01	μSv/h	5.00E-02	cps	6.60E-02	cps	60	18	0	21760	25.10.18	15:51
43398.6616	1.78E+02	cps	1.98E+02	cps	4.81E-01	μSv/h	5.94E-01	μSv/h	1.00E-01	cps	1.16E-01	cps	60	18	0	21760	25.10.18	15:52
43398.66229	1.65E+02	cps	1.85E+02	cps	4.19E-01	μSv/h	4.95E-01	μSv/h	1.17E-01	cps	1.24E-01	cps	60	18	0	21760	25.10.18	15:53
43398.66299	1.72E+02	cps	1.96E+02	cps	4.70E-01	μSv/h	4.97E-01	μSv/h	8.33E-02	cps	1.36E-01	cps	60	18	0	21760	25.10.18	15:54
43398.66367	1.82E+02	cps	2.18E+02	cps	4.89E-01	μSv/h	5.56E-01	μSv/h	3.33E-02	cps	9.87E-02	cps	60	18	0	21760	25.10.18	15:55
43398.66438	1.79E+02	cps	2.01E+02	cps	4.69E-01	μSv/h	5.52E-01	μSv/h	5.00E-02	cps	7.29E-02	cps	60	18	0	21760	25.10.18	15:56
43398.66506	1.70E+02	cps	2.02E+02	cps	4.73E-01	μSv/h	5.50E-01	μSv/h	1.33E-01	cps	1.21E-01	cps	60	18	0	21760	25.10.18	15:57
43398.66576	1.66E+02	cps	1.84E+02	cps	4.46E-01	μSv/h	5.20E-01	μSv/h	1.00E-01	cps	1.43E-01	cps	60	18	0	21760	25.10.18	15:58
43398.66646	1.80E+02	cps	1.99E+02	cps	4.87E-01	μSv/h	5.31E-01	μSv/h	6.67E-02	cps	1.08E-01	cps	60	18	0	21760	25.10.18	15:59
43398.66714	1.95E+02	cps	2.16E+02	cps	5.07E-01	μSv/h	5.59E-01	μSv/h	1.00E-01	cps	1.35E-01	cps	60	18	0	21760	25.10.18	16:00

Table S3-2. Data sheet for October 10, 2019

RadEye SPRD History	Version 1.42.0	30												
Date	Serial number	Version	Type	Unit GUnit N	Timer interval	Flag 1	Flag 2	Flag 3	Flag 4	Flag Unit G	Flag Unit N	Flag Type	Index Unit	HistCalMode
**10/10/2019**	10109	RadEye SPRD E1.16	Sv/h	cps	60	124	181	0	2056	261	0	48	5	0

Survey	
Average （μSv/h)	0.47
Median （μSv/h)	0.45
90th percentile	0.63
Min	0.15
Max	0.89
Estimated annual dose rate （mSv/y)	4.0

Date	Mean G	Unit	Max G/Bgnd G	Unit	**Mean** DR-G[μSv/h]	Unit	**Max** DR[μSv/h]/nc	Unit	N[cps]	Unit	Max N/Bgnd N[cps]	Unit	M. time	Temperature	#	Status	Date [dd.mm.yy]	Time [hh:mm]
43748.56909	8.71E+01	cps	1.05E+02	cps	1.55E-01	μSv/h	1.60E-01	μSv/h	8.33E-02	cps	9.26E-02	cps	60	27	0	21760	10.10.19	13:39
43748.56983	1.20E+02	cps	1.41E+02	cps	2.18E-01	μSv/h	2.80E-01	μSv/h	5.00E-02	cps	7.20E-02	cps	60	27	0	21760	10.10.19	13:40
43748.57052	1.35E+02	cps	2.02E+02	cps	2.72E-01	μSv/h	4.20E-01	μSv/h	5.00E-02	cps	7.07E-02	cps	60	27	0	21760	10.10.19	13:41
43748.57123	1.66E+02	cps	1.95E+02	cps	3.70E-01	μSv/h	4.90E-01	μSv/h	6.67E-02	cps	1.07E-01	cps	60	27	0	21760	10.10.19	13:42
43748.57191	1.99E+02	cps	2.26E+02	cps	4.66E-01	μSv/h	5.36E-01	μSv/h	6.67E-02	cps	8.59E-02	cps	60	27	0	21760	10.10.19	13:43
43748.5726	2.00E+02	cps	2.35E+02	cps	4.87E-01	μSv/h	5.71E-01	μSv/h	1.17E-01	cps	1.21E-01	cps	60	27	0	21760	10.10.19	13:44
43748.5733	1.87E+02	cps	2.55E+02	cps	4.65E-01	μSv/h	5.49E-01	μSv/h	8.33E-02	cps	9.79E-02	cps	60	27	0	21760	10.10.19	13:45
43748.57399	1.60E+02	cps	2.06E+02	cps	3.67E-01	μSv/h	5.06E-01	μSv/h	6.67E-02	cps	9.91E-02	cps	60	27	0	21760	10.10.19	13:46
43748.57469	1.58E+02	cps	2.09E+02	cps	3.92E-01	μSv/h	5.20E-01	μSv/h	6.67E-02	cps	8.85E-02	cps	60	27	0	21760	10.10.19	13:47
43748.57538	1.85E+02	cps	2.14E+02	cps	4.50E-01	μSv/h	4.96E-01	μSv/h	5.00E-02	cps	8.82E-02	cps	60	27	0	21760	10.10.19	13:48
43748.57608	1.76E+02	cps	2.03E+02	cps	4.43E-01	μSv/h	5.34E-01	μSv/h	3.33E-02	cps	6.86E-02	cps	60	27	0	21760	10.10.19	13:49
43748.57677	1.83E+02	cps	2.27E+02	cps	4.52E-01	μSv/h	5.18E-01	μSv/h	1.17E-01	cps	1.09E-01	cps	60	27	0	21760	10.10.19	13:50
43748.57747	1.94E+02	cps	2.49E+02	cps	4.35E-01	μSv/h	5.99E-01	μSv/h	8.33E-02	cps	1.20E-01	cps	60	27	0	21760	10.10.19	13:51
43748.57816	1.92E+02	cps	2.67E+02	cps	4.11E-01	μSv/h	6.07E-01	μSv/h	6.67E-02	cps	9.47E-02	cps	60	26	0	21760	10.10.19	13:52
43748.57885	1.75E+02	cps	1.93E+02	cps	3.44E-01	μSv/h	3.78E-01	μSv/h	5.00E-02	cps	7.60E-02	cps	60	26	0	21760	10.10.19	13:53
43748.57955	1.89E+02	cps	2.63E+02	cps	3.76E-01	μSv/h	4.64E-01	μSv/h	3.33E-02	cps	7.83E-02	cps	60	26	0	21760	10.10.19	13:54
43748.58024	2.30E+02	cps	2.80E+02	cps	4.93E-01	μSv/h	6.00E-01	μSv/h	8.33E-02	cps	9.57E-02	cps	60	26	0	21760	10.10.19	13:55
43748.58094	2.78E+02	cps	3.13E+02	cps	6.57E-01	μSv/h	7.23E-01	μSv/h	1.17E-01	cps	1.22E-01	cps	60	26	0	21760	10.10.19	13:56
43748.58163	2.04E+02	cps	2.49E+02	cps	4.15E-01	μSv/h	5.57E-01	μSv/h	1.67E-02	cps	1.28E-01	cps	60	26	0	21760	10.10.19	13:57
43748.58233	1.96E+02	cps	2.18E+02	cps	4.31E-01	μSv/h	4.94E-01	μSv/h	8.33E-02	cps	8.26E-02	cps	60	25	0	21760	10.10.19	13:58
43748.58302	2.20E+02	cps	2.69E+02	cps	5.07E-01	μSv/h	6.19E-01	μSv/h	3.33E-02	cps	8.04E-02	cps	60	25	0	21760	10.10.19	13:59
43748.58374	2.19E+02	cps	2.37E+02	cps	4.88E-01	μSv/h	7.50E-01	μSv/h	5.00E-02	cps	7.12E-02	cps	60	25	0	21760	10.10.19	14:00
43748.58442	2.10E+02	cps	2.35E+02	cps	4.66E-01	μSv/h	5.22E-01	μSv/h	1.17E-01	cps	1.12E-01	cps	60	25	0	21760	10.10.19	14:01
43748.58512	2.23E+02	cps	2.55E+02	cps	4.83E-01	μSv/h	5.45E-01	μSv/h	8.33E-02	cps	1.21E-01	cps	60	25	0	21760	10.10.19	14:02
43748.58581	2.32E+02	cps	2.50E+02	cps	5.16E-01	μSv/h	5.57E-01	μSv/h	8.33E-02	cps	9.43E-02	cps	60	25	0	21760	10.10.19	14:03
43748.58652	2.44E+02	cps	2.77E+02	cps	5.31E-01	μSv/h	6.36E-01	μSv/h	5.00E-02	cps	9.68E-02	cps	60	25	0	21760	10.10.19	14:04
43748.5872	2.60E+02	cps	3.08E+02	cps	5.83E-01	μSv/h	6.78E-01	μSv/h	3.33E-02	cps	7.57E-02	cps	60	24	0	21760	10.10.19	14:05
43748.58789	2.63E+02	cps	3.13E+02	cps	6.35E-01	μSv/h	7.11E-01	μSv/h	6.67E-02	cps	6.30E-02	cps	60	24	0	21760	10.10.19	14:06
43748.5886	2.26E+02	cps	2.52E+02	cps	5.08E-01	μSv/h	6.32E-01	μSv/h	3.33E-02	cps	7.93E-02	cps	60	24	0	21760	10.10.19	14:07
43748.58928	2.26E+02	cps	2.65E+02	cps	4.85E-01	μSv/h	5.45E-01	μSv/h	1.00E-01	cps	9.78E-02	cps	60	24	0	21760	10.10.19	14:08
43748.58998	3.67E+02	cps	5.18E+02	cps	8.94E-01	μSv/h	1.22E+00	μSv/h	1.00E-01	cps	1.15E-01	cps	60	24	0	21760	10.10.19	14:09
43748.59067	3.04E+02	cps	3.52E+02	cps	7.29E-01	μSv/h	8.79E-01	μSv/h	5.00E-02	cps	9.25E-02	cps	60	24	0	21760	10.10.19	14:10
43748.59137	2.79E+02	cps	3.03E+02	cps	6.52E-01	μSv/h	7.46E-01	μSv/h	8.33E-02	cps	1.06E-01	cps	60	24	0	21760	10.10.19	14:11
43748.59207	2.73E+02	cps	2.95E+02	cps	5.86E-01	μSv/h	6.38E-01	μSv/h	1.17E-01	cps	1.37E-01	cps	60	24	0	21760	10.10.19	14:12
43748.59276	2.72E+02	cps	2.94E+02	cps	5.85E-01	μSv/h	6.89E-01	μSv/h	8.33E-02	cps	1.10E-01	cps	60	24	0	21760	10.10.19	14:13
43748.59345	2.59E+02	cps	3.08E+02	cps	5.91E-01	μSv/h	8.28E-01	μSv/h	1.17E-01	cps	1.30E-01	cps	60	24	0	21760	10.10.19	14:14
43748.59414	2.74E+02	cps	3.77E+02	cps	6.60E-01	μSv/h	7.88E-01	μSv/h	1.00E-01	cps	1.34E-01	cps	60	24	0	21760	10.10.19	14:15
43748.59483	2.37E+02	cps	2.71E+02	cps	5.27E-01	μSv/h	7.77E-01	μSv/h	1.50E-01	cps	2.49E-01	cps	60	24	0	21760	10.10.19	14:16
43748.59554	2.54E+02	cps	3.04E+02	cps	5.70E-01	μSv/h	7.22E-01	μSv/h	6.67E-02	cps	2.22E-01	cps	60	24	0	21760	10.10.19	14:17
43748.59623	2.88E+02	cps	3.30E+02	cps	6.63E-01	μSv/h	7.59E-01	μSv/h	5.00E-02	cps	1.10E-01	cps	60	24	0	21760	10.10.19	14:18
43748.59692	2.56E+02	cps	2.81E+02	cps	5.46E-01	μSv/h	6.41E-01	μSv/h	1.67E-02	cps	5.37E-02	cps	60	24	0	21760	10.10.19	14:19
43748.59762	2.72E+02	cps	3.53E+02	cps	6.05E-01	μSv/h	7.67E-01	μSv/h	5.00E-02	cps	5.72E-02	cps	60	24	0	21760	10.10.19	14:20
43748.59831	2.66E+02	cps	3.40E+02	cps	6.23E-01	μSv/h	7.60E-01	μSv/h	1.17E-01	cps	1.25E-01	cps	60	24	0	21760	10.10.19	14:21
43748.59902	2.47E+02	cps	2.71E+02	cps	5.41E-01	μSv/h	5.67E-01	μSv/h	5.00E-02	cps	1.19E-01	cps	60	24	0	21760	10.10.19	14:22
43748.59969	2.50E+02	cps	3.14E+02	cps	5.51E-01	μSv/h	7.69E-01	μSv/h	6.67E-02	cps	7.93E-02	cps	60	24	0	21760	10.10.19	14:23
43748.60041	2.40E+02	cps	2.59E+02	cps	5.44E-01	μSv/h	5.72E-01	μSv/h	1.17E-01	cps	1.16E-01	cps	60	24	0	21760	10.10.19	14:24
43748.60109	2.27E+02	cps	2.45E+02	cps	4.98E-01	μSv/h	5.57E-01	μSv/h	6.67E-02	cps	1.33E-01	cps	60	24	0	21760	10.10.19	14:25
43748.60178	2.84E+02	cps	3.27E+02	cps	6.47E-01	μSv/h	7.21E-01	μSv/h	3.33E-02	cps	9.42E-02	cps	60	24	0	21760	10.10.19	14:26
43748.60248	3.05E+02	cps	3.53E+02	cps	7.07E-01	μSv/h	7.93E-01	μSv/h	6.67E-02	cps	7.67E-02	cps	60	24	0	21760	10.10.19	14:27
43748.60317	2.75E+02	cps	3.46E+02	cps	6.64E-01	μSv/h	8.60E-01	μSv/h	1.00E-01	cps	1.04E-01	cps	60	23	0	21760	10.10.19	14:28
43748.60387	2.03E+02	cps	2.28E+02	cps	4.70E-01	μSv/h	5.91E-01	μSv/h	1.17E-01	cps	1.23E-01	cps	60	23	0	21760	10.10.19	14:29
43748.60455	1.84E+02	cps	1.99E+02	cps	3.86E-01	μSv/h	4.11E-01	μSv/h	6.67E-02	cps	1.28E-01	cps	60	23	0	21760	10.10.19	14:30
43748.60526	1.94E+02	cps	2.09E+02	cps	4.37E-01	μSv/h	4.97E-01	μSv/h	8.33E-02	cps	1.03E-01	cps	60	23	0	21760	10.10.19	14:31
43748.60596	2.02E+02	cps	2.14E+02	cps	4.52E-01	μSv/h	5.06E-01	μSv/h	8.33E-02	cps	1.06E-01	cps	60	23	0	21760	10.10.19	14:32
43748.60664	2.06E+02	cps	2.23E+02	cps	4.64E-01	μSv/h	5.08E-01	μSv/h	3.33E-02	cps	6.97E-02	cps	60	23	0	21760	10.10.19	14:33
43748.60734	1.99E+02	cps	2.20E+02	cps	4.44E-01	μSv/h	5.12E-01	μSv/h	1.00E-01	cps	1.28E-01	cps	60	23	0	21760	10.10.19	14:34
43748.60803	1.78E+02	cps	2.32E+02	cps	3.71E-01	μSv/h	5.32E-01	μSv/h	1.00E-01	cps	1.10E-01	cps	60	23	0	21760	10.10.19	14:35
43748.60873	1.78E+02	cps	2.00E+02	cps	3.66E-01	μSv/h	4.74E-01	μSv/h	1.50E-01	cps	1.46E-01	cps	60	23	0	21760	10.10.19	14:36
43748.60943	1.86E+02	cps	2.02E+02	cps	4.15E-01	μSv/h	4.37E-01	μSv/h	6.67E-02	cps	1.33E-01	cps	60	23	0	21760	10.10.19	14:37
43748.61013	1.93E+02	cps	2.10E+02	cps	4.32E-01	μSv/h	4.74E-01	μSv/h	8.33E-02	cps	9.59E-02	cps	60	23	0	21760	10.10.19	14:38
43748.61082	2.04E+02	cps	2.27E+02	cps	4.36E-01	μSv/h	5.19E-01	μSv/h	1.33E-01	cps	1.37E-01	cps	60	23	0	21760	10.10.19	14:39
43748.61153	2.26E+02	cps	2.51E+02	cps	5.19E-01	μSv/h	5.84E-01	μSv/h	1.33E-01	cps	1.52E-01	cps	60	23	0	21760	10.10.19	14:40
43748.61221	2.07E+02	cps	2.66E+02	cps	4.57E-01	μSv/h	5.72E-01	μSv/h	5.00E-02	cps	1.46E-01	cps	60	23	0	21760	10.10.19	14:41
43748.61292	2.05E+02	cps	2.25E+02	cps	4.74E-01	μSv/h	5.06E-01	μSv/h	5.00E-02	cps	8.18E-02	cps	60	23	0	21760	10.10.19	14:42
43748.6136	2.22E+02	cps	2.62E+02	cps	5.23E-01	μSv/h	6.05E-01	μSv/h	8.33E-02	cps	9.72E-02	cps	60	23	0	21760	10.10.19	14:43
43748.61431	2.25E+02	cps	2.60E+02	cps	4.97E-01	μSv/h	5.54E-01	μSv/h	1.33E-01	cps	1.35E-01	cps	60	23	0	21760	10.10.19	14:44
43748.61499	2.30E+02	cps	2.74E+02	cps	5.11E-01	μSv/h	6.00E-01	μSv/h	6.67E-02	cps	1.30E-01	cps	60	23	0	21760	10.10.19	14:45
43748.61568	1.89E+02	cps	2.04E+02	cps	3.74E-01	μSv/h	4.12E-01	μSv/h	3.33E-02	cps	7.29E-02	cps	60	23	0	21760	10.10.19	14:46
43748.61637	1.89E+02	cps	2.02E+02	cps	4.12E-01	μSv/h	4.76E-01	μSv/h	1.17E-01	cps	1.15E-01	cps	60	23	0	21760	10.10.19	14:47
43748.61707	1.89E+02	cps	2.06E+02	cps	4.21E-01	μSv/h	4.33E-01	μSv/h	1.00E-01	cps	1.57E-01	cps	60	23	0	21760	10.10.19	14:48
43748.61777	1.84E+02	cps	2.01E+02	cps	3.81E-01	μSv/h	4.48E-01	μSv/h	1.67E-02	cps	1.13E-01	cps	60	23	0	21760	10.10.19	14:49
43748.61847	1.73E+02	cps	1.94E+02	cps	3.25E-01	μSv/h	3.53E-01	μSv/h	1.50E-01	cps	1.22E-01	cps	60	23	0	21760	10.10.19	14:50
43748.61916	1.92E+02	cps	2.35E+02	cps	3.92E-01	μSv/h	4.69E-01	μSv/h	8.33E-02	cps	1.19E-01	cps	60	23	0	21760	10.10.19	14:51
43748.61984	2.00E+02	cps	2.18E+02	cps	4.33E-01	μSv/h	4.56E-01	μSv/h	6.67E-02	cps	1.10E-01	cps	60	23	0	21760	10.10.19	14:52
43748.62054	1.92E+02	cps	2.13E+02	cps	4.03E-01	μSv/h	4.32E-01	μSv/h	5.00E-02	cps	8.70E-02	cps	60	23	0	21760	10.10.19	14:53
43748.62124	2.01E+02	cps	2.13E+02	cps	4.54E-01	μSv/h	5.19E-01	μSv/h	8.33E-02	cps	8.74E-02	cps	60	23	0	21760	10.10.19	14:54
43748.62193	1.81E+02	cps	2.02E+02	cps	4.16E-01	μSv/h	4.62E-01	μSv/h	5.00E-02	cps	8.05E-02	cps	60	23	0	21760	10.10.19	14:55
43748.62264	1.72E+02	cps	1.99E+02	cps	3.55E-01	μSv/h	4.61E-01	μSv/h	6.67E-02	cps	8.94E-02	cps	60	23	0	21760	10.10.19	14:56
43748.62332	1.84E+02	cps	2.00E+02	cps	3.98E-01	μSv/h	4.15E-01	μSv/h	8.33E-02	cps	9.42E-02	cps	60	23	0	21760	10.10.19	14:57
43748.62403	1.77E+02	cps	1.83E+02	cps	3.68E-01	μSv/h	4.31E-01	μSv/h	1.00E-01	cps	1.10E-01	cps	60	23	0	21760	10.10.19	14:58
43748.62471	1.80E+02	cps	2.08E+02	cps	3.90E-01	μSv/h	4.74E-01	μSv/h	8.33E-02	cps	1.18E-01	cps	60	23	0	21760	10.10.19	14:59
43748.62539	1.75E+02	cps	1.85E+02	cps	3.83E-01	μSv/h	4.08E-01	μSv/h	1.33E-01	cps	1.36E-01	cps	60	23	0	21760	10.10.19	15:00
43748.6261	1.75E+02	cps	1.94E+02	cps	3.91E-01	μSv/h	4.48E-01	μSv/h	6.67E-02	cps	1.04E-01	cps	60	23	0	21760	10.10.19	15:01
43748.62681	1.74E+02	cps	1.87E+02	cps	3.76E-01	μSv/h	4.11E-01	μSv/h	5.00E-02	cps	9.26E-02	cps	60	23	0	21760	10.10.19	15:02
43748.62749	1.86E+02	cps	2.07E+02	cps	4.34E-01	μSv/h	4.84E-01	μSv/h	1.17E-01	cps	1.33E-01	cps	60	22	0	21760	10.10.19	15:03
43748.62818	1.97E+02	cps	2.28E+02	cps	4.82E-01	μSv/h	5.25E-01	μSv/h	1.00E-01	cps	1.10E-01	cps	60	22	0	21760	10.10.19	15:04
43748.62888	1.84E+02	cps	2.33E+02	cps	4.27E-01	μSv/h	5.67E-01	μSv/h	5.00E-02	cps	9.57E-02	cps	60	22	0	21760	10.10.19	15:05
43748.62957	1.92E+02	cps	2.34E+02	cps	4.32E-01	μSv/h	4.82E-01	μSv/h	5.00E-02	cps	6.50E-02	cps	60	22	0	21760	10.10.19	15:06
43748.63028	1.83E+02	cps	2.05E+02	cps	4.24E-01	μSv/h	4.71E-01	μSv/h	1.17E-01	cps	1.49E-01	cps	60	22	0	21760	10.10.19	15:07
43748.63096	1.76E+02	cps	2.28E+02	cps	4.00E-01	μSv/h	4.41E-01	μSv/h	5.00E-02	cps	1.04E-01	cps	60	22	0	21760	10.10.19	15:08
43748.63164	1.90E+02	cps	2.15E+02	cps	4.49E-01	μSv/h	5.61E-01	μSv/h	1.00E-01	cps	1.09E-01	cps	60	22	0	21760	10.10.19	15:09
43748.63236	1.99E+02	cps	2.14E+02	cps	4.65E-01	μSv/h	5.38E-01	μSv/h	8.33E-02	cps	1.17E-01	cps	60	22	0	21760	10.10.19	15:10
43748.63306	2.16E+02	cps	2.43E+02	cps	5.17E-01	μSv/h	6.20E-01	μSv/h	6.67E-02	cps	1.09E-01	cps	60	22	0	21760	10.10.19	15:11
43748.63374	1.69E+02	cps	2.03E+02	cps	3.61E-01	μSv/h	4.80E-01	μSv/h	5.00E-02	cps	8.37E-02	cps	60	22	0	21760	10.10.19	15:12

Table S3-3. Data sheet for October 17, 2019

RadEye SPRD History	Version 1.42.0	30													**Survey**	
Date	Serial number	Version	Type	Unit GUnit N	Timer interval	Flag 1	Flag 2	Flag 3	Flag 4	Flag Unit G	Flag Unit N	Flag Type	Index Unit	HistCalMode	Average (μSv/h)	0.48
**17/10/2019**	10109	RadEye SPRD E1.16	Sv/h	cps	60	124	181	0	2056	261	0	48	5	0	Median (μSv/h)	0.45
															90th percentile	0.67
															Min	0.22
															Max	0.92
															Estimated annual dose rate (mSv/y)	3.9

Date	Mean G	Unit	Max G/Bgnd G	Unit	**Mean** DR-G[μSv/h]	Unit	**Max** DR[μSv/h]/nc	Unit	N[cps]	Unit	Max N/Bgnd N[cps]	Unit	M. time	Temperature	#	Status	Date [dd.mm.yy]	Time [hh:mm]
43755.62229	1.28E+02	cps	1.46E+02	cps	2.44E-01	μSv/h	3.07E-01	μSv/h	3.33E-02	cps	1.75E-01	cps	60	26	0	21760	17.10.19	14:56
43755.62298	1.16E+02	cps	1.42E+02	cps	2.41E-01	μSv/h	3.26E-01	μSv/h	8.33E-02	cps	8.89E-02	cps	60	26	0	21760	17.10.19	14:57
43755.62368	1.21E+02	cps	1.91E+02	cps	2.23E-01	μSv/h	4.16E-01	μSv/h	6.67E-02	cps	9.13E-02	cps	60	25	0	21760	17.10.19	14:58
43755.62438	1.75E+02	cps	2.18E+02	cps	4.12E-01	μSv/h	4.70E-01	μSv/h	1.00E-01	cps	1.22E-01	cps	60	25	0	21760	17.10.19	14:59
43755.62507	2.05E+02	cps	2.56E+02	cps	5.31E-01	μSv/h	7.03E-01	μSv/h	1.17E-01	cps	1.34E-01	cps	60	25	0	21760	17.10.19	15:00
43755.62575	1.87E+02	cps	3.04E+02	cps	4.82E-01	μSv/h	7.70E-01	μSv/h	1.00E-01	cps	1.35E-01	cps	60	25	0	21760	17.10.19	15:01
43755.62645	1.46E+02	cps	1.85E+02	cps	3.62E-01	μSv/h	4.88E-01	μSv/h	1.33E-01	cps	1.60E-01	cps	60	24	0	21760	17.10.19	15:02
43755.62715	1.77E+02	cps	2.08E+02	cps	4.72E-01	μSv/h	5.25E-01	μSv/h	8.33E-02	cps	1.18E-01	cps	60	24	0	21760	17.10.19	15:03
43755.62784	1.73E+02	cps	1.97E+02	cps	4.32E-01	μSv/h	4.71E-01	μSv/h	5.00E-02	cps	9.16E-02	cps	60	24	0	21760	17.10.19	15:04
43755.62854	1.72E+02	cps	2.27E+02	cps	4.46E-01	μSv/h	5.76E-01	μSv/h	8.33E-02	cps	8.37E-02	cps	60	24	0	21760	17.10.19	15:05
43755.62924	2.14E+02	cps	2.58E+02	cps	5.24E-01	μSv/h	6.05E-01	μSv/h	5.00E-02	cps	9.12E-02	cps	60	24	0	21760	17.10.19	15:06
43755.62993	1.92E+02	cps	2.62E+02	cps	4.51E-01	μSv/h	6.34E-01	μSv/h	6.67E-02	cps	9.08E-02	cps	60	24	0	21760	17.10.19	15:07
43755.63064	1.61E+02	cps	1.94E+02	cps	3.54E-01	μSv/h	4.45E-01	μSv/h	1.33E-01	cps	1.27E-01	cps	60	23	0	21760	17.10.19	15:08
43755.63132	2.00E+02	cps	2.55E+02	cps	4.49E-01	μSv/h	5.98E-01	μSv/h	1.50E-01	cps	1.66E-01	cps	60	23	0	21760	17.10.19	15:09
43755.63203	2.72E+02	cps	3.07E+02	cps	6.31E-01	μSv/h	7.43E-01	μSv/h	5.00E-02	cps	1.23E-01	cps	60	23	0	21760	17.10.19	15:10
43755.63272	1.94E+02	cps	2.54E+02	cps	4.42E-01	μSv/h	5.67E-01	μSv/h	5.00E-02	cps	8.55E-02	cps	60	23	0	21760	17.10.19	15:11
43755.63341	2.02E+02	cps	2.21E+02	cps	4.72E-01	μSv/h	5.26E-01	μSv/h	5.00E-02	cps	8.51E-02	cps	60	23	0	21760	17.10.19	15:12
43755.6341	2.12E+02	cps	2.41E+02	cps	4.87E-01	μSv/h	5.47E-01	μSv/h	8.33E-02	cps	9.14E-02	cps	60	23	0	21760	17.10.19	15:13
43755.63479	2.08E+02	cps	2.36E+02	cps	4.72E-01	μSv/h	5.18E-01	μSv/h	8.33E-02	cps	8.82E-02	cps	60	23	0	21760	17.10.19	15:14
43755.63549	2.17E+02	cps	2.38E+02	cps	4.95E-01	μSv/h	5.33E-01	μSv/h	5.00E-02	cps	9.18E-02	cps	60	22	0	21760	17.10.19	15:15
43755.63619	2.21E+02	cps	2.48E+02	cps	5.30E-01	μSv/h	5.87E-01	μSv/h	1.00E-01	cps	1.01E-01	cps	60	22	0	21760	17.10.19	15:16
43755.63689	2.31E+02	cps	2.99E+02	cps	5.63E-01	μSv/h	7.21E-01	μSv/h	1.17E-01	cps	1.25E-01	cps	60	22	0	21760	17.10.19	15:17
43755.63757	2.50E+02	cps	2.95E+02	cps	6.09E-01	μSv/h	6.81E-01	μSv/h	6.67E-02	cps	1.12E-01	cps	60	22	0	21760	17.10.19	15:18
43755.63824	2.56E+02	cps	3.21E+02	cps	6.53E-01	μSv/h	8.64E-01	μSv/h	5.00E-02	cps	9.28E-02	cps	60	22	0	21760	17.10.19	15:19
43755.63897	2.21E+02	cps	2.51E+02	cps	5.33E-01	μSv/h	5.75E-01	μSv/h	1.17E-01	cps	1.00E-01	cps	60	22	0	21760	17.10.19	15:20
43755.63965	3.49E+02	cps	5.13E+02	cps	9.23E-01	μSv/h	1.54E+00	μSv/h	1.67E-02	cps	9.24E-02	cps	60	22	0	21760	17.10.19	15:21
43755.64036	2.97E+02	cps	3.47E+02	cps	7.85E-01	μSv/h	8.60E-01	μSv/h	8.33E-02	cps	1.06E-01	cps	60	22	0	21760	17.10.19	15:22
43755.64107	2.51E+02	cps	2.81E+02	cps	6.78E-01	μSv/h	1.54E+00	μSv/h	1.00E-01	cps	9.74E-02	cps	60	22	0	21760	17.10.19	15:23
43755.64175	2.65E+02	cps	3.63E+02	cps	6.71E-01	μSv/h	1.01E+00	μSv/h	1.67E-01	cps	1.79E-01	cps	60	21	0	21760	17.10.19	15:24
43755.64244	2.78E+02	cps	3.00E+02	cps	6.71E-01	μSv/h	6.97E-01	μSv/h	6.67E-02	cps	1.15E-01	cps	60	21	0	21760	17.10.19	15:25
43755.64315	2.88E+02	cps	3.16E+02	cps	7.24E-01	μSv/h	7.74E-01	μSv/h	8.33E-02	cps	1.14E-01	cps	60	21	0	21760	17.10.19	15:26
43755.64383	2.52E+02	cps	2.92E+02	cps	6.59E-01	μSv/h	7.81E-01	μSv/h	1.00E-01	cps	1.23E-01	cps	60	21	0	21760	17.10.19	15:27
43755.64454	2.70E+02	cps	3.31E+02	cps	7.00E-01	μSv/h	8.11E-01	μSv/h	5.00E-02	cps	9.64E-02	cps	60	21	0	21764	17.10.19	15:28
43755.64522	2.50E+02	cps	3.06E+02	cps	5.88E-01	μSv/h	7.98E-01	μSv/h	1.00E-01	cps	1.14E-01	cps	60	21	0	21764	17.10.19	15:29
43755.64593	2.48E+02	cps	2.85E+02	cps	5.53E-01	μSv/h	5.87E-01	μSv/h	1.00E-01	cps	1.29E-01	cps	60	21	0	21764	17.10.19	15:30
43755.64661	2.43E+02	cps	3.09E+02	cps	5.68E-01	μSv/h	8.36E-01	μSv/h	3.33E-02	cps	8.32E-02	cps	60	21	0	21764	17.10.19	15:31
43755.64732	2.42E+02	cps	3.01E+02	cps	5.88E-01	μSv/h	6.91E-01	μSv/h	1.67E-02	cps	6.84E-02	cps	60	21	0	21764	17.10.19	15:32
43755.648	3.00E+02	cps	3.33E+02	cps	7.46E-01	μSv/h	8.29E-01	μSv/h	1.00E-01	cps	8.63E-02	cps	60	21	0	21764	17.10.19	15:33
43755.6487	2.80E+02	cps	3.37E+02	cps	6.90E-01	μSv/h	8.67E-01	μSv/h	3.33E-02	cps	8.61E-02	cps	60	21	0	21764	17.10.19	15:34
43755.6494	1.96E+02	cps	2.36E+02	cps	4.42E-01	μSv/h	6.45E-01	μSv/h	1.67E-02	cps	4.36E-02	cps	60	21	0	21764	17.10.19	15:35
43755.65009	1.88E+02	cps	2.02E+02	cps	4.26E-01	μSv/h	4.56E-01	μSv/h	6.67E-02	cps	8.12E-02	cps	60	21	0	21764	17.10.19	15:36
43755.65079	2.01E+02	cps	2.26E+02	cps	4.67E-01	μSv/h	5.54E-01	μSv/h	1.33E-01	cps	1.51E-01	cps	60	21	0	21760	17.10.19	15:37
43755.65148	2.01E+02	cps	2.24E+02	cps	4.27E-01	μSv/h	4.57E-01	μSv/h	3.33E-02	cps	1.10E-01	cps	60	20	0	21760	17.10.19	15:38
43755.65218	1.93E+02	cps	2.26E+02	cps	4.49E-01	μSv/h	6.17E-01	μSv/h	1.17E-01	cps	1.03E-01	cps	60	20	0	21760	17.10.19	15:39
43755.65284	1.63E+02	cps	2.38E+02	cps	3.23E-01	μSv/h	6.05E-01	μSv/h	6.67E-02	cps	1.21E-01	cps	60	20	0	21760	17.10.19	15:40
43755.65355	1.64E+02	cps	1.73E+02	cps	3.33E-01	μSv/h	3.76E-01	μSv/h	1.00E-01	cps	1.13E-01	cps	60	20	0	21760	17.10.19	15:41
43755.65425	1.75E+02	cps	2.09E+02	cps	3.75E-01	μSv/h	7.25E-01	μSv/h	1.50E-01	cps	1.44E-01	cps	60	20	0	21760	17.10.19	15:42
43755.65495	1.90E+02	cps	2.17E+02	cps	3.89E-01	μSv/h	4.26E-01	μSv/h	5.00E-02	cps	1.51E-01	cps	60	20	0	21760	17.10.19	15:43
43755.65565	1.80E+02	cps	1.93E+02	cps	3.70E-01	μSv/h	4.03E-01	μSv/h	6.67E-02	cps	9.71E-02	cps	60	20	0	21760	17.10.19	15:44
43755.65634	1.92E+02	cps	2.12E+02	cps	4.14E-01	μSv/h	4.47E-01	μSv/h	6.67E-02	cps	8.28E-02	cps	60	20	0	21760	17.10.19	15:45
43755.65704	2.04E+02	cps	2.50E+02	cps	4.49E-01	μSv/h	4.78E-01	μSv/h	6.67E-02	cps	8.10E-02	cps	60	20	0	21760	17.10.19	15:46
43755.65773	2.20E+02	cps	2.47E+02	cps	4.83E-01	μSv/h	5.37E-01	μSv/h	1.33E-01	cps	2.57E-01	cps	60	20	0	21760	17.10.19	15:47
43755.65843	1.92E+02	cps	2.07E+02	cps	4.04E-01	μSv/h	4.55E-01	μSv/h	5.00E-02	cps	1.50E-01	cps	60	20	0	21760	17.10.19	15:48
43755.65911	2.18E+02	cps	2.67E+02	cps	4.72E-01	μSv/h	5.94E-01	μSv/h	1.00E-01	cps	1.13E-01	cps	60	20	0	21760	17.10.19	15:49
43755.6598	2.22E+02	cps	2.48E+02	cps	4.93E-01	μSv/h	5.76E-01	μSv/h	8.33E-02	cps	1.11E-01	cps	60	20	0	21760	17.10.19	15:50
43755.66051	1.94E+02	cps	2.18E+02	cps	4.16E-01	μSv/h	4.74E-01	μSv/h	1.17E-01	cps	1.21E-01	cps	60	20	0	21760	17.10.19	15:51
43755.6612	1.88E+02	cps	2.11E+02	cps	3.99E-01	μSv/h	4.46E-01	μSv/h	1.00E-01	cps	1.34E-01	cps	60	20	0	21760	17.10.19	15:52
43755.6619	1.87E+02	cps	2.11E+02	cps	4.21E-01	μSv/h	4.92E-01	μSv/h	6.67E-02	cps	1.15E-01	cps	60	20	0	21760	17.10.19	15:53
43755.66258	1.98E+02	cps	2.26E+02	cps	4.60E-01	μSv/h	6.61E-01	μSv/h	8.33E-02	cps	1.29E-01	cps	60	20	0	21760	17.10.19	15:54
43755.66328	1.96E+02	cps	2.13E+02	cps	4.29E-01	μSv/h	4.91E-01	μSv/h	6.67E-02	cps	9.98E-02	cps	60	20	0	21760	17.10.19	15:55
43755.66397	1.93E+02	cps	2.05E+02	cps	4.38E-01	μSv/h	4.65E-01	μSv/h	1.00E-01	cps	1.36E-01	cps	60	20	0	21760	17.10.19	15:56
43755.66468	1.70E+02	cps	1.83E+02	cps	3.82E-01	μSv/h	4.49E-01	μSv/h	1.17E-01	cps	1.42E-01	cps	60	20	0	21760	17.10.19	15:57
43755.66537	1.58E+02	cps	1.69E+02	cps	3.22E-01	μSv/h	3.75E-01	μSv/h	1.00E-01	cps	1.35E-01	cps	60	20	0	21760	17.10.19	15:58
43755.66608	1.82E+02	cps	2.08E+02	cps	3.97E-01	μSv/h	4.87E-01	μSv/h	3.33E-02	cps	1.07E-01	cps	60	20	0	21760	17.10.19	15:59
43755.66676	1.71E+02	cps	2.10E+02	cps	3.72E-01	μSv/h	4.98E-01	μSv/h	1.67E-02	cps	5.36E-02	cps	60	20	0	21760	17.10.19	16:00
43755.66747	1.72E+02	cps	1.91E+02	cps	3.70E-01	μSv/h	3.95E-01	μSv/h	1.00E-01	cps	8.13E-02	cps	60	20	0	21760	17.10.19	16:01
43755.66815	1.68E+02	cps	1.79E+02	cps	3.41E-01	μSv/h	3.82E-01	μSv/h	1.67E-02	cps	7.51E-02	cps	60	20	0	21760	17.10.19	16:02
43755.66885	1.64E+02	cps	1.85E+02	cps	3.42E-01	μSv/h	4.01E-01	μSv/h	5.00E-02	cps	6.16E-02	cps	60	20	0	21760	17.10.19	16:03
43755.66955	1.82E+02	cps	2.04E+02	cps	3.91E-01	μSv/h	4.12E-01	μSv/h	1.17E-01	cps	1.08E-01	cps	60	20	0	21760	17.10.19	16:04
43755.67024	1.71E+02	cps	1.87E+02	cps	3.77E-01	μSv/h	4.03E-01	μSv/h	3.33E-02	cps	9.54E-02	cps	60	20	0	21760	17.10.19	16:05
43755.67091	1.86E+02	cps	2.14E+02	cps	4.19E-01	μSv/h	5.05E-01	μSv/h	1.17E-01	cps	1.06E-01	cps	60	20	0	21760	17.10.19	16:06
43755.67162	1.76E+02	cps	1.96E+02	cps	3.92E-01	μSv/h	4.49E-01	μSv/h	5.00E-02	cps	1.27E-01	cps	60	20	0	21760	17.10.19	16:07
43755.67233	1.78E+02	cps	2.13E+02	cps	4.14E-01	μSv/h	5.08E-01	μSv/h	1.33E-01	cps	2.60E-01	cps	60	20	0	21760	17.10.19	16:08
43755.67301	1.86E+02	cps	2.11E+02	cps	4.40E-01	μSv/h	5.07E-01	μSv/h	1.17E-01	cps	2.29E-01	cps	60	20	0	21760	17.10.19	16:09
43755.6737	2.02E+02	cps	2.44E+02	cps	4.76E-01	μSv/h	6.16E-01	μSv/h	5.00E-02	cps	1.34E-01	cps	60	20	0	21760	17.10.19	16:10

Table S3-4. Data sheet for October 24, 2019

RadEye SPRD History	Version 1.42.0	30													Survey	
Date	Serial number	Version	Type	Unit GUnit N	Timer interval	Flag 1	Flag 2	Flag 3	Flag 4	Flag Unit G	Flag Unit N	Flag Type	Index Unit	HistCalMode	Average（μSv/h）	0.45
24/10/2019	24/10/2019	RadEye SPRD E1.16	Sv/h	cps	60	124	181	0	2056	261	0	48	5	0	Median（μSv/h）	0.43
															90th percentile	0.58
															Min	0.22
															Max	0.84
															Estimated annual dose rate（mSv/y）	3.8

Date	Mean G	Unit	Max G/Bgnd G	Unit	Mean DR-G[μSv/h]	Unit	Max DR[μSv/h]/nc	Unit	N[cps]	Unit	Max N/Bgnd N[cps]	Unit	M. time	Temperature	#	Status	Date [dd.mm.yy]	Time [hh:mm]
43762.5466	1.25E+02	cps	1.39E+02	cps	2.46E-01	μSv/h	3.25E-01	μSv/h	5.00E-02	cps	8.56E-02	cps	60	25	0	21760	24.10.19	13:07
43762.5473	1.26E+02	cps	1.40E+02	cps	2.65E-01	μSv/h	3.18E-01	μSv/h	3.33E-02	cps	6.86E-02	cps	60	25	0	21760	24.10.19	13:08
43762.54802	1.16E+02	cps	1.67E+02	cps	2.21E-01	μSv/h	4.32E-01	μSv/h	6.67E-02	cps	6.57E-02	cps	60	25	0	21760	24.10.19	13:09
43762.54872	1.72E+02	cps	2.11E+02	cps	3.93E-01	μSv/h	4.79E-01	μSv/h	3.33E-02	cps	7.52E-02	cps	60	24	0	21760	24.10.19	13:10
43762.54941	2.07E+02	cps	2.41E+02	cps	5.36E-01	μSv/h	6.24E-01	μSv/h	1.50E-01	cps	1.37E-01	cps	60	24	0	21760	24.10.19	13:11
43762.5501	1.89E+02	cps	2.78E+02	cps	4.73E-01	μSv/h	7.49E-01	μSv/h	1.17E-01	cps	1.30E-01	cps	60	24	0	21760	24.10.19	13:12
43762.5508	1.68E+02	cps	2.08E+02	cps	3.97E-01	μSv/h	5.29E-01	μSv/h	3.33E-02	cps	1.29E-01	cps	60	24	0	21760	24.10.19	13:13
43762.55149	1.47E+02	cps	1.93E+02	cps	3.46E-01	μSv/h	4.58E-01	μSv/h	1.00E-01	cps	1.00E-01	cps	60	24	0	21760	24.10.19	13:14
43762.55219	1.76E+02	cps	1.99E+02	cps	4.33E-01	μSv/h	4.83E-01	μSv/h	1.00E-01	cps	1.50E-01	cps	60	24	0	21760	24.10.19	13:15
43762.55288	1.70E+02	cps	1.94E+02	cps	4.33E-01	μSv/h	4.79E-01	μSv/h	6.67E-02	cps	9.71E-02	cps	60	23	0	21760	24.10.19	13:16
43762.55357	1.80E+02	cps	2.29E+02	cps	4.58E-01	μSv/h	6.78E-01	μSv/h	6.67E-02	cps	1.06E-01	cps	60	23	0	21760	24.10.19	13:17
43762.55427	1.46E+02	cps	1.63E+02	cps	2.91E-01	μSv/h	3.55E-01	μSv/h	8.33E-02	cps	1.18E-01	cps	60	23	0	21760	24.10.19	13:18
43762.55497	1.69E+02	cps	1.96E+02	cps	3.85E-01	μSv/h	4.39E-01	μSv/h	1.17E-01	cps	1.34E-01	cps	60	23	0	21760	24.10.19	13:19
43762.55566	2.17E+02	cps	2.76E+02	cps	4.96E-01	μSv/h	5.86E-01	μSv/h	1.00E-01	cps	1.24E-01	cps	60	23	0	21760	24.10.19	13:20
43762.55637	1.85E+02	cps	2.51E+02	cps	4.17E-01	μSv/h	6.77E-01	μSv/h	5.00E-02	cps	8.69E-02	cps	60	23	0	21760	24.10.19	13:21
43762.55706	1.70E+02	cps	1.91E+02	cps	3.56E-01	μSv/h	3.98E-01	μSv/h	5.00E-02	cps	7.06E-02	cps	60	22	0	21760	24.10.19	13:22
43762.55776	1.63E+02	cps	1.83E+02	cps	3.59E-01	μSv/h	3.84E-01	μSv/h	1.00E-01	cps	1.19E-01	cps	60	22	0	21760	24.10.19	13:23
43762.55845	1.66E+02	cps	1.91E+02	cps	3.75E-01	μSv/h	4.53E-01	μSv/h	1.17E-01	cps	1.51E-01	cps	60	22	0	21760	24.10.19	13:24
43762.55914	1.65E+02	cps	1.89E+02	cps	3.73E-01	μSv/h	4.44E-01	μSv/h	1.00E-01	cps	1.41E-01	cps	60	22	0	21760	24.10.19	13:25
43762.55984	2.51E+02	cps	3.10E+02	cps	5.62E-01	μSv/h	6.20E-01	μSv/h	1.33E-01	cps	1.35E-01	cps	60	22	0	21760	24.10.19	13:26
43762.56053	2.41E+02	cps	3.41E+02	cps	5.44E-01	μSv/h	8.29E-01	μSv/h	1.50E-01	cps	2.01E-01	cps	60	22	0	21760	24.10.19	13:27
43762.56123	1.82E+02	cps	2.02E+02	cps	4.04E-01	μSv/h	4.78E-01	μSv/h	1.33E-01	cps	1.64E-01	cps	60	22	0	21760	24.10.19	13:28
43762.56191	2.08E+02	cps	2.34E+02	cps	4.74E-01	μSv/h	5.21E-01	μSv/h	3.33E-02	cps	1.16E-01	cps	60	22	0	21760	24.10.19	13:29
43762.5626	2.15E+02	cps	2.43E+02	cps	5.03E-01	μSv/h	5.61E-01	μSv/h	8.33E-02	cps	8.71E-02	cps	60	22	0	21760	24.10.19	13:30
43762.56331	2.01E+02	cps	2.26E+02	cps	4.21E-01	μSv/h	5.10E-01	μSv/h	5.00E-02	cps	7.41E-02	cps	60	21	0	21760	24.10.19	13:31
43762.56401	2.16E+02	cps	2.38E+02	cps	4.75E-01	μSv/h	5.06E-01	μSv/h	3.33E-02	cps	7.19E-02	cps	60	21	0	21760	24.10.19	13:32
43762.5647	2.17E+02	cps	2.33E+02	cps	4.87E-01	μSv/h	5.63E-01	μSv/h	5.00E-02	cps	6.75E-02	cps	60	21	0	21760	24.10.19	13:33
43762.56539	2.26E+02	cps	2.42E+02	cps	5.16E-01	μSv/h	5.97E-01	μSv/h	5.00E-02	cps	6.97E-02	cps	60	21	0	21760	24.10.19	13:34
43762.56609	2.50E+02	cps	3.32E+02	cps	5.73E-01	μSv/h	6.87E-01	μSv/h	1.67E-02	cps	3.70E-02	cps	60	21	0	21760	24.10.19	13:35
43762.56678	2.76E+02	cps	3.14E+02	cps	6.47E-01	μSv/h	7.45E-01	μSv/h	8.33E-02	cps	7.64E-02	cps	60	21	0	21760	24.10.19	13:36
43762.56748	2.34E+02	cps	2.69E+02	cps	5.55E-01	μSv/h	6.51E-01	μSv/h	1.00E-01	cps	1.60E-01	cps	60	21	0	21760	24.10.19	13:37
43762.56817	2.19E+02	cps	2.69E+02	cps	5.00E-01	μSv/h	5.47E-01	μSv/h	1.83E-01	cps	1.93E-01	cps	60	21	0	21760	24.10.19	13:38
43762.56887	2.37E+02	cps	2.57E+02	cps	5.13E-01	μSv/h	5.24E-01	μSv/h	1.67E-02	cps	1.73E-01	cps	60	21	0	21760	24.10.19	13:39
43762.56956	3.03E+02	cps	4.33E+02	cps	7.14E-01	μSv/h	1.01E+00	μSv/h	3.33E-02	cps	6.54E-02	cps	60	21	0	21760	24.10.19	13:40
43762.57026	3.44E+02	cps	5.26E+02	cps	8.45E-01	μSv/h	1.24E+00	μSv/h	5.00E-02	cps	6.94E-02	cps	60	21	0	21760	24.10.19	13:41
43762.57095	2.63E+02	cps	2.87E+02	cps	6.55E-01	μSv/h	7.21E-01	μSv/h	5.00E-02	cps	5.54E-02	cps	60	21	0	21760	24.10.19	13:42
43762.57166	2.74E+02	cps	3.83E+02	cps	6.73E-01	μSv/h	8.61E-01	μSv/h	8.33E-02	cps	8.70E-02	cps	60	21	0	21760	24.10.19	13:43
43762.57235	2.60E+02	cps	3.00E+02	cps	5.88E-01	μSv/h	6.22E-01	μSv/h	1.67E-01	cps	1.61E-01	cps	60	21	0	21760	24.10.19	13:44
43762.57304	2.56E+02	cps	3.20E+02	cps	6.02E-01	μSv/h	7.19E-01	μSv/h	1.33E-01	cps	1.53E-01	cps	60	20	0	21760	24.10.19	13:45
43762.57373	2.52E+02	cps	2.94E+02	cps	5.91E-01	μSv/h	6.25E-01	μSv/h	1.50E-01	cps	1.75E-01	cps	60	20	0	21760	24.10.19	13:46
43762.57443	2.69E+02	cps	3.39E+02	cps	6.28E-01	μSv/h	9.24E-01	μSv/h	1.00E-01	cps	1.59E-01	cps	60	20	0	21760	24.10.19	13:47
43762.57513	2.39E+02	cps	2.88E+02	cps	5.34E-01	μSv/h	6.46E-01	μSv/h	1.33E-01	cps	1.67E-01	cps	60	20	0	21760	24.10.19	13:48
43762.57582	2.25E+02	cps	2.72E+02	cps	5.18E-01	μSv/h	5.94E-01	μSv/h	1.17E-01	cps	2.79E-01	cps	60	20	0	21760	24.10.19	13:49
43762.57652	2.42E+02	cps	2.56E+02	cps	5.49E-01	μSv/h	6.23E-01	μSv/h	1.17E-01	cps	1.96E-01	cps	60	20	0	21760	24.10.19	13:50
43762.57721	2.31E+02	cps	2.43E+02	cps	4.99E-01	μSv/h	5.72E-01	μSv/h	8.33E-02	cps	1.42E-01	cps	60	20	0	21760	24.10.19	13:51
43762.57791	2.15E+02	cps	2.47E+02	cps	4.98E-01	μSv/h	5.97E-01	μSv/h	6.67E-02	cps	9.22E-02	cps	60	20	0	21760	24.10.19	13:52
43762.5786	1.82E+02	cps	1.92E+02	cps	3.96E-01	μSv/h	4.14E-01	μSv/h	5.00E-02	cps	8.17E-02	cps	60	20	0	21760	24.10.19	13:53
43762.57929	1.89E+02	cps	2.04E+02	cps	4.04E-01	μSv/h	4.30E-01	μSv/h	6.67E-02	cps	8.63E-02	cps	60	20	0	21760	24.10.19	13:54
43762.57998	2.00E+02	cps	2.20E+02	cps	4.34E-01	μSv/h	4.97E-01	μSv/h	1.00E-01	cps	1.02E-01	cps	60	20	0	21760	24.10.19	13:55
43762.58068	1.99E+02	cps	2.28E+02	cps	4.36E-01	μSv/h	4.80E-01	μSv/h	6.67E-02	cps	1.07E-01	cps	60	20	0	21760	24.10.19	13:56
43762.58138	2.01E+02	cps	2.16E+02	cps	4.76E-01	μSv/h	5.45E-01	μSv/h	8.33E-02	cps	9.96E-02	cps	60	20	0	21760	24.10.19	13:57
43762.58207	1.87E+02	cps	2.25E+02	cps	4.03E-01	μSv/h	5.15E-01	μSv/h	3.33E-02	cps	9.05E-02	cps	60	20	0	21760	24.10.19	13:58
43762.58276	1.63E+02	cps	1.87E+02	cps	3.38E-01	μSv/h	3.55E-01	μSv/h	6.67E-02	cps	9.50E-02	cps	60	20	0	21760	24.10.19	13:59
43762.58346	1.84E+02	cps	2.05E+02	cps	3.85E-01	μSv/h	4.33E-01	μSv/h	1.33E-01	cps	1.50E-01	cps	60	20	0	21760	24.10.19	14:00
43762.58416	1.82E+02	cps	2.00E+02	cps	4.17E-01	μSv/h	4.32E-01	μSv/h	1.50E-01	cps	1.49E-01	cps	60	20	0	21760	24.10.19	14:01
43762.58485	1.95E+02	cps	2.09E+02	cps	4.34E-01	μSv/h	5.18E-01	μSv/h	1.33E-01	cps	2.62E-01	cps	60	20	0	21760	24.10.19	14:02
43762.58554	1.86E+02	cps	2.15E+02	cps	3.98E-01	μSv/h	5.01E-01	μSv/h	1.00E-01	cps	2.46E-01	cps	60	20	0	21760	24.10.19	14:03
43762.58624	1.95E+02	cps	2.14E+02	cps	4.30E-01	μSv/h	5.99E-01	μSv/h	6.67E-02	cps	1.21E-01	cps	60	20	0	21760	24.10.19	14:04
43762.58693	2.07E+02	cps	2.46E+02	cps	4.58E-01	μSv/h	5.12E-01	μSv/h	8.33E-02	cps	1.16E-01	cps	60	20	0	21760	24.10.19	14:05
43762.58763	2.28E+02	cps	2.55E+02	cps	4.86E-01	μSv/h	5.01E-01	μSv/h	0.00E+00	cps	7.25E-02	cps	60	20	0	21760	24.10.19	14:06
43762.58832	1.93E+02	cps	2.50E+02	cps	4.01E-01	μSv/h	5.10E-01	μSv/h	1.00E-01	cps	1.04E-01	cps	60	20	0	21760	24.10.19	14:07
43762.58902	2.14E+02	cps	2.66E+02	cps	4.53E-01	μSv/h	6.59E-01	μSv/h	1.17E-01	cps	1.36E-01	cps	60	20	0	21760	24.10.19	14:08
43762.58971	2.11E+02	cps	2.29E+02	cps	4.45E-01	μSv/h	4.76E-01	μSv/h	1.33E-01	cps	1.36E-01	cps	60	19	0	21760	24.10.19	14:09
43762.59039	2.25E+02	cps	2.37E+02	cps	5.09E-01	μSv/h	5.40E-01	μSv/h	1.50E-01	cps	1.70E-01	cps	60	19	0	21760	24.10.19	14:10
43762.5911	2.21E+02	cps	2.51E+02	cps	4.78E-01	μSv/h	6.20E-01	μSv/h	1.17E-01	cps	1.61E-01	cps	60	19	0	21760	24.10.19	14:11
43762.59179	1.85E+02	cps	2.00E+02	cps	3.99E-01	μSv/h	4.58E-01	μSv/h	8.33E-02	cps	1.38E-01	cps	60	19	0	21760	24.10.19	14:12
43762.59249	1.88E+02	cps	2.03E+02	cps	3.95E-01	μSv/h	4.21E-01	μSv/h	5.00E-02	cps	1.01E-01	cps	60	19	0	21760	24.10.19	14:13
43762.59318	1.90E+02	cps	2.07E+02	cps	4.14E-01	μSv/h	4.45E-01	μSv/h	6.67E-02	cps	7.17E-02	cps	60	19	0	21760	24.10.19	14:14
43762.59388	1.98E+02	cps	2.23E+02	cps	4.34E-01	μSv/h	6.38E-01	μSv/h	6.67E-02	cps	8.86E-02	cps	60	19	0	21760	24.10.19	14:15
43762.59457	1.96E+02	cps	2.16E+02	cps	4.38E-01	μSv/h	5.90E-01	μSv/h	8.33E-02	cps	9.28E-02	cps	60	19	0	21760	24.10.19	14:16
43762.59527	1.89E+02	cps	2.08E+02	cps	4.16E-01	μSv/h	4.52E-01	μSv/h	1.67E-01	cps	1.60E-01	cps	60	19	0	21760	24.10.19	14:17
43762.59596	1.92E+02	cps	2.02E+02	cps	4.26E-01	μSv/h	4.42E-01	μSv/h	8.33E-02	cps	1.50E-01	cps	60	19	0	21760	24.10.19	14:18
43762.59666	1.72E+02	cps	1.89E+02	cps	3.68E-01	μSv/h	4.33E-01	μSv/h	5.00E-02	cps	9.25E-02	cps	60	19	0	21760	24.10.19	14:19
43762.59734	1.68E+02	cps	1.94E+02	cps	3.65E-01	μSv/h	4.43E-01	μSv/h	1.50E-01	cps	1.43E-01	cps	60	19	0	21760	24.10.19	14:20
43762.59804	1.77E+02	cps	1.91E+02	cps	3.67E-01	μSv/h	4.25E-01	μSv/h	5.00E-02	cps	1.46E-01	cps	60	19	0	21760	24.10.19	14:21
43762.59874	1.68E+02	cps	2.23E+02	cps	3.50E-01	μSv/h	5.05E-01	μSv/h	1.00E-01	cps	1.37E-01	cps	60	19	0	21760	24.10.19	14:22
43762.5994	1.71E+02	cps	1.84E+02	cps	3.50E-01	μSv/h	4.02E-01	μSv/h	6.67E-02	cps	8.72E-02	cps	60	19	0	21760	24.10.19	14:23
43762.60012	1.72E+02	cps	1.84E+02	cps	3.68E-01	μSv/h	4.24E-01	μSv/h	1.33E-01	cps	1.39E-01	cps	60	19	0	21760	24.10.19	14:24
43762.60082	1.65E+02	cps	1.74E+02	cps	3.40E-01	μSv/h	4.05E-01	μSv/h	3.33E-02	cps	1.14E-01	cps	60	19	0	21760	24.10.19	14:25
43762.60152	1.73E+02	cps	1.92E+02	cps	3.63E-01	μSv/h	3.88E-01	μSv/h	1.17E-01	cps	1.40E-01	cps	60	19	0	21760	24.10.19	14:26
43762.60221	1.89E+02	cps	2.05E+02	cps	4.14E-01	μSv/h	4.59E-01	μSv/h	5.00E-02	cps	8.62E-02	cps	60	19	0	21760	24.10.19	14:27
43762.60291	1.92E+02	cps	2.28E+02	cps	4.27E-01	μSv/h	4.74E-01	μSv/h	6.67E-02	cps	9.09E-02	cps	60	19	0	21760	24.10.19	14:28
43762.60361	1.82E+02	cps	2.06E+02	cps	4.25E-01	μSv/h	5.53E-01	μSv/h	1.67E-02	cps	6.59E-02	cps	60	19	0	21760	24.10.19	14:29
43762.60431	1.78E+02	cps	2.05E+02	cps	3.96E-01	μSv/h	4.61E-01	μSv/h	1.50E-01	cps	1.60E-01	cps	60	19	0	21760	24.10.19	14:30
43762.605	1.82E+02	cps	1.97E+02	cps	4.15E-01	μSv/h	5.20E-01	μSv/h	8.33E-02	cps	1.33E-01	cps	60	19	0	21760	24.10.19	14:31
43762.60569	1.83E+02	cps	2.03E+02	cps	4.12E-01	μSv/h	5.61E-01	μSv/h	3.33E-02	cps	8.80E-02	cps	60	19	0	21760	24.10.19	14:32
43762.60639	1.87E+02	cps	2.08E+02	cps	4.30E-01	μSv/h	5.15E-01	μSv/h	6.67E-02	cps	8.52E-02	cps	60	19	0	21760	24.10.19	14:33
43762.60708	2.12E+02	cps	2.62E+02	cps	4.98E-01	μSv/h	6.27E-01	μSv/h	8.33E-02	cps	8.40E-02	cps	60	19	0	21760	24.10.19	14:34

Table S3-5. Data sheet for December 5, 2019

RadEye SPRD History	Version 1.42.0	30													Survey	
Date	Serial number	Version	Type	Unit GUnit N	Timer interval	Flag 1	Flag 2	Flag 3	Flag 4	Flag Unit G	Flag Unit N	Flag Type	Index Unit	HistCalMode	Average (μSv/h)	0.45
05/12/2019	05/12/2019	RadEye SPRD E1.16	Sv/h	cps	60	124	181	0	2056	261	0	48	5	0	Median (μSv/h)	0.44
															90th percentile	0.62
															Min	0.14
															Max	0.83
															Estimated annual dose rate (mSv/y)	3.8

Date	Mean G	Unit	Max G/Bgnd G	Unit	Mean DR-G[μSv/h]	Unit	Max DR[μSv/h]/nc	Unit	N[cps]	Unit	Max N/Bgnd N[cps]	Unit	M. time	Temperature	#	Status	Date [dd.mm.yy]	Time [hh:mm]
43804.42345	7.91E+01	cps	8.59E+01	cps	1.37E-01	μSv/h	1.52E-01	μSv/h	1.00E-01	cps	1.18E-01	cps	60	17	0	21760	05.12.19	10:09
43804.42413	1.31E+02	cps	2.02E+02	cps	2.54E-01	μSv/h	4.42E-01	μSv/h	6.67E-02	cps	1.05E-01	cps	60	17	0	21760	05.12.19	10:10
43804.42485	1.77E+02	cps	1.96E+02	cps	4.26E-01	μSv/h	4.99E-01	μSv/h	1.17E-01	cps	1.20E-01	cps	60	17	0	21760	05.12.19	10:11
43804.42552	2.02E+02	cps	2.33E+02	cps	5.13E-01	μSv/h	6.02E-01	μSv/h	8.33E-02	cps	1.20E-01	cps	60	17	0	21760	05.12.19	10:12
43804.42623	1.88E+02	cps	2.56E+02	cps	4.60E-01	μSv/h	5.35E-01	μSv/h	1.17E-01	cps	1.41E-01	cps	60	16	0	21760	05.12.19	10:13
43804.42693	1.40E+02	cps	1.70E+02	cps	3.02E-01	μSv/h	4.32E-01	μSv/h	8.33E-02	cps	1.12E-01	cps	60	16	0	21760	05.12.19	10:14
43804.42762	1.66E+02	cps	1.94E+02	cps	4.16E-01	μSv/h	5.46E-01	μSv/h	1.00E-01	cps	1.43E-01	cps	60	16	0	21760	05.12.19	10:15
43804.42833	1.80E+02	cps	2.06E+02	cps	4.66E-01	μSv/h	5.05E-01	μSv/h	1.00E-01	cps	1.29E-01	cps	60	16	0	21760	05.12.19	10:16
43804.42903	1.79E+02	cps	2.23E+02	cps	4.43E-01	μSv/h	5.19E-01	μSv/h	1.00E-01	cps	1.39E-01	cps	60	15	0	21760	05.12.19	10:17
43804.42972	1.59E+02	cps	2.24E+02	cps	3.29E-01	μSv/h	5.03E-01	μSv/h	1.00E-01	cps	1.23E-01	cps	60	15	0	21760	05.12.19	10:18
43804.43042	1.55E+02	cps	1.67E+02	cps	3.33E-01	μSv/h	3.64E-01	μSv/h	5.00E-02	cps	1.02E-01	cps	60	15	0	21760	05.12.19	10:19
43804.4311	1.59E+02	cps	1.68E+02	cps	3.37E-01	μSv/h	4.47E-01	μSv/h	5.00E-02	cps	7.70E-02	cps	60	14	0	21760	05.12.19	10:20
43804.43181	1.61E+02	cps	1.75E+02	cps	3.39E-01	μSv/h	3.71E-01	μSv/h	5.00E-02	cps	7.92E-02	cps	60	14	0	21760	05.12.19	10:21
43804.4325	2.25E+02	cps	2.78E+02	cps	5.19E-01	μSv/h	5.83E-01	μSv/h	8.33E-02	cps	9.17E-02	cps	60	14	0	21760	05.12.19	10:22
43804.43319	1.92E+02	cps	2.48E+02	cps	4.34E-01	μSv/h	4.81E-01	μSv/h	1.00E-01	cps	1.13E-01	cps	60	14	0	21760	05.12.19	10:23
43804.43388	1.85E+02	cps	2.41E+02	cps	3.79E-01	μSv/h	4.67E-01	μSv/h	1.00E-01	cps	1.39E-01	cps	60	14	0	21760	05.12.19	10:24
43804.43458	2.73E+02	cps	3.14E+02	cps	6.25E-01	μSv/h	7.11E-01	μSv/h	3.33E-02	cps	7.76E-02	cps	60	14	0	21760	05.12.19	10:25
43804.43528	2.12E+02	cps	2.75E+02	cps	4.44E-01	μSv/h	6.28E-01	μSv/h	5.00E-02	cps	6.72E-02	cps	60	14	0	21760	05.12.19	10:26
43804.43597	2.05E+02	cps	2.24E+02	cps	4.56E-01	μSv/h	5.13E-01	μSv/h	1.00E-01	cps	1.40E-01	cps	60	13	0	21760	05.12.19	10:27
43804.43668	2.21E+02	cps	2.49E+02	cps	4.58E-01	μSv/h	4.83E-01	μSv/h	3.33E-02	cps	1.07E-01	cps	60	13	0	21760	05.12.19	10:28
43804.43737	2.19E+02	cps	2.53E+02	cps	4.69E-01	μSv/h	5.27E-01	μSv/h	6.67E-02	cps	7.08E-02	cps	60	13	0	21760	05.12.19	10:29
43804.43807	2.19E+02	cps	2.49E+02	cps	4.69E-01	μSv/h	5.40E-01	μSv/h	1.00E-01	cps	9.19E-02	cps	60	13	0	21760	05.12.19	10:30
43804.43876	2.34E+02	cps	2.50E+02	cps	4.90E-01	μSv/h	5.17E-01	μSv/h	3.33E-02	cps	1.16E-01	cps	60	13	0	21760	05.12.19	10:31
43804.43946	2.20E+02	cps	2.31E+02	cps	4.81E-01	μSv/h	5.09E-01	μSv/h	1.50E-01	cps	1.15E-01	cps	60	13	0	21760	05.12.19	10:32
43804.44015	2.54E+02	cps	3.20E+02	cps	5.77E-01	μSv/h	6.36E-01	μSv/h	3.33E-02	cps	1.15E-01	cps	60	12	0	21760	05.12.19	10:33
43804.44083	2.69E+02	cps	3.20E+02	cps	6.05E-01	μSv/h	7.15E-01	μSv/h	1.00E-01	cps	9.59E-02	cps	60	12	0	21760	05.12.19	10:34
43804.44154	2.59E+02	cps	3.28E+02	cps	6.00E-01	μSv/h	7.44E-01	μSv/h	6.67E-02	cps	1.05E-01	cps	60	12	0	21760	05.12.19	10:35
43804.44223	2.29E+02	cps	2.53E+02	cps	5.06E-01	μSv/h	5.45E-01	μSv/h	5.00E-02	cps	1.01E-01	cps	60	12	0	21760	05.12.19	10:36
43804.44293	3.51E+02	cps	5.55E+02	cps	8.28E-01	μSv/h	1.33E+00	μSv/h	6.67E-02	cps	7.91E-02	cps	60	12	0	21760	05.12.19	10:37
43804.44362	2.99E+02	cps	3.57E+02	cps	7.23E-01	μSv/h	8.08E-01	μSv/h	1.00E-01	cps	1.16E-01	cps	60	12	0	21760	05.12.19	10:38
43804.44433	2.57E+02	cps	2.82E+02	cps	6.37E-01	μSv/h	7.82E-01	μSv/h	1.50E-01	cps	1.76E-01	cps	60	11	0	21760	05.12.19	10:39
43804.44502	2.78E+02	cps	4.07E+02	cps	6.44E-01	μSv/h	9.29E-01	μSv/h	6.67E-02	cps	1.67E-01	cps	60	11	0	21760	05.12.19	10:40
43804.44571	2.67E+02	cps	3.04E+02	cps	6.10E-01	μSv/h	6.66E-01	μSv/h	8.33E-02	cps	9.86E-02	cps	60	11	0	21760	05.12.19	10:41
43804.44641	2.72E+02	cps	3.22E+02	cps	6.20E-01	μSv/h	7.05E-01	μSv/h	3.33E-02	cps	8.42E-02	cps	60	11	0	21760	05.12.19	10:42
43804.4471	2.49E+02	cps	2.62E+02	cps	5.45E-01	μSv/h	6.33E-01	μSv/h	1.00E-01	cps	1.11E-01	cps	60	11	0	21760	05.12.19	10:43
43804.4478	2.79E+02	cps	3.59E+02	cps	6.70E-01	μSv/h	8.13E-01	μSv/h	8.33E-02	cps	1.08E-01	cps	60	11	0	21760	05.12.19	10:44
43804.4485	2.35E+02	cps	2.66E+02	cps	5.07E-01	μSv/h	6.20E-01	μSv/h	5.00E-02	cps	1.25E-01	cps	60	10	0	21760	05.12.19	10:45
43804.44919	2.44E+02	cps	2.93E+02	cps	5.49E-01	μSv/h	6.07E-01	μSv/h	3.33E-02	cps	5.50E-02	cps	60	10	0	21760	05.12.19	10:46
43804.44988	2.51E+02	cps	2.71E+02	cps	5.60E-01	μSv/h	6.16E-01	μSv/h	1.17E-01	cps	1.04E-01	cps	60	10	0	21760	05.12.19	10:47
43804.45058	2.35E+02	cps	2.51E+02	cps	5.21E-01	μSv/h	5.70E-01	μSv/h	3.33E-02	cps	8.85E-02	cps	60	10	0	21760	05.12.19	10:48
43804.45127	2.60E+02	cps	2.95E+02	cps	6.29E-01	μSv/h	7.30E-01	μSv/h	3.33E-02	cps	5.61E-02	cps	60	10	0	21760	05.12.19	10:49
43804.45197	3.08E+02	cps	3.49E+02	cps	7.36E-01	μSv/h	9.91E-01	μSv/h	3.33E-02	cps	5.93E-02	cps	60	10	0	21760	05.12.19	10:50
43804.45266	3.00E+02	cps	3.52E+02	cps	7.19E-01	μSv/h	9.80E-01	μSv/h	8.33E-02	cps	7.93E-02	cps	60	10	0	21760	05.12.19	10:51
43804.45336	2.39E+02	cps	2.57E+02	cps	5.23E-01	μSv/h	5.66E-01	μSv/h	6.67E-02	cps	9.21E-02	cps	60	10	0	21760	05.12.19	10:52
43804.45405	2.04E+02	cps	2.46E+02	cps	4.59E-01	μSv/h	5.63E-01	μSv/h	3.33E-02	cps	7.58E-02	cps	60	10	0	21760	05.12.19	10:53
43804.45475	1.91E+02	cps	2.02E+02	cps	3.90E-01	μSv/h	4.16E-01	μSv/h	6.67E-02	cps	7.75E-02	cps	60	10	0	21760	05.12.19	10:54
43804.45544	2.02E+02	cps	2.17E+02	cps	4.37E-01	μSv/h	5.50E-01	μSv/h	5.00E-02	cps	6.35E-02	cps	60	10	0	21760	05.12.19	10:55
43804.45612	2.05E+02	cps	2.18E+02	cps	4.40E-01	μSv/h	5.26E-01	μSv/h	1.17E-01	cps	1.05E-01	cps	60	10	0	21760	05.12.19	10:56
43804.45683	2.02E+02	cps	2.26E+02	cps	4.44E-01	μSv/h	5.19E-01	μSv/h	6.67E-02	cps	1.09E-01	cps	60	10	0	21760	05.12.19	10:57
43804.4575	1.73E+02	cps	2.33E+02	cps	3.55E-01	μSv/h	4.83E-01	μSv/h	0.00E+00	cps	6.97E-02	cps	60	10	0	21760	05.12.19	10:58
43804.45822	1.57E+02	cps	1.92E+02	cps	3.06E-01	μSv/h	3.90E-01	μSv/h	5.00E-02	cps	5.73E-02	cps	60	10	0	21760	05.12.19	10:59
43804.45891	1.76E+02	cps	2.13E+02	cps	3.42E-01	μSv/h	3.89E-01	μSv/h	1.00E-01	cps	9.89E-02	cps	60	10	0	21760	05.12.19	11:00
43804.4596	1.76E+02	cps	2.07E+02	cps	3.64E-01	μSv/h	4.32E-01	μSv/h	0.00E+00	cps	8.51E-02	cps	60	10	0	21760	05.12.19	11:01
43804.46028	1.84E+02	cps	2.10E+02	cps	3.87E-01	μSv/h	4.13E-01	μSv/h	6.67E-02	cps	7.01E-02	cps	60	10	0	21760	05.12.19	11:02
43804.461	1.76E+02	cps	2.04E+02	cps	3.88E-01	μSv/h	4.86E-01	μSv/h	6.67E-02	cps	9.21E-02	cps	60	10	0	21760	05.12.19	11:03
43804.46169	1.78E+02	cps	1.94E+02	cps	3.65E-01	μSv/h	4.70E-01	μSv/h	1.00E-01	cps	1.31E-01	cps	60	10	0	21760	05.12.19	11:04
43804.46238	2.07E+02	cps	2.30E+02	cps	4.59E-01	μSv/h	4.93E-01	μSv/h	5.00E-02	cps	9.95E-02	cps	60	10	0	21760	05.12.19	11:05
43804.46308	2.19E+02	cps	2.41E+02	cps	4.88E-01	μSv/h	5.50E-01	μSv/h	3.33E-02	cps	6.63E-02	cps	60	10	0	21760	05.12.19	11:06
43804.46377	2.04E+02	cps	2.44E+02	cps	4.55E-01	μSv/h	5.36E-01	μSv/h	1.33E-01	cps	1.19E-01	cps	60	10	0	21760	05.12.19	11:07
43804.46447	2.06E+02	cps	2.96E+02	cps	4.56E-01	μSv/h	6.67E-01	μSv/h	8.33E-02	cps	1.10E-01	cps	60	10	0	21760	05.12.19	11:08
43804.46516	2.06E+02	cps	2.36E+02	cps	4.56E-01	μSv/h	6.48E-01	μSv/h	5.00E-02	cps	1.32E-01	cps	60	10	0	21760	05.12.19	11:09
43804.46586	2.08E+02	cps	2.30E+02	cps	4.38E-01	μSv/h	4.60E-01	μSv/h	1.17E-01	cps	1.48E-01	cps	60	10	0	21760	05.12.19	11:10
43804.46655	2.31E+02	cps	2.60E+02	cps	5.01E-01	μSv/h	5.25E-01	μSv/h	5.00E-02	cps	1.18E-01	cps	60	10	0	21760	05.12.19	11:11
43804.46725	1.94E+02	cps	2.28E+02	cps	4.13E-01	μSv/h	5.08E-01	μSv/h	1.00E-01	cps	1.12E-01	cps	60	11	0	21760	05.12.19	11:12
43804.46794	1.82E+02	cps	1.93E+02	cps	3.99E-01	μSv/h	4.11E-01	μSv/h	8.33E-02	cps	1.21E-01	cps	60	11	0	21760	05.12.19	11:13
43804.46863	1.81E+02	cps	1.96E+02	cps	4.25E-01	μSv/h	4.52E-01	μSv/h	1.00E-01	cps	1.21E-01	cps	60	11	0	21760	05.12.19	11:14
43804.46933	1.82E+02	cps	2.02E+02	cps	4.02E-01	μSv/h	4.44E-01	μSv/h	5.00E-02	cps	1.05E-01	cps	60	11	0	21760	05.12.19	11:15
43804.47002	1.88E+02	cps	2.15E+02	cps	4.16E-01	μSv/h	4.71E-01	μSv/h	3.33E-02	cps	7.53E-02	cps	60	11	0	21760	05.12.19	11:16
43804.47072	2.00E+02	cps	2.16E+02	cps	4.42E-01	μSv/h	4.76E-01	μSv/h	6.67E-02	cps	8.61E-02	cps	60	11	0	21760	05.12.19	11:17
43804.4714	1.90E+02	cps	2.23E+02	cps	4.15E-01	μSv/h	4.35E-01	μSv/h	5.00E-02	cps	6.88E-02	cps	60	11	0	21760	05.12.19	11:18
43804.47211	1.97E+02	cps	2.14E+02	cps	4.28E-01	μSv/h	4.96E-01	μSv/h	6.67E-02	cps	1.03E-01	cps	60	11	0	21760	05.12.19	11:19
43804.4728	1.74E+02	cps	1.90E+02	cps	3.77E-01	μSv/h	4.07E-01	μSv/h	5.00E-02	cps	7.60E-02	cps	60	11	0	21760	05.12.19	11:20
43804.4735	1.64E+02	cps	2.07E+02	cps	3.40E-01	μSv/h	4.21E-01	μSv/h	8.33E-02	cps	8.92E-02	cps	60	11	0	21760	05.12.19	11:21
43804.47419	1.81E+02	cps	2.03E+02	cps	3.91E-01	μSv/h	4.22E-01	μSv/h	6.67E-02	cps	1.24E-01	cps	60	11	0	21760	05.12.19	11:22
43804.47487	1.78E+02	cps	1.92E+02	cps	3.65E-01	μSv/h	4.38E-01	μSv/h	1.00E-01	cps	1.03E-01	cps	60	11	0	21760	05.12.19	11:23
43804.47558	1.64E+02	cps	1.75E+02	cps	3.27E-01	μSv/h	3.63E-01	μSv/h	1.00E-01	cps	1.20E-01	cps	60	12	0	21760	05.12.19	11:24
43804.47627	1.66E+02	cps	2.00E+02	cps	3.50E-01	μSv/h	4.18E-01	μSv/h	8.33E-02	cps	1.06E-01	cps	60	12	0	21760	05.12.19	11:25
43804.47697	1.87E+02	cps	1.98E+02	cps	4.21E-01	μSv/h	4.73E-01	μSv/h	1.33E-01	cps	2.63E-01	cps	60	12	0	21760	05.12.19	11:26
43804.47765	1.90E+02	cps	2.18E+02	cps	4.34E-01	μSv/h	5.01E-01	μSv/h	1.67E-02	cps	1.55E-01	cps	60	12	0	21760	05.12.19	11:27
43804.47836	1.75E+02	cps	1.98E+02	cps	3.63E-01	μSv/h	3.94E-01	μSv/h	1.67E-02	cps	4.67E-02	cps	60	12	0	21760	05.12.19	11:28
43804.47905	1.68E+02	cps	1.87E+02	cps	3.81E-01	μSv/h	4.20E-01	μSv/h	1.00E-01	cps	1.02E-01	cps	60	12	0	21760	05.12.19	11:29
43804.47975	1.65E+02	cps	1.76E+02	cps	3.52E-01	μSv/h	3.80E-01	μSv/h	6.67E-02	cps	1.02E-01	cps	60	12	0	21760	05.12.19	11:30
43804.48044	1.76E+02	cps	1.90E+02	cps	3.84E-01	μSv/h	4.68E-01	μSv/h	6.67E-02	cps	1.07E-01	cps	60	12	0	21760	05.12.19	11:31
43804.48112	1.94E+02	cps	2.24E+02	cps	4.65E-01	μSv/h	5.83E-01	μSv/h	1.17E-01	cps	1.12E-01	cps	60	12	0	21760	05.12.19	11:32
43804.48183	1.75E+02	cps	1.87E+02	cps	3.76E-01	μSv/h	4.77E-01	μSv/h	6.67E-02	cps	1.21E-01	cps	60	12	0	21760	05.12.19	11:33
43804.48252	1.87E+02	cps	2.21E+02	cps	4.14E-01	μSv/h	4.61E-01	μSv/h	6.67E-02	cps	9.16E-02	cps	60	12	0	21760	05.12.19	11:34
43804.48322	1.77E+02	cps	2.06E+02	cps	3.88E-01	μSv/h	4.61E-01	μSv/h	1.33E-01	cps	1.19E-01	cps	60	12	0	21760	05.12.19	11:35
43804.48391	1.71E+02	cps	2.04E+02	cps	3.71E-01	μSv/h	4.36E-01	μSv/h	5.00E-02	cps	1.13E-01	cps	60	12	0	21760	05.12.19	11:36
43804.4846	1.69E+02	cps	1.99E+02	cps	3.79E-01	μSv/h	5.47E-01	μSv/h	6.67E-02	cps	9.57E-02	cps	60	12	0	21760	05.12.19	11:37
43804.4853	1.84E+02	cps	2.09E+02	cps	4.18E-01	μSv/h	4.77E-01	μSv/h	3.33E-02	cps	6.53E-02	cps	60	12	0	21760	05.12.19	11:38
43804.486	1.93E+02	cps	2.06E+02	cps	4.52E-01	μSv/h	5.04E-01	μSv/h	5.00E-02	cps	6.44E-02	cps	60	12	0	21760	05.12.19	11:39
43804.48668	2.03E+02	cps	2.20E+02	cps	4.56E-01	μSv/h	4.84E-01	μSv/h	1.17E-01	cps	1.26E-01	cps	60	12	0	21760	05.12.19	11:40
43804.48738	1.64E+02	cps	2.07E+02	cps	3.78E-01	μSv/h	4.80E-01	μSv/h	1.00E-01	cps	1.22E-01	cps	60	12	0	21760	05.12.19	11:41

Fig S3-1. Chronological changes in ambient dose rates measured using an SPRDJ dosimeter along the Mt Okura hiking trail

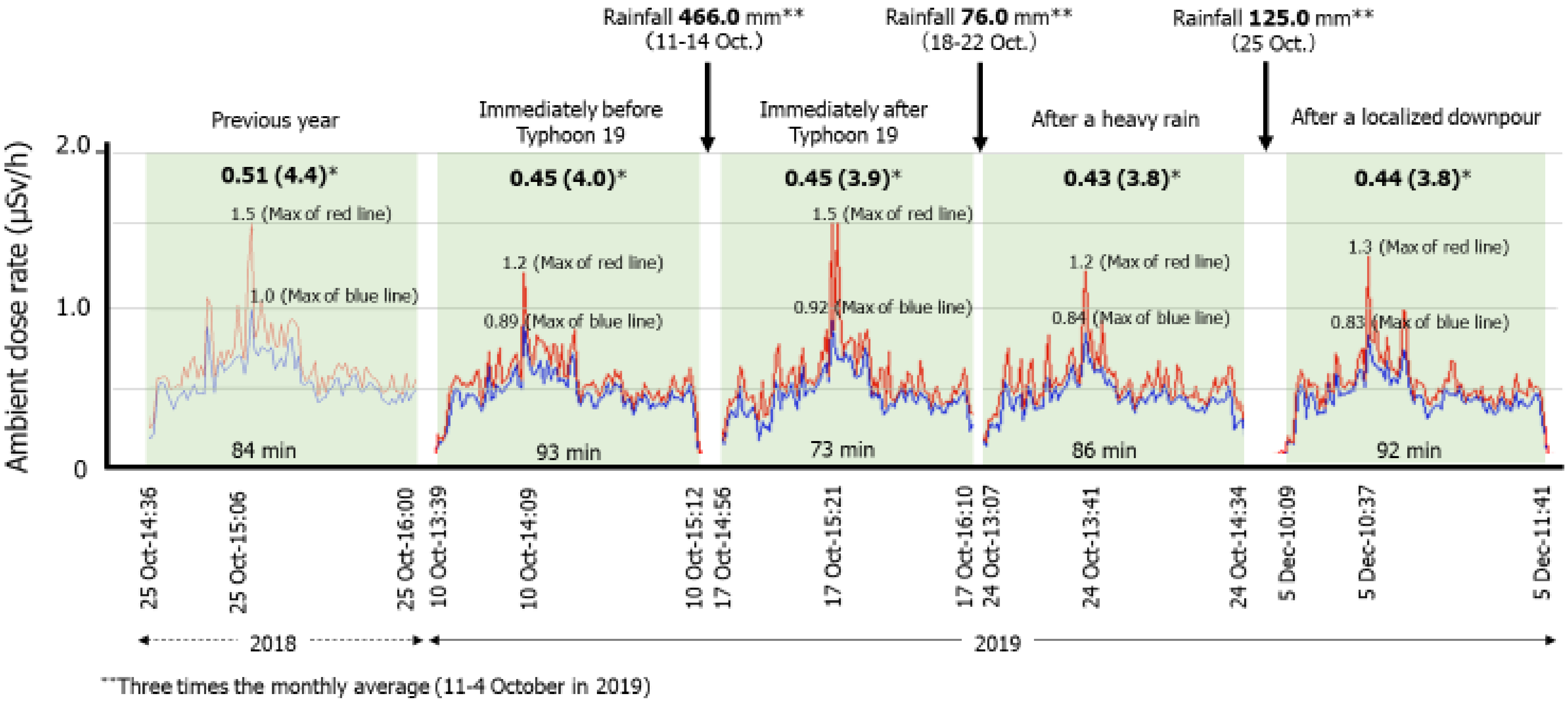

Fig S3-1 shows the variation in ambient dose rates, based on the data collected by walking survey using an SPRDJ dosimeter in 2018-2019. The red line shows the maximum ambient dose rates at each measuring point. The blue line shows the average ambient dose rates per minute. The single asterisk shows the median µSv/h value obtained using the SPRDJ dosimeter (the estimated annual dose rate in mSv/y is given in brackets). The double asterisk shows rainfall records associated with Typhoon 19 and downpours (JMA, Tokyo, Japan; data available from: http://www.data.jma.go.jp/obd/stats/etrn/view/daily_a1.php?prec_no=36&block_no=1129&year=2019&month=10&day=&view=). Values above the X-axis indicate the time taken to complete the hike.
